# Artificial intelligence-driven multi-omics approaches in Alzheimer's disease: Progress, challenges, and future directions

**DOI:** 10.1016/j.apsb.2025.07.030

**Published:** 2025-07-25

**Authors:** Fang Ren, Jing Wei, Qingxin Chen, Mengling Hu, Lu Yu, Jianing Mi, Xiaogang Zhou, Dalian Qin, Jianming Wu, Anguo Wu

**Affiliations:** a Chongqing Key Laboratory of Sichuan-Chongqing Co-construction for Diagnosis and Treatment of Infectious Diseases Integrated Traditional Chinese and Western Medicine, Chongqing Traditional Chinese Medicine Hospital, Chongqing 400021, China; b State Key Laboratory of Traditional Chinese Medicine Syndrome, the Second Affiliated Hospital of Guangzhou University of Chinese Medicine, Guangzhou 510120, China; c Sichuan Key Medical Laboratory of New Drug Discovery and Drugability Evaluation, Key Laboratory of Medical Electrophysiology of Ministry of Education, School of Pharmacy, Department of Cardiology, Department of Ophthalmology, the Affiliated Hospital of Southwest Medical University, Southwest Medical University, Luzhou 646000, China; d Department of Pharmacy, Guang'an People's Hospital, Guangan 638550, China

**Keywords:** Alzheimer's disease, Artificial intelligence, Multi-omics, Biomarkers, Early detection, Personalized treatment, Drug discovery, Pathological mechanisms, Machine learning, Deep learning

## Abstract

Alzheimer's disease (AD) is a progressive neurodegenerative disorder characterized by cognitive decline and memory loss, with few effective treatments currently available. The multifactorial nature of AD, shaped by genetic, environmental, and biological factors, complicates both research and clinical management. Recent advances in artificial intelligence (AI) and multi-omics technologies provide new opportunities to elucidate the molecular mechanisms of AD and identify early biomarkers for diagnosis and prognosis. AI-driven approaches such as machine learning, deep learning, and network-based models have enabled the integration of large-scale genomic, transcriptomic, proteomic, metabolomic, and microbiomic datasets. These efforts have facilitated the discovery of novel molecular signatures and therapeutic targets. Methods including deep belief networks and joint deep semi-non-negative matrix factorization have contributed to improvements in disease classification and patient stratification. However, ongoing challenges remain. These include data heterogeneity, limited interpretability of complex models, a lack of large and diverse datasets, and insufficient clinical validation. The absence of standardized multi-omics data processing methods further restricts progress. This review systematically summarizes recent advances in AI-driven multi-omics research in AD, highlighting achievements in early diagnosis and biomarker discovery while discussing limitations and future directions needed to advance these approaches toward clinical application.

## Graphical abstract

Artificial intelligence-driven multi-omics approaches in Alzheimer's disease integrate machine learning, deep learning, and network-based methods for biomarker identification, disease mechanism exploration, early diagnosis, patient stratification, and targeted drug development.

**Figure:**
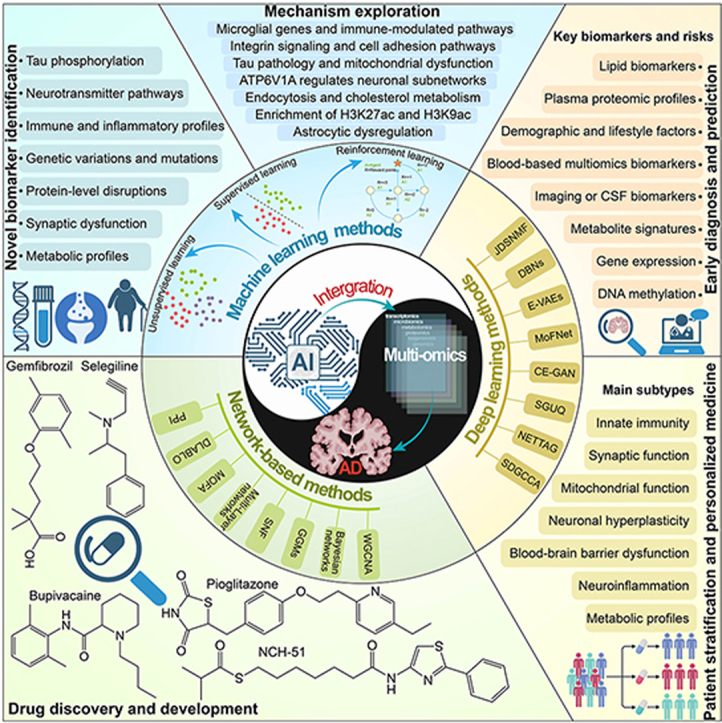

## 1 Introduction

Alzheimer's disease (AD) is the most prevalent cause of dementia and poses an increasing threat to global public health, especially with aging populations worldwide^1^. The disease is characterized by progressive cognitive decline, memory impairment, and complex neuropathological changes including amyloid beta (A*β*) plaque deposition, neurofibrillary tangles (NFTs) formed by hyperphosphorylated tau, chronic neuroinflammation, and extensive neuronal loss^2^. Despite extensive research, current therapeutic options for AD remain largely symptomatic and do not halt or reverse disease progression. This underscores the urgent need for novel strategies focused on early detection and personalized interventions.

Recent advances in multi-omics technologies, such as genomics, transcriptomics, proteomics, metabolomics, and microbiomics, enable comprehensive profiling of biological changes across multiple molecular layers in AD^3^. These technologies hold great promise for unraveling the complex molecular networks underlying AD pathogenesis. They enable the identification of novel biomarkers for early diagnosis and prognosis, as well as the discovery of new therapeutic targets. However, multi-omics data are inherently high-dimensional, heterogeneous, and noisy, often generated across disparate platforms. These characteristics significantly complicate data integration and interpretation using traditional statistical methods^4^.

Artificial intelligence (AI), particularly machine learning and deep learning algorithms, has emerged as a transformative tool ideally suited to overcoming these challenges. AI techniques are well-suited for analyzing large-scale, complex datasets. They can extract latent features, identify nonlinear patterns, and integrate diverse data modalities in a comprehensive manner^5^. In AD research, AI-driven multi-omics approaches facilitate biomarker discovery, patient stratification, disease progression modeling, and drug target identification with unprecedented precision^6^. These capabilities position AI as a critical enabler for unlocking the full potential of multi-omics in advancing AD research and precision medicine.

This review provides a comprehensive overview of recent advances in AI-driven multi-omics applications in AD, highlighting key methodological innovations and emerging biological insights. It examines how AI techniques have been applied to address major challenges in multi-omics research, such as data heterogeneity, feature selection, and temporal complexity. Clinical translation barriers, including issues related to validation and regulatory approval, are also discussed. Finally, the review outlines future directions for integrating multi-omics data with clinical and neuroimaging information to facilitate earlier diagnosis, more precise patient stratification, and personalized therapeutic development. Overall, it offers a structured synthesis of the current landscape and future potential of AI-enhanced multi-omics in addressing the complexities of AD.

## 2 Pathological mechanisms and biomarkers of AD

### 2.1 Pathological mechanisms

**Figure 1:**
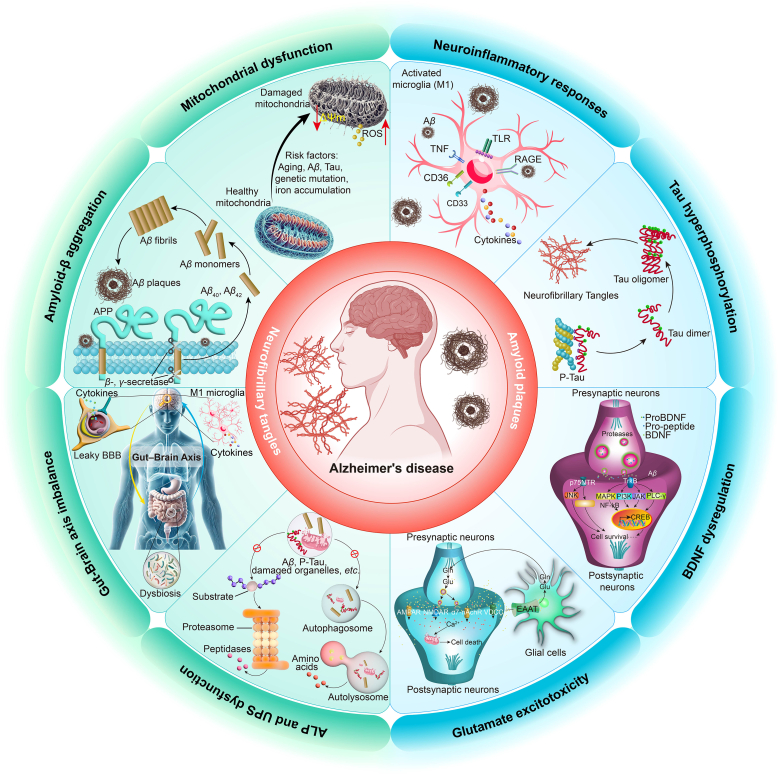
Key molecular mechanisms underlying Alzheimer's disease (AD) pathogenesis. The hallmark pathological features of AD are amyloid beta (A*β*) plaques and neurofibrillary tangles (NFTs). Several interconnected mechanisms contribute to AD progression: (1) A*β* aggregation: Abnormal cleavage of amyloid precursor protein (APP) forms A*β* monomers that aggregate into toxic oligomers, leading to plaque formation, synaptic dysfunction, and inflammation. (2) Tau hyperphosphorylation: Misfolded, hyperphosphorylated tau proteins form NFTs, disrupting neuronal function. (3) Neuroinflammatory responses: A*β* and tau accumulation activate microglia (M1), releasing pro-inflammatory cytokines (*e*.*g*., TNF-*α*) that worsen neuronal damage. (4) Mitochondrial dysfunction: Aging and oxidative stress damage mitochondria, increasing reactive oxygen species (ROS), accelerating neurodegeneration. (5) Glutamate excitotoxicity: Dysregulated glutamate signaling leads to excitotoxic neuronal damage and cell death *via* NMDA and AMPA receptor pathways. (6) Brain-derived neurotrophic factor (BDNF) dysregulation: Impaired BDNF signaling reduces neuronal survival and synaptic plasticity, contributing to cognitive decline. Pathways such as PI3K/AKT and MAPK/ERK are implicated in these processes. (7) Autophagy and proteasome dysfunction: Impaired autophagy-lysosome (ALP) and ubiquitin-proteasome (UPS) systems result in the accumulation of damaged proteins and organelles, including A*β* and tau, which further disrupt neuronal homeostasis. (8) Gut–brain axis imbalance: Gut dysbiosis promotes inflammation and blood-brain barrier (BBB) disruption, further aggravating AD pathology.

AD is a multifactorial neurodegenerative disorder marked by the interplay of A*β* accumulation, tau hyperphosphorylation, neuroinflammation, synaptic dysfunction, metabolic imbalance, oxidative stress, and mitochondrial impairment^7^^,^^8^. Increasing evidence also implicates alterations in the gut–brain axis in its pathogenesis^9^. These interconnected mechanisms collectively drive the onset and progression of the disease (Fig. 1)^2^. With the emergence of AI-integrated multi-omics platforms, computational approaches have been increasingly applied across these research domains to disentangle molecular interdependencies, identify latent variables, and construct models that capture disease evolution and region-specific pathology.

#### 2.1.1 Aβ accumulation

A*β* pathology originates from the aberrant cleavage of amyloid precursor protein (APP) by *β*-secretase (*BACE1*) and *γ*-secretase, resulting in the production of aggregation-prone peptides, especially A*β*_42_^10^^,^^11^. In AD, impaired clearance mechanisms lead to both extracellular and intracellular accumulation of A*β*^12^^,^^13^. Soluble A*β* oligomers disrupt synaptic plasticity, activate microglia, and exacerbate oxidative stress and inflammation^14^^,^^15^. Furthermore, A*β* accumulation has been associated with tau hyperphosphorylation^16^. Genetic risk factors, notably the apolipoprotein E (*APOE*) *ε*4 allele, significantly influence A*β* aggregation and clearance dynamics^10^^,^^17^. AI techniques, such as unsupervised clustering and regression-based feature selection, have been extensively applied to associate A*β* burden with cognitive phenotypes and to identify molecular signatures predictive of amyloid accumulation across multi-omics datasets^18^^,^^19^. Therapeutic strategies targeting A*β* include *BACE1* inhibitors, *γ*-secretase modulators, and A*β* immunotherapies, though their clinical efficacy remains under active investigation^20^.

#### 2.1.2 Tau hyperphosphorylation and aggregation

Tau is a microtubule-associated protein essential for axonal transport^21^^,^^22^. In AD, kinases such as glycogen synthase kinase-3*β* (GSK-3*β*) and cyclin-dependent kinase 5 (CDK5) induce tau hyperphosphorylation, causing its dissociation from microtubules and subsequent aggregation into NFTs^12^. These aggregates disrupt the neuronal cytoskeleton and impair axonal integrity and function^15^^,^^23^. Increasing evidence indicates that A*β* pathology may further promote tau phosphorylation^21^^,^^24^. Advanced AI techniques, including deep learning and graph-based network inference, have been applied to phosphoproteomic and transcriptomic datasets to characterize kinase activity profiles and predict tau-related disease stages with high precision^25^, ^26^, ^27^. As a result, tau-targeting therapies, such as kinase inhibitors and tau immunotherapies, are under development to reduce neurotoxicity and slow cognitive decline^28^.

#### 2.1.3 Neuroinflammation

Neuroinflammation is increasingly recognized as a central contributor to the pathogenesis of AD. The accumulation of A*β* and hyperphosphorylated tau activates microglia and astrocytes, triggering an innate immune response^29^^,^^30^. While acute inflammation may aid in clearing toxic aggregates, chronic glial activation results in sustained release of proinflammatory cytokines and reactive oxygen species (ROS), which exacerbate synaptic dysfunction and neuronal injury^31^^,^^32^. Immune-related genes, such as triggering receptor expressed on myeloid cells 2 (*TREM2*), further influence microglial responses and disease progression^33^. AI-driven approaches, including network analysis and integrative omics, have been employed to identify immune-related molecular signatures and prioritize inflammatory mediators implicated in AD^34^^,^^35^. Therapeutic strategies targeting neuroinflammation include anti-inflammatory agents, inhibitors of cytokine signaling pathway, and modulators of microglial activity^36^, ^37^, ^38^.

#### 2.1.4 Synaptic dysfunction

Synaptic dysfunction is recognized as an early and critical event in the progression of AD^39^. A*β* oligomers disrupt synaptic signaling by interfering with neurotransmitter receptors, particularly *N*-methyl-d-aspartate (NMDA)-type glutamate receptors, leading to impaired long-term potentiation, calcium dysregulation, and oxidative stress^40^. Tau pathology further exacerbates synaptic damage by destabilizing microtubules and impairing axonal transport^22^. Recent AI-driven analyses of transcriptomic and proteomic datasets have facilitated the identification of key synaptic genes and network disruptions associated with early cognitive decline^41^. Therapeutic strategies aimed at mitigating A*β* and tau toxicity, including immunotherapies and receptor modulators, are currently under investigation for their potential to preserve synaptic integrity and slow cognitive deterioration^42^.

#### 2.1.5 Mitochondrial dysfunction

Mitochondrial dysfunction is a major contributor to the pathogenesis of AD, resulting in cellular energy deficits, elevated oxidative stress, and progressive neuronal loss^43^^,^^44^. Under physiological conditions, mitochondria supply adenosine triphosphate (ATP) essential for synaptic activity and neuronal survival. In AD, however, mitochondrial abnormalities, impaired oxidative phosphorylation, and excessive production of ROS are commonly observed^45^. A*β* and hyperphosphorylated tau can accumulate within mitochondria, disrupting the electron transport chain and exacerbating oxidative damage^46^. This bioenergetic failure compromises neuronal resilience and initiates a pathological feedback loop involving mitochondrial DNA damage, inflammation, and synaptic dysfunction^12^. AI-driven approaches have enabled more comprehensive analyses of mitochondrial gene expression profiles and metabolic pathway alterations^47^^,^^48^. Therapeutic strategies targeting mitochondrial dysfunction, such as antioxidants, mitochondrial biogenesis enhancers, and electron transport chain modulators, are being actively explored to interrupt this degenerative cycle.

#### 2.1.6 Autophagy and the UPS dysfunction

Proteostasis disruption is a hallmark of AD, contributing to the pathological accumulation of A*β* and hyperphosphorylated tau. These aggregation-prone proteins are normally degraded by two complementary systems: autophagy and the ubiquitin–proteasome system (UPS)^49^^,^^50^. Autophagy degrades misfolded proteins and damaged organelles *via* the lysosomal pathway, whereas the UPS targets aberrant proteins for proteasomal degradation^51^. In AD, both systems are impaired. Autophagic flux is often disrupted, leading to vacuolar accumulation, while reduced proteasomal activity diminishes protein clearance efficiency^12^^,^^51^. These deficits exacerbate synaptic dysfunction and neuronal toxicity. To investigate these proteostatic disruptions, AI-based methods such as quantitative modeling and integrative network analysis have been applied to assess autophagy and UPS-related pathways in AD models^52^^,^^53^. These computational approaches help uncover regulatory failures and identify key molecular players involved in disease progression. Building on these insights, several therapeutic strategies aim to enhance autophagy or UPS function^54^. Ongoing investigations are exploring the potential of autophagy inducers and proteasome enhancers to reduce protein aggregation and mitigate disease progression^38^.

#### 2.1.7 BDNF dysregulation

Brain-derived neurotrophic factor (BDNF) plays a critical role in supporting neuronal survival, synaptic plasticity, and memory formation^55^. In AD, reduced levels of BDNF and impaired signaling through its receptor, tropomyosin-related kinase B (TrkB), are particularly evident in the hippocampus and are strongly associated with cognitive decline^56^. This neurotrophic deficit impairs long-term potentiation and weakens synaptic function^57^. Accumulation of A*β* and hyperphosphorylated tau further disrupts the BDNF–TrkB signaling axis, exacerbating synaptic vulnerability^56^. To investigate these alterations, machine learning techniques have been employed to integrate transcriptomic and proteomic datasets, revealing widespread dysregulation within neurotrophic signaling networks in AD^41^^,^^58^. Therapeutic efforts are currently focused on restoring BDNF expression and enhancing TrkB receptor activity as potential strategies to preserve synaptic integrity and improve cognitive outcomes.

#### 2.1.8 Glutamate excitotoxicity

Glutamate excitotoxicity is a critical pathological mechanism in AD, driven by excessive activation of NMDA receptors. This overactivation leads to intracellular calcium overload, oxidative stress, and ultimately, neuronal death^59^. A*β* oligomers exacerbate excitotoxicity by impairing glutamate uptake and dysregulating receptor activity, thereby amplifying neuronal damage^60^. The resulting synaptic loss is closely associated with cognitive decline in affected individuals^59^. Recent advances in AI-powered computational modeling have enabled the simulation of excitotoxic signaling cascades and the prediction of responses to neuroprotective compounds^61^^,^^62^. Among current therapeutic options, memantine, an NMDA receptor antagonist, has demonstrated neuroprotective effects while preserving physiological glutamatergic transmission. Ongoing research is focused on optimizing such strategies to enhance efficacy and minimize side effects^60^.

#### 2.1.9 Gut–brain axis imbalance

Emerging evidence underscores a bidirectional relationship between the gut microbiota and the central nervous system, referred to as the gut–brain axis, which plays a significant role in the pathophysiology of AD^63^^,^^64^. In AD, gut dysbiosis has been associated with systemic inflammation, impaired immune homeostasis, and increased permeability of both the intestinal barrier and blood–brain barrier (BBB). These alterations collectively promote neuroinflammation, A*β* accumulation, and neuronal degeneration^64^^,^^65^. Furthermore, microbial metabolites, including neurotransmitters and short-chain fatty acids (SCFAs), influence synaptic function and cognitive performance^63^. AI-assisted multi-omics integration has been increasingly applied to investigate how microbiome alterations affect host metabolism and immune signaling in AD^66^, ^67^, ^68^. Therapeutic strategies aimed at restoring gut microbial balance through dietary interventions, probiotics, or prebiotics are currently under active investigation as potential approaches to modulate the gut–brain axis and attenuate disease progression.

### 2.2 Established biomarkers

**Figure 2:**
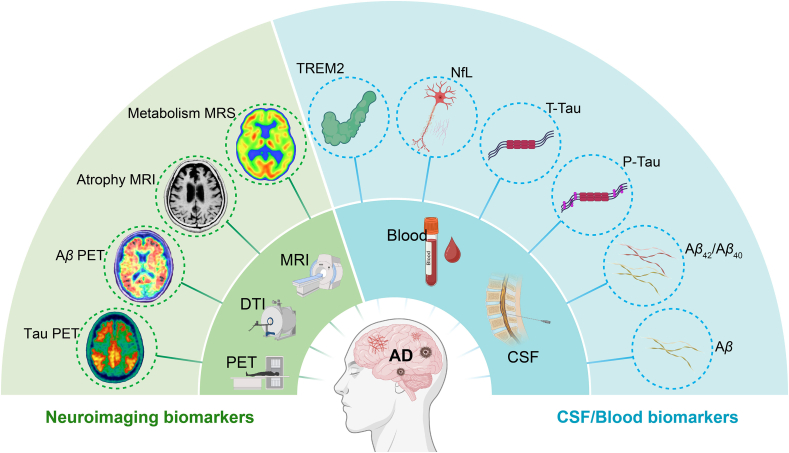
Neuroimaging and CSF/blood biomarkers associated with AD. On the left, neuroimaging biomarkers, including A*β* Positron emission tomography (PET), Tau PET, Atrophy magnetic resonance imaging (MRI), Metabolism magnetic resonance spectroscopy (MRS), and diffusion tensor imaging (DTI), show related brain changes in AD patients. On the right, cerebrospinal fluid (CSF)/blood biomarkers, such as triggering receptor expressed on myeloid cells 2 (*TREM2*), neurofilament light (NfL), total tau (t-tau), phosphorylated tau (p-tau), and the A*β*_42_/A*β*_40_ ratio, highlight biological changes in blood and CSF associated with AD.

The identification of reliable biomarkers is essential for enhancing the diagnosis, prognosis, and therapeutic monitoring of AD^69^. Current approaches, including cerebrospinal fluid (CSF) assays, neuroimaging modalities, and emerging blood-based markers, offer complementary perspectives on disease stage, underlying pathology, and progression dynamics (Fig. 2)^70^^,^^71^. To manage the complexity of these multimodal biomarker datasets, AI-powered computational tools are increasingly employed to perform data integration, pattern recognition, and predictive modeling. These techniques facilitate the development of precision medicine strategies that are tailored to the specific disease trajectories of individuals with AD.

#### 2.2.1 CSF biomarkers

CSF biomarkers provide direct biochemical evidence of neuropathological changes associated with AD^72^. The most well-established markers include A*β*_42_, total tau (t-tau), and phosphorylated tau (p-tau). A characteristic biomarker profile, defined by reduced levels of A*β*_42_ in conjunction with elevated t-tau and p-tau, provides a robust biochemical signature for detecting AD pathology and distinguishing it from other neurodegenerative disorders^72^^,^^73^. Recent studies have expanded the CSF biomarker panel to include neurofilament light (NfL), which reflects neuroaxonal damage, and soluble triggering receptor expressed on myeloid cells 2 (*sTREM2*), which is indicative of microglial activation^74^^,^^75^. These additional markers complement the core profile by providing insights into distinct aspects of disease pathophysiology. To enhance diagnostic accuracy, advanced AI techniques, such as machine learning classifiers and dimensionality reduction algorithms, have been applied to integrate multi-analyte CSF profiles with clinical metadata^76^, ^77^, ^78^, ^79^. Incorporating these biomarkers into diagnostic pipelines can improve early detection, facilitate patient stratification in clinical trials, and inform the development of personalized therapeutic strategies.

#### 2.2.2 Neuroimaging biomarkers

Neuroimaging biomarkers play a pivotal role in the diagnosis, staging, and therapeutic monitoring of AD by providing non-invasive, *in vivo* assessments of brain structure and function^80^^,^^81^. Structural magnetic resonance imaging (sMRI) is commonly used to detect atrophy in the hippocampus and medial temporal lobe, which are regions strongly associated with early cognitive decline in AD^80^. Functional MRI (fMRI) and resting-state connectivity analyses reveal alterations in neural networks involved in memory and executive functions^82^. Positron emission tomography (PET), utilizing tracers such as [^11^C] Pittsburgh compound B (PiB) and [^18^F]-labeled agents, enables the early detection of amyloid deposition. Tau PET imaging with ligands like [^18^F]flortaucipir allows for topographical mapping of tau pathology, which has been shown to correlate strongly with clinical severity^80^^,^^81^. AI-based neuroimaging analysis, particularly through the application of deep learning and pattern recognition techniques, has significantly advanced the interpretation and quantification of complex imaging data^83^^,^^84^. These methods support automated detection of subtle brain changes, enable disease trajectory prediction, and assist in patient selection for clinical trials. Additional modalities, including diffusion tensor imaging (DTI) and magnetic resonance spectroscopy (MRS), offer complementary insights into white matter integrity and metabolic alterations, further enhancing our ability to monitor disease progression^85^^,^^86^. Integrating these neuroimaging biomarkers with AI-driven analytical frameworks holds considerable promise for improving early detection, refining patient stratification in clinical studies, and informing the development of personalized treatment strategies.

#### 2.2.3 Blood-based biomarkers

Blood-based biomarkers are emerging as practical and scalable tools for early detection and longitudinal monitoring of AD^72^. In contrast to CSF and neuroimaging biomarkers, blood-based assays offer a minimally invasive and cost-effective alternative, making them well-suited for large-scale population screening and repeated assessments over time. Among the most promising indicators are plasma phosphorylated tau isoforms, including p-tau181 and p-tau217. These biomarkers exhibit high specificity in distinguishing AD from other dementias and show strong correlations with tau PET imaging and CSF tau concentrations^87^. In addition, plasma A*β*_42_/A*β*_40_ ratios have demonstrated potential for reflecting cerebral amyloid burden^88^, while NfL, a marker of axonal damage, is elevated in AD and provides prognostic insight into ongoing neurodegeneration^89^. AI-enabled platforms for biomarker discovery, which integrate large-scale cohort data with machine learning algorithms, have facilitated the identification, validation, and clinical implementation of blood-based biomarkers^90^^,^^91^. These approaches enhance the predictive accuracy and translational relevance of biomarker profiles. As validation studies expand to include more diverse and preclinical populations, the implementation of AI-driven blood biomarker panels is expected to improve early diagnosis, support precise patient stratification for clinical trials, and increase access to personalized interventions in routine care setting^72^.

## 3 Multi-omics in AD research

**Figure 3:**
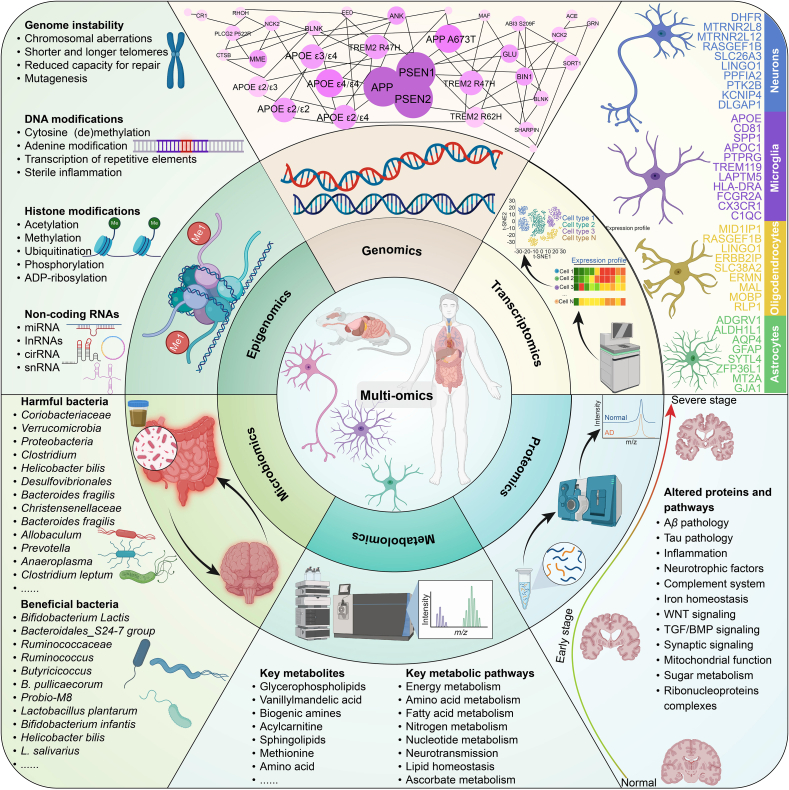
A comprehensive multi-omics framework for understanding AD through various molecular layers. It integrates genomics, epigenomics, transcriptomics, proteomics, metabolomics, and microbiomics to reveal key insights into AD pathology. The top left section shows genomic instability, highlighting chromosomal alterations, DNA modifications, and mutations related to AD genes, such as *APP*, presenilin 1 (*PS1*), and apolipoprotein E (*APOE*). The epigenomic panel outlines histone modifications and non-coding RNAs that contribute to disease progression. The transcriptomics section focuses on the expression of key genes in neurons, microglia, astrocytes, and oligodendrocytes, correlating with AD severity. The proteomics panel examines altered proteins and pathways involved in A*β* and tau pathology, inflammation, and neurodegeneration. Metabolomics identifies key metabolites and metabolic pathways, including those involved in energy and amino acid metabolism, as well as lipid and nitrogen metabolism. Lastly, the microbiomics section explores the roles of harmful and beneficial bacteria in the gut–brain axis.

Recent advances in high-throughput technologies have enabled systematic investigation of AD across multiple biological layers. These multi-omics approaches, including genomics, transcriptomics, proteomics, metabolomics, and microbiomics, reveal disease-associated alterations that deepen our understanding of AD's complex pathophysiology. Each omics layer captures a unique yet complementary facet of disease biology, from genetic susceptibility and gene expression to protein dynamics, metabolic shifts, and gut microbiome perturbations (Fig. 3). In this review, we summarize key discoveries and methodological advances within each omics domain that have enhanced our understanding of AD mechanisms (Table 1^92^, ^93^, ^94^, ^95^, ^96^, ^97^, ^98^, ^99^, ^100^, ^101^, ^102^, ^103^, ^104^, ^105^, ^106^, ^107^, ^108^, ^109^, ^110^, ^111^, ^112^, ^113^, ^114^, ^115^, ^116^, ^117^, ^118^, ^119^, ^120^, ^121^, ^122^, ^123^, ^124^, ^125^, ^126^, ^127^, ^128^, ^129^, ^130^, ^131^, ^132^, ^133^, ^134^, ^135^, ^136^, ^137^, ^138^, ^139^, ^140^, ^141^, ^142^, ^143^, ^144^, ^145^, ^146^, ^147^, ^148^, ^149^).

**Table 1:** Multi-omics used in the AD research.

Proteins/biomarkers	Omics	Biological sample	Integrative method	Main findings	Ref.
rs4802200: disrupts *E2F7* regulation of *KCNN4* (AD LTL module); rs117863556: interrupts *REST* regulation of *GAB2* in DLPFC	Genomics, transcriptomics, and epigenomics	Human brains	Yes	Non-coding SNPs are linked to AD-related gene expression, with 51 potential early neuroinflammatory biomarkers identified	92
*ANK1*, *CDH23*, *DIP2A*, *RHBDF2*, *RPL13*, *RNF34*, *SERPINF1*, and *SERPINF2*	Genomics, transcriptomics, and epigenomics	Human brains	Yes	Methylation at 71 of 415,848 CpGs links to AD pathology; eleven regions are validated; genes' expression is altered. This may start AD, which is seen in presymptomatic stages and *via* gene network	93
*SORL1*, *ABCA7*, *HLA-DRB5*, *SLC24A4*, and *BIN1*	Genomics, transcriptomics, and epigenomics	Human brains	Yes	Brain DNA methylation at AD loci ties to pathologies/hallmarks, gene expression correlates too, hinting at its AD role	94
*P2RX5*, *TRPV3*, *DPY30*, and *MEMO1*	Genomics, transcriptomics, and epigenomics	Human brains	Yes	Five loci linked to cell-type composition, with relevant genes, suggest cell-type shifts in neuropsychiatric disorder pathophysiology	95
Blood DNAm, CSF A*β*_42_, p-tau181, and t-tau	Genomics, transcriptomics, and epigenomics	Human blood and CSF	Yes	Connects blood DNAm to CSF AD biomarkers, finds AD treatments, tags *HOXA5* DNAm as biomarker, offers AD research resource	96
Tau and H3K9ac	Epigenomics, transcriptomics, and chromatin accessibility	Human brains, Mouse models, iNs	Yes	Tau-driven chromatin changes in AD surpass amyloid effects, involve the nuclear lamina, and can be studied and targeted using iNs and 17-DMAG	97
*SPI1*, *ELF2*, and *RUNX1*	Genomics, transcriptomics, and epigenomics	Human brains	Yes	Single-nucleus RNA and ATAC profiling revealed AD-associated epigenomic erosion, cell identity loss, and microglial regulatory risk loci	98
*ELOVL*, *FADS6*, *FAR1*, *FAR2*, *FABP7*, and *FA2H*	Epigenomics and lipidomics	Human plasma	Yes	Fatty acid metabolism linked to early AD, revealing diagnostic biomarkers and therapeutic targets	99
DMPs	Epigenomics	Human peripheral blood	Yes	DMPs linked to brain pathways and cognitive decline; validated as potential non-invasive AD biomarkers	100
*ANK1*, *MYOC*, *RHBDF2*, *GPR56*, *APOE*, and *IGF1*	Transcriptomics and epigenomics	Human brains	Yes	5246 DMPs are linked to AD severity (Braak stage, plaque density) and key pathways like IGF1 regulation	101
*APOE4*	Transcriptomics, proteomics, and phosphoproteomics	Mouse brains	Yes	BBB breakdown precedes synaptic and behavioral deficits; early EC dysfunction is key	102
SORLA	Transcriptomics	Human brains, iPSC-derived microglia	Yes	*SORL1* loss impairs *CD14* sorting, weakens pro-inflammatory signaling, and alters vesicle trafficking	103
H3K27ac and H3K9ac	Transcriptomics, proteomics, and epigenomics	Human brains	Yes	H3K27ac/H3K9ac enhancement disrupts chromatin feedback, worsens A*β* neurodegeneration	104
APOE	Transcriptomics and lipidomics	Human brains	Yes	APOE *ε2* boosts autophagy and ER stability; APOE *ε4* disrupts lipid and protein homeostasis	105
*SORL1*, *CHI3L1*, *HLA-E*, and *NEFL*	Transcriptomics	Human brains	Yes	Cell-specific transcriptomic alterations, reduced myelination, astrocytic stress signatures, and microglial activation highlight AD cellular dysfunction	106
*ITM2B*, *APOE*, *BIN1*, and *SPI1*	Transcriptomics and epigenomics	Human brains	Yes	Cross-disorder single-cell analyses identify shared pathways and disease-specific cellular states in tau-related neurodegenerative disorders	107
A*β*, pTau, NFTs, and APOE	Transcriptomics	Human brains	Yes	Amyloid plaques trigger microglial activation and inflammation, with APOE *ε*4 intensifying these effects	108
DAMs and DAAs	Transcriptomics	Mouse brains	Yes	Plaque-associated signaling impairs synapses, boosting GABAergic and reducing glutamatergic activity	109
miRNA-9, miRNA-125b, miRNA-146a, and miRNA-155	Transcriptomics	Human brains	Yes	Pro-inflammatory miRNAs are enriched in AD CSF/ECF, induced by AD ECF in cells, and suppressed by NF-*к*B inhibitors (CAPE, CAY10512)	110
miR-133b and NPTX2	Transcriptomics and proteomics	Human brains	Yes	miR-133b regulates NPTX2, influencing AD progression and cognition; mediation analysis confirms its neuroprotective role	111
miRNA-92a-3p, miRNA-486-5p, and miRNA-29a-3p	Transcriptomics	Plasma	Yes	Three miRNAs are identified as early AD biomarkers, linked to neuronal and structural pathways	112
DSBs	Genomics, transcriptomics, and epigenomics	Mouse and human brains	Yes	Neuron subtypes link to AD pathology; the cohesin complex and DNA damage genes are elevated in AD	113
PIGs and OLIGs	Transcriptomics	Mouse and human brains	Yes	Spatially dysregulated genes are identified as linked to metabolism, stress, and synaptic functions	114
*APP* K670N/M671L, *MAPT* P301L, and *PS1* M146V	Genomics, transcriptomics, and epigenomics	Human brains	Yes	Studies identify 124 spatially dysregulated genes; pathways are altered in lipid, mitochondrial stress-response and ion transport; BOK modulation shows potential	115
*APOE*, *CALM1*, *CALM3*, *LRP1*, and *SORL1*	Transcriptomics and genomics	Human brains	Yes	Studies identify 190 dysregulated ligand-receptor pairs; pathways are altered in amyloid and ion transport; EGFR inhibitors show potential	116
miR-132/212 and miR-184	Transcriptomics, metabolomics	Human brains.	Yes	2064 LOAD-specific genes, along with reduced RNA editing (*e*.*g*., GRIA2 Q/R) and miR-184-NR4A2 regulation, link to synaptic and mitochondrial dysfunction	117
Synaptic dysfunction, glial inflammation, myelination disruption, and sex-specific differences.	Transcriptomics	Human brains	Yes	AD involves synaptic dysfunction, glial inflammation, disrupted myelination, and sex-specific transcriptional differences, revealed by transcriptomics	118
CpG-rich *FMR1*	Epigenomics	Mouse NIH-3T3 and human cell lines	No	dCas9 allows precise demethylation, showing that selective promoter methylation impacts gene expression. This tool clarifies methylation's regulatory role	119
Tumor suppressor-mediated differentiation and involution	Transcriptomics	Human brains	No	TS pathways and differentiation drive early pathology, highlighting therapeutic targets	120
DAM, LRM, ARM, ERM, IRM, and TRM	Transcriptomics	Mouse and human brains	No	PAM drives inflammation; PCM aids plaque initiation. Both show distinct transcriptomic changes in AD	121
APs and NFTs	Transcriptomics	Human brains	Yes	The temporal lobe shows early abnormalities; cell-specific modules reveal therapeutic targets	122
DNA methylation	Epigenomics	Human brains	Yes	236, 95, and 10 CpGs are identified in cortex regions; none are found in cerebellum. Novel epigenetic loci are discovered	123
*APOE*, H3K9Ac	Genomics, transcriptomics, and epigenomics	Human brains	Yes	Multi-omics reveal key pathways in aging and AD	124
*APP*, *PS1* or *PS2*	Transcriptomics	Human brains	Yes	Synaptic and amyloid-related genes change before plaques and tangles	125
*PIK3R1*, *BTN3A3*, *NHLH1*, and *SLC16A7*	Transcriptomics and epigenomics	Human brains	Yes	DNA methylation-gene expression correlations are identified, highlighting potential pathogenic mechanisms in psychotic disorders	126
*CLU*, *SYNJ2*, *NCOR2*, *RAI1*, *CXXC5*, *INPP5A*, *MCF2L*, *ANK1*, *MAP2*, *LRRC8B*, *STK32C*, *S100B*, *HOXA3*, *APP*, and *ADAM17*	Epigenomics	Human brains	Yes	Aging- and Braak stage-related epigenetic markers like *APP* and *HOXA3* are identified	127
*IQCK*, *ACE*, *ADAM10*, *ADAMTS1*, and *WWOX*	Genomics	Human blood and brains	Yes	Five new loci are identified, linking APP, tau, and immune pathways	128
*APP*, *PS1*, and *PS2*	Genomics	Human blood and brains	Yes	29 loci are identified, linking key pathways to AD risk	129
*TYROBP* (aka DAP12)	Genomics and transcriptomics	Human brains	Yes	*TYROBP* regulates immune/microglial networks central to LOAD	130
*APOE* and *ABCA7*	Genomics	Human blood	Yes	Two novel loci (COBL, SLC10A2) linked to AD in African Americans	131
Ng	Proteomics	Human CSF	No	Elevated CSF Ng predicts synaptic loss and cognitive decline in AD	132
Docosahexaenoic acid (C22:6n-3) and tetracosahexaenoic acid (C24:6n-3)	Lipidomics	Human liver and brains	No	Reduced DHA and enzyme activity in AD correlate with cognitive impairment	133
GSK-3*β*, CDK5, PKC, MAPK, ROCK2, and PP2A	Proteomics and phosphoproteomics	Human brains	Yes	Protein and phosphosite changes are identified as linked to AD stages	134
LXRs, SREBPs, IDOL, HMGCR, and CYP46A1	Lipidomics	Mouse brains and plasma	Yes	Probiotics improve lipid homeostasis and reduce AD pathology	135
*CR1*, *PLCG2*, *MEF2C*, *IL34*, *ABCA7*, *PTK2B*, *HLA-DRB1*, *MS4A6A*, and *APOE ε4*	Lipidomics and proteomics.	Human plasma	Yes	Lipid and protein modules linked to AD highlight these pathways	136
Metabolic dysregulation, oxidative stress, neuroinflammation, complement activation, synaptic dysfunction, and vitamin B2/B5/B6 pathway abnormalities	Transcriptomics, proteomics, and metabolomics	Human brains, blood, and CSF; mouse brains, blood, and CSF	Yes	Deregulated pathways, particularly those involving neurotransmitter synapses, inflammation, oxidative stress, and the metabolism of vitamins B2, B5, and B6, are associated with the progression of AD and may represent potential therapeutic targets	137
PC, LPC, PE, phosphatidylinositol, SM, ceramide, and TAG	Lipidomics	Human plasma	No	Reduced sphingomyelin and elevated ceramides correlate with AD severity	138
A*β*_42_	Proteomics	Mouse and human brains	No	Met-35 is key to A*β*_42_ toxicity; altering it reduces damage	139
*APP*SWE/*PS1*ΔE9	Transcriptomics and metagenomics	Mouse brains	Yes	Microbiome alterations reduce A*β* and restore microglial homeostasis in male mice only	140
SCFAs, BDNF, NGF-A1, ZO-1, Occludin, and Claudin-5	Transcriptomics and metagenomics	Mouse brains and gut samples	Yes	Microbiota or metabolites restore BBB integrity in germ-free mice	141
FGG	Proteomics	Human plasma	No	Plasma FGG and age predict amyloid burden with moderate accuracy	142
MDK, NTN1, SMOC1, SLIT2 and HTRA1	Proteomics, phosphoproteomics, and transcriptomics	Human brains and CSF, mouse brains	Yes	Key proteins/pathways are linked to AD progression, with distinct human-mouse differences in autophagy and inflammation	143
BIN1, PICALM, PTK2B, and FERMT2	Proteomics, proteogenomics and transcriptomics	Human brains	Yes	RNA-binding proteins and splicing events are identified linked to pathology and cognitive decline	144
*TREM2*	Transcriptomics and proteomics	Human brains	Yes	Altered microglial activation is observed; there are no significant A*β* or tau differences	145
t-tau, SNCA, JPH3, CFP, and PI15	Proteomics, metabolomics, and lipidomics	Human plasma.	Yes	Oleamide and several proteins are identified as key molecules distinguishing MCI subtypes and AD	146
H3 K18/K23	Epigenomics	Human brains	No	H3 K18/K23 acetylation is significantly lower in AD temporal lobe *vs.* controls	147
RSS	Proteomics	Mouse brains	No	TPsC and protein polysulfides decrease in 5xFAD mice	148
A*β*_42_, t-tau, p-tau181, ^11^C-PET imaging, ^18^F-PET imaging, MRI (especially hippocampal volume-related content)	Proteomics	Human plasma	No	Plasma APOE, BNP, CRP, and pancreatic polypeptide correlate with CSF amyloid and tau	149

Note: AD, Alzheimer's disease; *APP*, amyloid precursor protein; ARM, activated-response microglia; BBB, blood-brain barrier; *BIN1*, bridging integrator 1; CFP, cerulean fluorescent protein; CSF, cerebrospinal fluid; DAAs, disease-associated astrocytes; DAMs, disease-associated microglia; DLPFC, dorsolateral prefrontal cortex; DMPs, differentially methylated positions; DNAm, DNA methylation; DSBs, DNA double-strand breaks; EC, endothelial cell; ERM, early responsive microglia; FERMT2, fermitin family member 2; HTRA1, HtrA serine peptidase 1; iNs, induced neurons; IRM, interferon-response microglia; JPH3, junctophilin 3; LPC, lysophosphatidylcholine; LRM, late response microglia; MRI, magnetic resonance imaging; NAB, neocortical amyloid burden; NFTs, neurofibrillary tangles; NTN1, netrin-1; PAM, plaque-associated microglia; PC, phosphatidylcholine; PE, ethanolamine glycerophospholipid; PI15, protein inhibitor 15; PICALM, phosphatidylinositol binding clathrin assembly; PTK2B, protein tyrosine kinase 2 beta; PS1, presenilin 1; RSS, reactive sulfur species; SLIT2, slit homolog 2; SM, sphingomyelin; SMOC1, SPARC-related modular calcium-binding 1; SNCA, alpha-synuclein; TAG, triacylglycerol; TPsC, total polysulfide content; *TREM2*, triggering receptor expressed on myeloid cells 2; TRM, transiting response microglia; t-tau, total tau; MCI, mild cognitive impairment; DHA, docosahexaenoic acid; ANK1, ankyrin 1; CLU, clusterin; LTL, Lateral Temporal Lobe; PET, positron emission tomography; p-tau, phosphorylated tau; PIG, plaque-induced gene; OLIG, oligodendrocyte gene; SCFAs, short-chain fatty acids; ATAC, assay for transposase-accessible chromatin; BDNF, Brain-derived neurotrophic factor; CDK5, cyclin-dependent kinase 5; Ng, Neurogranin; GSK-3*β*, glycogen synthase kinase-3*β;* PCM, plaque-distant microglia.

### 3.1 Genomics and epigenomics

AD is increasingly recognized as a heterogeneous disorder influenced by both genetic and environmental factors. Over the last two decades, genome-wide association studies (GWAS) have identified more than 40 risk loci associated with pathways including lipid metabolism, immune regulation, and endosomal trafficking^128^^,^^129^^,^^150^^,^^151^. The *APOE* gene remains the prominent genetic risk factor, with the *ε*4 allele significantly increasing susceptibility. In addition, recently identified variants in genes such as bridging integrator 1 (*BIN1*) and clusterin (*CLU*) highlight the multifactorial nature of the disease and extend the genetic landscape beyond the classical amyloid and tau pathways^128^^,^^150^. Many of these variants have modest individual effect sizes, indicating a polygenic model in which numerous low-to moderate-impact alleles collectively modulate disease risk^129^.

Genetic predisposition alone does not fully explain the observed variability in the onset and progression of AD. This limitation has driven growing interest in epigenomic regulation, which includes mechanisms such as DNA methylation, histone modifications, and chromatin remodeling. Early epigenome-wide association studies (EWAS) identified differentially methylated regions (DMRs) in genes including ankyrin 1 (*ANK1*) and *BIN1*, suggesting that epigenetic modifications may converge on biological pathways previously implicated by genetic studies^124^^,^^152^. Notably, methylation changes at these loci often correlate with altered gene expression and chromatin accessibility, indicating that epigenetic reprogramming may amplify or mitigate the functional impact of genetic variants^126^. Thus, the epigenome serves as a critical interface through which environmental influences, including inflammation, lifestyle factors, and metabolic stress, can modulate the course of AD, even in individuals with established genetic risk^153^.

The methodological landscape for AD research has become increasingly sophisticated. GWAS and whole-genome sequencing (WGS) provide comprehensive coverage of common and rare variants across the entire genome^128^. In contrast, targeted capture and whole-exome sequencing (WES) focus specifically on protein-coding regions, which may be more amenable to therapeutic targeting^131^. However, these genome-wide approaches do not directly elucidate how regulatory variants influence gene function. To address this gap, epigenomic profiling techniques, such as DNA methylation arrays, reduced representation bisulfite sequencing (RRBS), and chromatin immunoprecipitation followed by sequencing (ChIP-seq), offer critical insights into the regulatory elements that modulate gene expression and splicing^123^^,^^154^. Meanwhile, methods like assay for transposase-accessible chromatin using sequencing (ATAC-seq) and single-cell epigenomic technologies have advanced the ability to map chromatin accessibility and characterize three-dimensional chromatin architecture. These approaches also enable the identification of cell-type specific enhancer–promoter interactions in brain tissue^155^.

The growing volume and complexity of genomic and epigenomic data have accelerated the adoption of AI and machine learning techniques to identify disease-associated variants, predict regulatory elements, and integrate multi-omics datasets. These computational strategies enable more accurate mapping of risk loci, functional annotation of regulatory elements, and inference of gene regulatory networks from large-scale data resources. These advancements, several key challenges remain. A major limitation is the reliance on post-mortem brain tissue, which restricts the ability to perform longitudinal analyses and may underrepresent the dynamic nature of epigenetic changes throughout the progression of AD^156^. Furthermore, the cellular heterogeneity of the AD brain presents significant analytical hurdles. Advanced computational deconvolution techniques or experimental methods such as fluorescence-activated cell sorting (FACS) are often required to resolve distinct cell populations, including neurons, astrocytes, and microglia^127^^,^^157^. This level of resolution is essential, as epigenetic profiles in glial cells may differ substantially from those in neurons and may play critical roles in processes such as amyloid plaque deposition and neuroinflammation^118^. Additionally, establishing causal relationships remains a major challenge. The identification of an epigenetic signature does not necessarily confirm its mechanistic relevance. Functional validation using CRISPR-based genome and epigenome editing technologies is increasingly employed to test the biological significance of candidate regulatory loci^119^^,^^158^.

There is increasing recognition that integrating genomics, epigenomics, and transcriptomics within the same biological samples, a strategy commonly referred to as multi-omics integration, can yield highly informative insights into the molecular mechanisms of AD^159^^,^^160^. By analyzing how genetic variants intersect with epigenetic modifications and gene expression profiles, researchers can identify specific regulatory nodes where disease-associated alleles exert their influence. AI-driven integrative analyses offer particular promise in elucidating how variants in genes such as *APOE* and *BIN1* affect chromatin structure or methylation states within distinct neuronal populations. These approaches facilitate a systems-level view of gene regulation that is not achievable through single-layer analyses alone. A fully integrated analytical framework that captures the interplay among genetic variation, epigenetic regulation, and transcriptional activity is essential for advancing our understanding of AD. When supported by AI-based data synthesis, such a framework holds strong potential for discovering novel biomarkers and clarifying the molecular mechanisms underlying disease progression. It can also guide the development of personalized therapeutic strategies that are tailored to an individual's unique molecular profile.

### 3.2 Transcriptomics

Over the past decade, transcriptomics has become a cornerstone in elucidating the complex molecular landscape of AD. It provides key insights into the dynamic, cell-type-specific processes that underlie disease onset and progression. Early transcriptomic studies utilizing microarrays revealed alterations in synaptic, immune, and metabolic pathways that appear even before clinical symptoms, underscoring the potential for pre-symptomatic intervention^120^^,^^125^^,^^161^. However, microarrays are limited by their probe-based design and restricted dynamic range, which led to a transition toward high-throughput bulk RNA sequencing (RNA-Seq). RNA-Seq offers an unbiased view of known and novel transcripts and has identified candidate “hub” genes within networks linked to lipid metabolism and innate immunity^122^^,^^130^^,^^159^^,^^160^. Despite these advances, bulk RNA-Seq cannot resolve cell-specific transcriptional changes due to tissue heterogeneity^122^^,^^124^. Single-cell RNA sequencing (scRNA-Seq) addresses this limitation by enabling high-resolution profiling of individual cell types. This technique has revealed distinct microglial and astrocytic subpopulations, such as disease-associated microglia (DAM) and reactive astrocytes, which are involved in plaque clearance, neuroinflammation, and synaptic modulation^118^^,^^162^, ^163^, ^164^.

AI methods have become indispensable tools for processing, clustering, and interpreting these high-dimensional single-cell datasets. These methods have facilitated the discovery of subtle cellular subtypes and gene regulatory modules associated with disease progression. Recent advances in single-nucleus RNA sequencing (snRNA-Seq) have extended transcriptomic analysis to frozen or archived post-mortem brain tissues. By isolating nuclei rather than whole cells, snRNA-Seq retains transcriptional fidelity while avoiding cell dissociation artifacts^155^^,^^165^. This technique has enabled detailed profiling of vulnerable neuronal subtypes and glial populations. However, data processing and integration remain computationally demanding, prompting the adoption of AI-based approaches for normalization, batch correction, and cell-type annotation.

Spatial transcriptomics further enhances transcriptomic profiling by preserving the spatial context of gene expression. It allows gene expression patterns to be mapped onto histologically defined regions, thereby linking molecular signatures to specific pathological features such as amyloid plaques and tau tangles^114^^,^^121^^,^^166^^,^^167^. When integrated with AI-enhanced image analysis, spatial transcriptomics provides more precise insights into the relationship between gene expression and tissue architecture. Each of these technologies offers distinct advantages and limitations. Microarrays and bulk RNA-Seq are scalable and cost-effective but lack single-cell resolution. Single-cell and single-nucleus methods deliver high granularity but require significant technical and computational resources. Spatial transcriptomics adds a critical spatial dimension but may trade off resolution or transcriptomic depth^114^^,^^118^^,^^162^^,^^166^.

Looking ahead, integrative multi-omics approaches are expected to play a pivotal role. Studies correlating transcriptomic profiles with proteomic, metabolomic, and epigenomic data have already revealed key discrepancies between RNA and protein expression in AD^89^^,^^159^^,^^160^. Moreover, combining single-cell or spatial transcriptomics with imaging modalities such as fluorescence in situ hybridization (FISH) or immunohistochemistry enhances validation within native tissue contexts^114^^,^^167^. Therefore, a systems biology framework that integrates these diverse molecular layers, supported by AI-powered analytics, will be crucial for illuminating the pathophysiological cascades of AD and accelerating the discovery of biomarkers and precision therapies.

### 3.3 Proteomics and metabolomics

Although genetic and transcriptomic studies have provided critical insights into AD, proteomics and metabolomics directly capture functional endpoints such as proteins and metabolites, which often act as early indicators or amplifiers of disease progression^156^^,^^159^. Utilizing these data types is essential for understanding the complex nature of AD pathogenesis and developing targeted interventions.

Contemporary proteomic research relies heavily on high-resolution mass spectrometry (HR-MS) platforms including Orbitrap and time-of-flight (TOF) systems. These tools quantify thousands of proteins across a wide dynamic range^168^^,^^169^. Techniques such as tandem mass tag (TMT) labeling, isobaric tagging, and label-free quantification allow for systematic comparisons of proteomes in brain tissue, CSF, and plasma. These approaches have revealed dysregulation in synaptic proteins, immune modulators, and metabolic enzymes that extend beyond the classical amyloid and tau pathways^134^^,^^160^^,^^170^, ^171^, ^172^, ^173^.

Proteins are subject to post-translational modifications (PTMs) including phosphorylation, ubiquitination, glycosylation, and oxidation, each of which can alter protein stability and function. Specific phosphorylation patterns on tau influence tangle formation, while ubiquitination may accelerate synaptic protein degradation and contribute to neurodegeneration^174^, ^175^, ^176^, ^177^. Advances in phosphoproteomics and glycoprotein enrichment technologies now enable proteome-wide mapping of these modifications. Given the complexity and low abundance of many PTMs, AI-based analytical methods are increasingly used for enrichment, site identification, and functional network modeling^139^^,^^169^^,^^178^^,^^179^. Most proteomic studies rely on bulk samples, but recent innovations in single-cell and spatial proteomics have enabled the identification of protein expression changes in specific cell types and anatomical regions. These methods allow researchers to examine the protein landscape surrounding plaques and within vulnerable brain areas such as the hippocampus^180^, ^181^, ^182^, ^183^. AI-enhanced image segmentation has improved the resolution and accuracy of these spatial patterns. However, single-cell proteomics still faces limitations such as lower proteome coverage and higher per-sample cost^184^^,^^185^.

Metabolomics complements proteomics by quantifying small molecules that reflect or influence cellular phenotypes. Untargeted methods like ultra-high-performance liquid chro-matography–mass spectrometry (UHPLC–MS) and nuclear magnetic resonance (NMR) capture broad metabolic profiles, while targeted approaches focus on specific pathways such as the tricarboxylic acid cycle and sphingolipid biosynthesis^138^^,^^186^, ^187^, ^188^, ^189^, ^190^, ^191^. These techniques have uncovered metabolic abnormalities in AD, particularly those related to lipid metabolism, mitochondrial dysfunction, and inflammatory signaling^192^^,^^193^. AI approaches are being used to analyze high-dimensional metabolomic datasets, identify diagnostic patterns, and enhance predictive power. Despite variability introduced by diet and comorbid conditions, metabolomic biomarkers such as reduced docosahexaenoic acid (DHA) and elevated homocysteine have demonstrated clinical utility^133^^,^^194^, ^195^, ^196^, ^197^, ^198^, ^199^. Metabolic shifts detectable in plasma or CSF may precede pathological changes in amyloid or tau, offering value for early diagnosis^200^. Single-cell and spatial metabolomics, including matrix-assisted laser desorption ionization imaging, now enable in situ mapping of metabolites around pathological structures. These methods provide localized biochemical context but currently face challenges in sensitivity and throughput^183^^,^^201^, ^202^, ^203^, ^204^.

Multi-omics integration that combines genomic, transcriptomic, proteomic, and metabolomic layers is creating more comprehensive models of AD biology^156^^,^^159^^,^^160^. Linking genetic variation to downstream protein networks and metabolic processes has led to the identification of novel therapeutic targets^137^^,^^160^^,^^205^. For example, individuals carrying the *APOE ε4* allele display distinct proteo-metabolomic signatures in lipid transport and immune pathways^206^^,^^207^. AI-driven integration and modeling are now essential tools for discovering biomarkers and stratifying patients. In clinical settings, proteomic and metabolomic biomarkers offer promising avenues for earlier detection, more precise monitoring, and tailored therapeutic response^186^^,^^208^^,^^209^. Several candidate biomarkers, such as neurogranin in CSF, are undergoing validation alongside amyloid and tau panels. Meanwhile, metabolomic profiles are being explored for guiding clinical trial inclusion and personalized intervention strategies^132^^,^^136^^,^^210^. Future directions should prioritize longitudinal and multi-modal studies that integrate omics, neuroimaging, and pathology to uncover the molecular mechanisms of AD and accelerate the development of individualized therapies^208^^,^^211^.

### 3.4 Microbiomics

While genomic, transcriptomic, and proteomic studies have deepened our understanding of AD, microbiomics has introduced a critical new dimension by elucidating the role of the gut–brain axis in neurodegeneration^65^^,^^195^. Studies using 16S rRNA gene sequencing and shotgun metagenomic sequencing have consistently identified significant changes in the gut microbiota composition of individuals with AD. These include reductions in beneficial genera such as *Lactobacillus* and *Bifidobacterium*, alongside increases in pro-inflammatory taxa like *Enterobacteriaceae*^64^. Reduced microbial diversity and taxonomic shifts have been associated with cognitive impairment, suggesting that microbial dysbiosis may contribute to AD *via* modulation of systemic and neuroinflammatory responses^212^.

Dysbiosis is hypothesized to affect AD through multiple pathways, including the production of SCFAs, regulation of immune function, and modulation of BBB integrity. Pro-inflammatory microbial taxa have been linked to elevated amyloid burden and systemic inflammation in cognitively impaired individuals^213^. In germ-free mouse models, increased BBB permeability has been observed, which can be reversed through colonization with SCFA-producing bacteria^141^. Shotgun metagenomic sequencing, analyzed using AI-enhanced bioinformatics, has revealed functional disruptions in AD-associated microbiomes, including altered lipid metabolism and reduced SCFA biosynthesis capacity^214^. Moreover, sex-specific differences have been noted, with female AD mouse models showing more pronounced microbiota-driven amyloid deposition and microglial activation compared to males^140^.

While 16S rRNA sequencing is efficient for profiling bacterial communities, it lacks species-level resolution and omits non-bacterial microbiota such as fungi and viruses^215^. Shotgun metagenomics provides more comprehensive functional insights but requires higher cost and computational power. AI algorithms are increasingly used to separate host from microbial DNA and to enhance functional annotation^216^^,^^217^. Furthermore, confounding factors such as diet, age, and antibiotic use complicate microbiome data interpretation^217^. Longitudinal studies are essential to establish causal relationships between microbiota shifts and AD progression^170^^,^^187^. Notably, antibiotic-induced depletion of gut microbiota in AD mouse models has been shown to reduce amyloid accumulation and modulate microglial activation^65^. Interventions targeting the gut microbiota, including probiotics, prebiotics, dietary modification, and fecal microbiota transplantation (FMT), are under active investigation. Clinical trials have shown that probiotic supplementation can improve cognitive function and reduce inflammation in AD patients^218^. Other strategies, such as SCFA supplementation and FMT, have demonstrated potential for restoring microbial balance and reducing neuroinflammation, although larger, well-controlled studies are needed to validate these effects^135^.

Integrating microbiomics with other omics layers, such as genomics, transcriptomics, and metabolomics, is crucial for a systems-level understanding of microbiome–host interactions in AD^133^^,^^219^. AI-driven multi-omics integration enables linking microbial genes with host immune responses and amyloid processing pathways^205^. Machine learning applied to metabolomic data can also identify how microbial metabolites affect neuronal health and inflammatory status^118^.

Emerging technologies such as single-cell microbiomics and spatial transcriptomics, combined with AI-based analytical platforms, will offer unprecedented spatial and cellular resolution for studying microbiome–brain interactions^220^^,^^221^. These tools can help identify specific microbial strains and their localized effects within the gut and central nervous system. To ensure reproducibility and translational relevance, standardized protocols and large longitudinal cohorts are needed^222^^,^^223^. Despite ongoing methodological challenges, the microbiome represents a promising frontier in AD research. Advances in microbiome-targeted diagnostics and therapeutics, particularly those integrated with multi-omics and AI approaches, may offer novel opportunities for early intervention and disease modification.

## 4 AI in multi-omics data integration and analysis of AD

**Figure 4:**
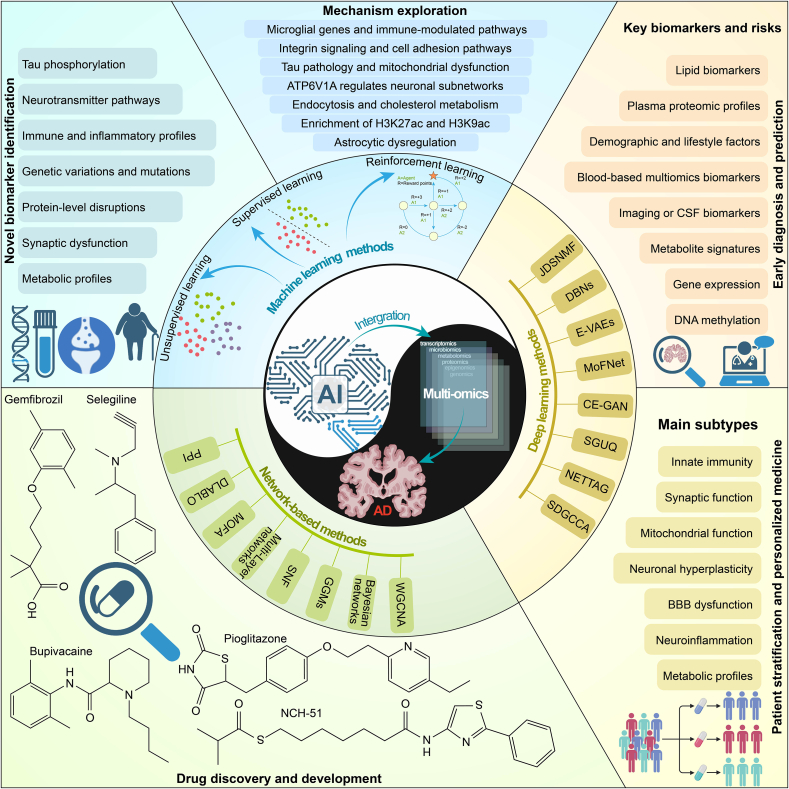
The application of Artificial Intelligence (AI) in integrating multi-omics data for AD research. Machine learning, deep learning, and network-based methods are essential AI techniques in multi-omics integration for AD research. Machine learning methods, such as supervised (SVM, Random Forests), unsupervised (K-means), and reinforcement learning, predict outcomes and uncover hidden patterns. Deep learning techniques like Deep Neural Networks (DNN), Convolutional Neural Networks (CNNs), and Variational Autoencoders (V-AEs) are used for complex data analysis, including brain image recognition and gene expression. Network-based methods, such as Weighted Gene Co-expression Network Analysis (WGCNA) and Bayesian networks, analyze gene interactions and protein pathways to identify key biomarkers and molecular mechanisms in AD. These multi-omics integrated by these AI integrative techniques are used for the identification of novel biomarkers, mechanism exploration, early diagnosis and prediction of disease, drug discovery and development, and patient stratification and personalized medicine.

Recent advances in multi-omics technologies have enabled simultaneous profiling of genomics, transcriptomics, proteomics, metabolomics, and epigenomics within AD cohorts. These platforms generate high-dimensional datasets that reflect the biological complexity of the disease across multiple molecular layers^159^. However, effective integration of such data remains a significant challenge due to differences in data scale, noise characteristics, missing value patterns, and the often weak biological signals relative to technical variation^224^. Successful integration requires a multistep preprocessing pipeline that includes batch effect correction, normalization, feature selection, and sample alignment. This is followed by statistical modeling approaches capable of capturing both shared and modality-specific variation across omics types^225^. Common methods include multiblock partial least squares, similarity network fusion, matrix factorization, and manifold learning techniques, each offering different strengths in capturing complex inter-omic relationships^226^. AI plays a central role in multi-omics data integration (Fig. 4). Classical machine learning algorithms such as random forests and support vector machines have demonstrated strong performance on curated datasets, providing interpretable feature rankings that aid in biomarker discovery^227^. More recent deep learning architectures, including variational autoencoders, graph neural networks, and transformer models, offer the capacity to handle large feature spaces and to model nonlinear, cross-omic interactions. In addition, network-based approaches that incorporate prior biological knowledge improve both model performance and interpretability^228^ (Table 2^34^^,^^41^^,^^53^^,^^225^^,^^227^, ^228^, ^229^, ^230^, ^231^, ^232^, ^233^, ^234^, ^235^, ^236^, ^237^, ^238^, ^239^, ^240^, ^241^, ^242^, ^243^, ^244^, ^245^, ^246^, ^247^, ^248^, ^249^, ^250^, ^251^, ^252^, ^253^, ^254^, ^255^, ^256^, ^257^, ^258^, ^259^, ^260^, ^261^, ^262^, ^263^, ^264^, ^265^, ^266^, ^267^, ^268^, ^269^, ^270^, ^271^, ^272^, ^273^, ^274^).

**Table 2:** The AI model used for the integration of various multi-omics of AD.

AI models	Key features	Representative applications	Advantages/Disadvantages	Main challenges	Evaluation metrics	Ref.
SVM	Serves as a supervised classifier for multi-omics integration	Applies to classify AD phenotypes and identify potential biomarkers in multi-omics datasets	Advantages: High accuracy in AD phenotype classification; well-established generalization capacity. Disadvantages: Requires vector reshaping of matrix data; sensitive to imbalanced inputs; high computational cost	Requires tuning for large datasets; limited interpretability in non-linear implementations		229
Linear SVM	Uses linear decision boundaries for classification	Performs binary classification tasks, such as differentiating AD from healthy subjects	Advantages: Simple formulation with fast computation; supports feature selection and outlier detection *via* MILP integration. Disadvantages: Limited to linear separability; reduced generalization for non-linear AD patterns	Requires kernel or feature mapping for non-linear patterns; tuning needed for optimal boundary definition		230
Non-linear SVM	Combines 51 AD SNPs and 8 environmental factors; uses SMOTE and grid-search tuning to handle class imbalance	AD classification on 184 cases and 3773 controls from Taiwan Biobank and CGMH; delivers 80% accuracy and specificity comparable to ANN and RF baselines	Advantages: Effective for AD classification using genetic and environmental features; comparable accuracy to ANN and RF. Disadvantages: High computational cost; sensitive to feature redundancy and data imbalance	Requires careful kernel and parameter tuning; vulnerable to noisy or heterogeneous omics data	Accuracy: 0.97; Sensitivity: 0.98; Specificity: 0.96; F1 Score: 0.97; MCC: 0.94; ROC-AUC: 0.99	231
RF	Serves as an ensemble method that uses multiple decision trees to improve accuracy and prevent overfitting	Predicts disease progression and classifies MCI as AD	Advantages: Handles high-dimensional AD data; provides built-in feature importance; robust to noise and overfitting. Disadvantages: High computational cost with large datasets; performance may degrade with redundant features	Limited interpretability due to large ensembles	AUC: 0.946; F1-score: 0.824; OOB Error: 0.1597	232
Decision trees	Create binary splits for classification (used as base learners in RF)	Serve as base learners or stand-alone interpretable models for stage classification	Advantages: Easy to interpret; effective in classifying AD stages using gene expression. Disadvantages: Easily overfits on small or high-dimensional AD data; lacks regularization mechanisms	Poor robustness in complex datasets; difficult to generalize without pruning or ensemble methods	Accuracy: 0.72; Sensitivity: 0.72; Specificity: 0.86; Precision: 0.75; F1 score: 0.72	233
Boosting methods	Combine multiple weak learners to form a strong model	Enhance feature selection and predict clinical outcomes based on multi-omics data	Advantages: Achieves high prediction accuracy on AD severity using multi-omics features; integrates diverse data types including proteomics, metabolomics, and microbiome. Disadvantages: Requires careful parameter tuning to prevent overfitting; performance may decline with noisy or imbalanced data	Sensitive to data noise and imbalance; necessitates meticulous tuning to maintain model generalization	AUC: 1.00 (training), 0.94 (validation), 0.87 (test); Accuracy: 0.973 (multi-omics, XGBoost): 0.973 (multi-omics, XGBoost)	234
M-LSTM	Captures long-term temporal dependencies in time-series data using memory-gated recurrent network	Detects AD progression using cognitive assessments and neuroimaging data	Advantages: High accuracy; models progression over time; robust to missing data. Disadvantages: Computationally intensive; requires careful preprocessing and tuning	Handling irregular visit intervals and missing values; optimizing hyperparameters for classification	Accuracy: 0.8178; Precision: 0.8550; Sensitivity: 0.8000; F1 score: 0.8266	235
AdaBoost	Combines multiple weak classifiers to improve prediction performance	Classifies AD subjects into progressive *vs* nonprogressive categories based on cognitive and imaging data	Advantages: High interpretability; improves accuracy *via* boosting. Disadvantages: Less effective for sequential data; limited in capturing temporal patterns	Enhancing accuracy with limited features; addressing class imbalance in progression labels	Accuracy: 0.7981; Precision: 0.7982; Sensitivity: 0.8125; F1 score: 0.8053	235
XGBoost–SHAP	Combines weighted XGBoost with SHAP for interpretable feature attribution	Multiclass classification of NC, MCI, and AD using ADNI and NACC datasets with clinical, imaging, and genetic data	Advantages: High classification accuracy with clinical interpretability; better performance than RF, Bagging, AdaBoost, and NB. Disadvantages: Requires hyperparameter tuning; sensitive to class imbalance	Weight adjustment of imbalanced classes; optimizing clinical utility across different data sources	ADNI: Accuracy: 0.88; Sensitivity: 0.81; Specificity: 0.92; AUC: 0.91; CUI+: 0.71; CUI–: 0.75 NACC: Accuracy: 0.81; Sensitivity: 0.75; Specificity: 0.90; AUC: 0.88; CUI+: 0.56; CUI–: 0.68	236
DBNs	Compose a probabilistic deep architecture from stacked RBMs optimized *via* Bayesian hyperparameter tuning	Classify AD using integrated gene expression and DNA methylation data	Advantages: High test accuracy with low feature count; handles HDLSS data effectively. Disadvantages: Requires extensive tuning; sensitive to omics integration noise	Optimizing architecture; addressing small sample bias in omics data	Accuracy (5-fold CV): 0.9956 (avg); Accuracy on test datasets: 0.82 (GSE109887/118553); Features selected: 39	237
GNNs	Operate on a signed, directed, and heterogeneous biomedical knowledge graph to learn drug combination embeddings	Support drug repurposing for AD based on expert-weighted multi-relational biomedical data	Advantages: Integrates expert guidance with biomedical networks; identifies clinically relevant 5-drug combinations. Disadvantages: Black-box structure limits interpretability; graph construction requires domain-specific effort	Designing interpretable graph structures; validating synergy predictions in complex pharmacological networks	Predicted synergy score: 0.3876 (10-fold CV); *P*-value: 0.006 (Wilcoxon *vs*. baseline)	238,239
DeepDrug2	Learns embeddings from a signed, directed biomedical graph integrating germline-driven AD genes, pathways, drugs, and interactions; uses link prediction and cosine drug–gene scoring	Prioritizes repurposed AD drug candidates based on proximity to AD-risk genes in embedding space; validated with UK Biobank EHRs	Advantages: Integrates germline mutations and real-world EHR validation; robust link prediction. Disadvantages: Limited by drug usage data coverage in EHR	Not externally validated beyond UKB; GNN output not individualized	AUC: 0.98; AP: 0.98	240
GCNs	Perform graph-level classification on subject-specific bipartite graphs constructed from image–text similarity using VLM	Recognize *via* multimodal integration of MRI slices and textual embeddings	Advantages: Utilizes VLM (BLIP) for cross-modal feature fusion; models global patient–feature relationships. Disadvantages: Sensitive to image–text alignment quality; depends on pretrained VLM robustness	Constructing meaningful bipartite graphs; ensuring stable similarity-based graph representation	Accuracy: 0.870; Sensitivity: 0.860; Specificity: 0.880; AUC: 0.945 (ADNI test set)	241
MC-RVAE	Integrates VRNNs with shared latent space across imaging and cognitive channels for multimodal sequence modeling	Reconstructs missing modalities and disease progression modeling using longitudinal MRI and cognitive assessments	Advantages: Captures intra- and inter-modality dynamics; handles missing channels over time; provides interpretable latent representations. Disadvantages: Assumes fixed temporal spacing; performance sensitive to modality dropout	Learning robust latent trajectories; balancing modality-specific reconstruction with shared space regularization	MAE: 0.75 (vol→cog); 0.68 (cort→vol); 0.68/0.68/0.59 (vol/cort/cog full); outperforms GFA and KNN.	242
JDSNMF	Extracts shared latent features by factoring multi-omics data; improves AD prediction accuracy	Extracts latent disease-relevant features from multi-omics datasets for AD prediction	Advantages: Captures AD-related modules with biological interpretability. Disadvantages: Sensitive to omics heterogeneity and non-informative signals; requires tuning across multiple layers and regularization terms	High computational cost with deep architecture; limited scalability for cohorts with unbalanced or sparse omics profiles	AUC (GE+DM): 0.801 (AD/NL), 0.771 (MCI/NL); AUC (MRI+PET): 0.843 (MCI/AD), 0.973 (NL/AD); outperforms NMF and deep semi-NMF.	243
WGCNA	Analyzes proteomic data to identify protein modules linked to tau pathology	Identifies tau-related modules in neuroinflammation, mitochondria, cholesterol biosynthesis, and synapse function	Advantages: Identifies tau-associated protein modules linked to neuroinflammation, mitochondrial dysfunction, cholesterol biosynthesis, and synaptic loss; enables system-level mapping of tau pathology. Disadvantages: Sensitive to experimental noise and batch effects; limited resolution in distinguishing endogenous from transgenic tau signals	Integration of mouse and human proteomes; module preservation and interpretation across models and tissues	Pearson correlation; Module preservation (Z-summary)	244,245
NBP	Uses graph theory to represent molecular interactions and analyze their relationships	Model molecular interactions in AD and identify key disease drivers	Advantages: Integrates pharmacological and omics data; enables target prioritization and drug repurposing. Disadvantages: Relies on interaction data quality; limited interpretability in dense or dynamic networks	Integrating heterogeneous datasets; distinguishing causality from correlation	Clustering coefficient: 0.749; Density: 0.648; Avg. path length: 1.385; Drug–target graph: 1836 nodes, 2725 edges, avg. degree: 2.97	246
PPI networks	Model interactions between proteins and help identify molecular mechanisms and disease drivers	Study post-translational modifications and interactions in AD pathology	Advantages: Enable high-resolution detection of dynamic protein complexes; integrates diverse quantification strategies including label-free, SILAC, and TMT. Disadvantages: Sensitive to data quality and peak modeling accuracy; computationally intensive for large datasets	Disambiguating overlapping complexes; Limited curated databases	AUC: >0.81 (classifier); F1-score: reported; Pearson *r*: >0.9; Peak fitting *R*^2^: >0.85	247
Pathway enrichment models	Identify overrepresented or topologically relevant pathways from omics-derived gene/protein lists	Interpret transcriptomic, proteomic, metabolomic, and epigenomic data to reveal biological functions in AD	Advantages: Broadly applicable across omics types; facilitates biological interpretation. Disadvantages: Sensitive to background selection and annotation version; inconsistent across tools	Integrating diverse omics distributions; standardizing inputs and annotations		248
Bayesian networks	Allow probabilistic inference and handle missing data; identify causal relationships	Help in causal inference and understanding complex relationships between biomarkers in AD	Infer causal relationships and handle missing data in AD modeling	Computationally intensive for large datasets; needs domain knowledge for reliable results		249
TabPFN	Uses millions of synthetic datasets for pre-training; supports classification and regression *via* in-context learning	Predicts tabular outcomes across biomedical, chemical, and AD-related datasets	Advantages: Learns from diverse tasks; high performance with minimal tuning. Disadvantages: High memory usage; limited scalability to very large datasets	Scaling beyond 10k samples or 500 features; interpretability and hardware efficiency	Classification AUC: 0.95 (tuned), 0.94 (default); Accuracy (normalized): ∼0.91 Regression RMSE: −0.97; R^2^: >0.92; Other: robust to noise/missing/outliers	250
Integrative network modeling approach	Integrates coexpression, causal, and regulatory networks across multi-omics and brain regions	Identifies LOAD-related neuronal subnetworks and key drivers; discovery of drug candidates like NCH-51	Advantages: Captures multiscale regulatory circuits; enables in silico to *in vivo* validation. Disadvantages: Methodologically complex; requires large-scale, high-quality multi-omics datasets	Integrating heterogeneous omics and trait data; validating key drivers across systems and models		251
Computational network analysis	Integrate expression data with signaling networks using PCST to infer sustained signaling paths	Identify site-specific inflammatory mediators in AD brain regions	Advantages: Reveals non-obvious upstream–downstream signaling links. Disadvantages: Relies on expression data; ignores post-translational regulation	Inferring causality from partial transcriptomic data; modeling sustained signaling with incomplete inputs		34
WIMOAD	Uses five base classifiers and an MLP-based meta-model; integrates gene expression and DNA methylation profiles *via* weighted score fusion	NC *vs* AD, NC *vs* EMCI, EMCI *vs* LMCI, etc., using ADNI data; compared against IntegrationLearner and MOGLAM	Advantages: Outperforms single-omics and prior models; interpretable *via* SHAP; robust multi-omics fusion. Disadvantages: The Current model is limited to two omics types; relies on peripheral blood data	Limited access to proteomic or imaging data; incomplete mapping between blood biomarkers and brain pathology	Accuracy: 0.88; Sensitivity: 0.89; Specificity: 0.87; F1 Score: 0.88; MCC: 0.82; ROC-AUC: 0.90	227
Ensemble model (RF + bagFDA + glmnet)	Integrates RNA-Seq, microarray, proteomics, and miRNA data from 4089 AD/control samples; age-stratified analysis	Identifies age-specific AD hallmarks; classifies AD *vs*. controls using genes, proteins, miRNAs, and pathway-level features	Advantages: Robust across omics types and age groups; generalizable to external datasets. Disadvantages: High dimensionality; not yet validated for early clinical deployment	Integration bias across modalities; lack of clinical metadata; limited proteomics and miRNA availability	Accuracy: 0.97; Sensitivity: 0.98; Specificity: 0.96; F1 Score: 0.97; MCC: 0.94; ROC-AUC: 0.99	225
GNNRAI	Integrates transcriptomics and proteomics using prior knowledge graphs; applies GNNs and set transformer for feature extraction and modality alignment	Predicts AD status from brain tissue samples across 16 AD biodomains; identifies known and novel biomarkers using explainable attribution methods	Advantages: Incorporates biological priors; handles missing modalities; provides biomarker-level interpretation. Disadvantages: Computationally intensive; lacks direct clinical metrics	No inclusion of methylomics/metabolomics; absence of feature-wise interpretability beyond graphs; not validated in clinical settings		228
IMBALMED	Combines 25 classifiers across 8 learning families; applies two-level fusion; integrates clinical assessments, biospecimen, neuroimaging, and demographics	NC *vs* AD, NC *vs* AD *vs* MCI; early detection at 12, 24, 36, and 48 months; externally validated on ADNI4	Advantages: Robust across timepoints and classifiers; outperforms 9 state-of-the-art imbalance methods; generalizable. Disadvantages: High computational cost; requires modality completeness for full prediction	Complexity in training and modality fusion; missing data across modalities; limited external datasets with matched structure	G-mean: 0.974 for binary task; External validation Accuracy: 0.9688	252
WGAN-WP + distance penalty	Pre-train on external omics data and retrain *via* transfer learning; use progressive growing and L2-distance regularization; apply to class-imbalanced microarray and lipidomics datasets	Synthetic augmentation of minority class samples to balance datasets; improves HistGradientBoostingClassifier performance on HCC and CBGS data	Advantages: Handles high-dimensional omics with few samples; outperforms SMOTE/RO in complex datasets. Disadvantages: Computationally intensive; sensitive to *α* tuning	Mode collapse risk; no early stopping from AUC in real-world deployment; limited generalization across omics types	AUC (microarray): 0.76; AUC (lipidomics): >0.89	253
TAMPOR	Performs batch harmonization using ratio transformation and median polish; provides tunable modes; ensures robustness to platform, cohort, and batch heterogeneity	Corrects and integrates TMT-MS, LFQ-MS, Olink, and SOMAscan data across AD cohorts; CSF and plasma alignment	Advantages: Platform-agnostic; outlier-tolerant; enables multi-cohort, multi-platform omics harmonization. Disadvantages: Not a predictive model; no classification output	Not suitable for direct classification tasks; requires missingness <50%; complex parameter tuning for GIS-defective datasets		254
SVM + Relief-F + bivariate ranking	Classify samples using gene pairs across 3 hippocampal transcriptomic datasets; select non-DE genes robustly *via* cross-study aggregation; optimize using CV > 0.70 filtering	Identify gene pairs showing robust predictive power across datasets; discovery of synergistic gene associations missed by univariate DE analysis	Advantages: Interpretable gene-level biomarkers; generalizable multivariate ranking; robust across datasets. Disadvantages: No clinical classifier built; lacks pathway-level aggregation	Poor generalizability of some gene pairs; no AUC/Sensitivity reported; not suitable for individual prediction	CV Accuracy: 0.90 (VR); generalizability scores: VR–MAYO–GSE CV decline observed	41
MINDSETS	Integrates radiomics MRI features, genetic, clinical, and cognitive data using CNN-based DFG module; segmentation *via* SynthSeg; feature fusion and selection *via* FeatureWiz; interpretability *via* SHAP	AD *vs* VaD, AD *vs* MCI, multiclass dementia subtyping using ANMerge; temporal MRI monitoring for treatment response	Advantages: Outperforms baseline 3D CNN; enables explainable early diagnosis and treatment effect tracking. Disadvantages: Computationally intensive; generalizability yet to be tested across populations	Fusion of heterogeneous modalities; longitudinal MRI variability; limited external validation	Accuracy: 0.89; AUC: 0.84; F1-score: 0.81; Precision: 0.79; Sensitivity: 0.84	255
2D/3D CNN (InceptionV3)	Uses MK-6240 and AV-1451 tau PET with image augmentation and 5-fold CV; occlusion sensitivity and t-SNE for interpretability	Predicts cognitive impairment (NC *vs*. MCI/AD) using dual-radioligand tau PET; compares against SUVR metrics	Advantages: Outperforms ROI-based SUVR in AUC; interpretable *via* occlusion maps; generalizable to new radioligands. Disadvantages: No external validation; moderate gains in specificity	Lack of standardization for tau PET classification; computationally intensive 3D training; cross-ligand harmonization needed	Accuracy: >0.80; Sensitivity: 0.82; Specificity: 0.75; AUC: 0.89 (combined 2D CNN)	256
Hybrid	Combines CNN with RNN/attention mechanisms; integrates imaging, genomics, cognition; applies at multiple fusion levels	AD *vs* MCI *vs* NC classification; cross-modal early diagnosis; progression trajectory modeling	Advantages: Fuses spatial + temporal signals; high accuracy across tasks. Disadvantages: Complex architecture; requires large labeled multimodal datasets	Multimodal alignment; overfitting risk; computational load	Accuracy: 0.99; Sensitivity: 1; Specificity: 0.988; AUC: 0.99	257
Transformer	Applies to multimodal neuroimaging time series; models long-range dependencies and modality-level interactions	Risk stratification and cognitive decline prediction using MRI, PET, and CSF data	Advantages: High scalability; effective on long sequences; enables inter-modality attention. Disadvantages: Requires extensive data; computationally intensive	High training cost; interpretability limited *vs* simpler models	Accuracy: 0.968; Sensitivity: 0.960; Specificity: 0.965; AUC: 0.97	257
CNN	Applies to structural and functional MRI/PET; image-level and feature-level fusion; detects spatial AD biomarkers	AD *vs* MCI *vs* NC classification; structural MRI-based early AD detection	Advantages: Captures spatial features; widely validated; efficient with radiomics input. Disadvantages: Limited temporal modeling; requires large image datasets	Generalization across scanners and protocols; lack of multimodal integration	Accuracy: up to 0.99; Sensitivity: up to 0.98; Specificity: up to 0.98; AUC: up to 0.99	257
DeepPrep	Integrates DL-based neuroimaging pipeline with FastSurferCNN, FastCSR, SUGAR, SynthMorph; 83 steps managed *via* Nextflow; deployable on HPC/cloud/local	Preprocessing of >55,000 sMRI/fMRI scans (UKB, MSC, CoRR-HNU, clinical datasets); segmentation, registration, normalization	Advantages: 10× faster than fMRIPrep; full completion on clinical data; scalable and modular. Disadvantages: Not a predictive or diagnostic model; lacks omics integration	No disease classification; GPU/memory requirements; cannot support omics fusion tasks	Speed-up: 10.1×; Completion ratio: 100%; Dice: >0.85 (in 52/62 regions)	258
p-tau217 + NfL model	Use plasma biomarkers to predict tau PET positivity and AD status; interpretable logistic regression or ensemble models	Plasma-based AD diagnosis; screen for PET positivity	Advantages: Non-invasive; cost-efficient; near-imaging level accuracy. Disadvantages: Performance varies by platform/cohort	Assay variability; requires standardization across sites	AUC: 0.89	259
NeuroPM-box	Uses diffusion models and multiscale latent space modeling for simulating molecular propagation and brain trajectory	Disease progression simulation; AD subtype trajectory estimation; therapeutic response modeling	Advantages: Mechanistic interpretability; usable in precision medicine design. Disadvantages: Requires longitudinal multi-modal data; complex parameter tuning	Limited to cohorts with dense data; real-world deployment uncertain		259
MORE	Integrates mRNA, DNA methylation, and miRNA using fused hyperedge group; MOHE module extracts omics-specific features; MOSA module applies multi-head self-attention for modality integration	Classifies AD (ROS/MAP), BRCA, and GBM subtypes; biomarker identification across omics	Advantages: Encodes higher-order correlations *via* hyperedges; outperforms MOGONET, MOGLAM, MOADLN across tasks; robust ablation results. Disadvantages: Currently limited to omics; not extended to imaging or clinical data	Sample size imbalance across modalities; lacks modality generalization beyond omics	Accuracy: 0.829 (ROS/MAP); 0.835 (BRCA); 0.762 (GBM); F1: 0.836 (ROS/MAP); 0.820 (BRCA); 0.755 (GBM); AUC: 0.903 (ROS/MAP)	260
MAGMA + scDRS (in sc2GWAS)	Integrate GWAS summary statistics with large-scale scRNA-seq data; calculates cell-wise trait enrichment scores; supports ontology alignment and cross-species mapping	Identify AD-associated cell types (*e*.*g*., microglia) and driver genes (*e*.*g*., *APOE*, *TIMP1*, *BTG1*) using single-cell profiles; scalable to 784 GWAS traits	Advantages: Cell-type resolution; flexible trait input; interpretable gene prioritization. Disadvantages: Not a classifier; lacks multi-omics training	Dependent on GWAS quality and scRNA resolution; no integration of proteomics or epigenetics		261
MOSEGCN	Combines multi-head self-attention and SNF to learn intra-/inter-omics latent features; uses semi-supervised SEGCN to leverage labeled and unlabeled data	Disease subtype classification on AD (ROS/MAP), BRCA, GBM; biomarker identification	Advantages: Integrates latent omics interactions; outperforms MOGONET and SEGCN; interpretable biomarkers. Disadvantages: Limited to 3 omics types; not tested on clinical/imaging data	Lacks cross-modal validation; sensitive to attention-head number; limited scalability	Accuracy: 0.83 (ROS/MAP), 0.87 (BRCA), 0.89 (GBM); AUC: 0.832 (ROS/MAP); F1: 0.827 (ROS/MAP), 0.868 (BRCA), 0.890 (GBM)	262
Federated LSTM with FedAvg	Trains LSTM models on site-partitioned EHRs without sharing raw data; uses FedAvg and personalized fine-tuning; incorporates SHAP and PFI for interpretation	Predicts MCI-to-AD progression across six sites in OneFlorida+ EHR consortium; compares personalized, pooled, global, and local models	Advantages: Preserves privacy; handles site heterogeneity; interpretable *via* SHAP. Disadvantages: Lacks multimodal input (no imaging/omics); only uses structured EHRs	Limited to structured EHR data; excludes unstructured notes or imaging; no comparison to deep multimodal fusion models	AUC: Personalized: 0.794 (North FL); 0.744 (South FL); Global: 0.837 (South FL); Local: 0.729–0.820 (by site)	263
Tri-COAT	Learns joint representation of imaging, genotype, and clinical data *via* modality-specific transformers and tri-modal co-attention; supports early subtyping using baseline-only data	Subtypes of AD patients into slow, intermediate, and fast cognitive decline using baseline ADNI data	Advantages: Baseline-only input; interpretable co-attention maps; identifies cross-modal biomarkers. Disadvantages: Limited to MMSE-based subtypes; lacks multi-cohort validation	Model trained on ADNI only; performance sensitive to class imbalance (fast subtype: *n* = 15); lacks PET or transcriptomic input	AUROC: 0.734 ± 0.076 (3-class, macro average); Imaging: 0.648; Genetics: 0.539; Clinical: 0.697	264
scGraph2Vec	Enhances variational graph autoencoder by integrating gene–gene networks and single-cell expression data; models community structure with modularity-aware loss	Identifies functional gene clusters across brain, heart, liver, lung, kidney, PBMCs; infers disease-related genes using AD and COVID-19 GWAS seed genes	Advantages: Captures both topological and expression-based gene features; interpretable latent space; broad tissue applicability. Disadvantages: Requires high-quality network data; gene-centric, not patient-centric	Lacks validation on external clinical cohorts; not designed for patient-level diagnosis or prediction		265
MADDi	Uses modality-specific DNN backbones and combines *via* self-attention and cross-modal attention; integrates MRI, SNPs, and structured clinical data	3-class classification (NC *vs* MCI *vs* AD) using ADNI dataset; evaluates attention mechanisms, modality contribution, and model robustness	Advantages: High accuracy; interpretable *via* attention; strong multimodal performance. Disadvantages: Requires matched multimodal data; no time-series or PET input	Small overlap set (*n* = 239); diagnosis labels vary across modalities; imbalance in fast-decline class	Accuracy: 0.97; F1-score (overall): 0.91; F1-score (AD): 1.00; F1-score (MCI): 0.77; F1-score (NC): 0.98	266
Federated GEIDI	Integrates sMRI, GWAS SNPs, and gene expression in a federated framework; uses stratified Chow test to detect genotype-dependent expression–imaging relationships	Identifies AD-related gene expression and SNPs stratified by *APOE* and rs942439 in ADNI; evaluates associations with hippocampal and mid-temporal volumes	Advantages: Preserves data privacy; reveals SNP-dependent transcriptomic correlates; outperforms Matrix eQTL. Disadvantages: No patient-level classification; slow computation	Requires pre-stratification by genotype; lacks multi-class prediction; limited to two imaging ROIs	TPR: 0.61 (*vs*. 0.55 by Matrix eQTL); *P*-values for hypergeometric enrichment: 0.00039 (hippocampus), 0.00624 (mid-temp)	267
BIONIC	Integrates proteomics and PPI data using GAT; outputs 512-dimensional features per protein; followed by Louvain clustering and MINDy-based moderator inference	Identifies *APP–MAPT–HSPA5* subnetwork; reveals moderators (*GPNMB*+ microglia, *GFAP*+ astrocytes) of A*β*–tau interaction in early-stage AD	Advantages: Captures latent topological relationships; identifies glia-enriched subnetworks; interpretable *via* modular enrichment. Disadvantages: No individual-level prediction; model trained at the protein-level	No external benchmarking; not validated on other modalities; lacks temporal prediction		53
Hybrid multimodal DNN + SVR	DenseNet3D + self-attention + AE modules extract image features (hippocampus & ATL); non-image inputs (MMSE, ADAS-Cog, APOE, A*β*42, p-tau) combined for SVR prediction of CDR-SB change	Predict cognitive decline (CDR-SB) over 2 years in NA-ADNI; used for stratified randomization in clinical trial simulation	Advantages: Robust to limited sample size; reduces randomization bias; supports personalized trial design. Disadvantages: Limited to MCI/AD; higher error in extreme cases	Noise in CDR-SB targets; limited to NA-ADNI; no external trial validation	MAE: 1.07; Correlation: 0.58 (overall); Inner samples: MAE: 0.89; Corr: 0.62	268
NeuroPM-cTI	Uses contrastive PCA + stratification to infer pseudotemporal progression and disease subtypes; integrates omics, imaging, and histopathology	Applies to ADNI, ROS/MAP, HBTRC; stratifies subjects into molecular sub-trajectories and progression scores; validates across AD, HD, and controls	Advantages: Interpretable scores, flexible multimodal inputs; robust to noise. Disadvantages: No direct predictive output	Requires tuning and quality-controlled inputs; pseudo-longitudinal inference only	FWE-corrected ANOVA *F* = 11.17 (Braak); *F* = 9.04 (Vonsattel)	269
LASSO-based 10-protein AD prediction model	Selects 10 CSF proteins *via* 50-iteration LASSO regression with 70/30 split; trained on SomaScan 7K proteomics; validates across 3 cohorts	Classifies A+T+ *vs* A–T–; predicts clinical AD diagnosis and amyloid PET status; applies to blood plasma with retraining	Advantages: High AUC across multiple cohorts; consistent across platforms; longitudinal prediction. Disadvantages: Requires CSF; protein weights are not transferable to plasma	Platform-specific training required; limited power in non-AD dementia	AUC: >0.98 (biomarker); 0.89–0.97 (clinical); 0.90 (PET); Sensitivity, Specificity, PPV, NPV >0.88	270
RF with SHAP-based explanation	Multimodal input (clinical, psychological, MRI segmentation); data-level fusion; SMOTE balancing; 10-fold stratified CV; SHAP for interpretation	Predicts 5-class AD status (NC, AD, Non-AD, Uncertain, Others) using OASIS-3 data; identifies key features (Memory, Sumbox, Judgment, IntraCranialVol)	Advantages: High accuracy, explainable, interpretable SHAP outputs. Disadvantages: Limited generalizability beyond OASIS; lacks external validation	Dataset imbalance (solved *via* SMOTE); not compared with DL models	Accuracy: 0.99; Precision: 0.99; Sensitivity: 0.99; F1-score: 0.99; AUC: 1.00	271
Transformer-based multimodal diagnostic model	Integrates demographics, medical history, neuropsychological tests, and multisequence MRI with modality-specific embeddings and attention-based transformer	Differential diagnosis of 13 dementia etiologies (AD, VaD, LBD, FTD, etc.) across 9 cohorts; predicts NC, MCI, dementia status and disease probabilities	Advantages: Handles missing data; aligns with biomarker and autopsy; supports AI-augmented diagnosis. Disadvantages: Some classes are underrepresented; limited staging	Performance is lower for rare etiologies; dataset imbalance	AUROC: 0.96 (micro), 0.94 (weighted); AUPR: 0.70 (micro); AI-augmented AUROC: 0.2625, AUPR: 0.7323	272
VBpMKL + MCI subtyping	Integrates sMRI, SNPs, and mRNA expression; subtypes *via* SNF; feature selection by Lasso	Predicts MCI-to-AD conversion in 3 years using ADNI-1 (*n* = 125) and ADNI-GO/2 (*n* = 98) datasets; internal and external validation performed	Advantages: Improves accuracy *via* subtype-specific models; multi-modality integration enhances prediction and interpretability. Disadvantages: Computationally intensive; requires large, well-annotated omics datasets	Model complexity; performance varies across subtypes; generalization is limited without large-scale validation	AUC (subtype I): 0.8581; AUC (subtype II): 0.8623; AUC (overall): 0.83; Accuracy: 0.79; Sensitivity: 0.81; Specificity: 0.78	273
MCAD	Imaging (cascaded dilated convolutions) and CSF (sinusoidal functions) encoders. Cross-attention for MI. The objective function combines classification and modality alignment loss	AD, MCI, NC diagnoses on ADNI dataset. Utilizes sMRI, FDG-PET, and CSF data. Multi-class classification tasks for AD, MCI, and NC	Advantages: Efficient, accurate AD diagnosis *via* inter-modality interactions, reduced discrepancy. Disadvantages: High complexity when extending cross-modal attention to different imaging modalities increases overfitting risk	Effectively capture inter-modality interaction. Reduce modality discrepancy. Bridge the heterogeneity gap between imaging and CSF	AD *vs*. NC: ACC: 0.9107 ± 0.0382 SEN: 0.9103 ± 0.0478 SPE: 0.9107 ± 0.0647 F1: 0.9111 ± 0.0381 AUC: 0.9407 ± 0.0316; AD *vs*. MCI *vs*. NC: ACC: 0.6403 ± 0.0249 SEN: 0.6385 ± 0.0241 SPE: 0.8200 ± 0.0124 F1: 0.6185 ± 0.0158 AUC: 0.7677 ± 0.0422	274

Note: A*β*, amyloid beta; ACC, accuracy; AD, Alzheimer's disease; ADAS-Cog, Alzheimer's Disease Assessment Scale – Cognitive Subscale; ADNI, Alzheimer's Disease Neuroimaging Initiative; AE, autoencoder; AI, artificial intelligence; ALP, autophagy–lysosome pathway; ANN, artificial neural network; ANOVA, analysis of variance; *APOE*, apolipoprotein E; AP, Average Precision; AUC, area under the ROC curve; AUPR, area under the precision recall curve; AUROC, area under the receiver operating characteristic curve; BN, Bayesian probabilistic causal Network; BRCA, breast cancer; CBGS, Cambridge Baby Growth Study; CDR-SB, Clinical and Dementia Rating – Sum of Boxes; CGMH, Chang Gung Memorial Hospital; CUI, clinical utility index; CoRR-HNU, Consortium for Reliability and Reproducibility Hangzhou Normal University; CSF, cerebrospinal fluid; CV, cross-validation; DBNs, deep belief networks; DE, differentially expressed; DFG, Directed Fusion Graph; DL, deep learning; DM, DNA methylation; DNAm, DNA methylation; DNN, deep neural networks; EHRs, electronic health records; EMCI, early mild cognitive impairment; FedAvg, federated averaging; FDR, false discovery rate; FDG, fluorodeoxyglucose; FWE, family-wise error; GAN, generative adversarial networks; GAT, graph attention network; GBM, glioblastoma multiforme; GCNs, graph convolutional networks; GE, gene expression; GFA, group factor analysis; GIS, global internal standard; GNNs, graph neural networks; GNNRAI, GNN-derived Representation Alignment and Integration; GWAS, genome-wide association studies; HD, Huntington's disease; HCC, hepatocellular carcinoma; HDLSS, high-dimensional, low-sample-size; HPC, high-performance computing; HSPA5, heat-shock-protein family A member 5; IMBALMED, iMbalancEd Data; JDSNMF, joint deep semi-non-negative matrix factorization; KNN, K nearest neighbors; LASSO, least absolute shrinkage and selection operator; LBD, Lewy body dementia; LFQ-MS, label-free-quantitation mass spectrometry; LMCI, Late Mild Cognitive Impairments; LSTM, long short-term memory; MAE, mean absolute error; MAGMA, multi-locus analysis of genomic architecture; MAPT, microtubule-associated protein-tau; MCAD, multi-modal cross-attention AD diagnosis; MCC, Matthews correlation coefficient; MCI, mild cognitive impairment; MC-RVAE, multi-channel recurrent variational autoencoder; MDP, Markov decision process; MILP, mixed-integer linear programming; MINDy, Modulator Inference by Network Dynamics; MINDSETS, Multi-omics Integration with Neuroimaging for Dementia Subtyping and Effective Temporal Study; miRNA, microRNA; MLP, multilayer perceptron; MMSE, Mini-Mental State Examination; MOADLN, Multi-Omics Attention Deep Learning Network; MOGLAM, Multi-Omics Graph Learning and Attention Mechanism; MOGONET, Multi-Omics Graph cOnvolutional NETworks; MOHE, Multi-Omics Hypergraph Encoding; MORE, Multi-Omics hypeRgraph integration nEtwork; MOSA, Multi-Omics Self-Attention; MOSEGCN, Multi-Omics Semi-Supervised Edge-to-Graph Convolutional Network; mRNA, messenger RNA; MSC, Midnight Scan Club; M-LSTM, multi-dimensional spatiotemporal long short-term memory; NAB, neocortical amyloid burden; NACC, National Alzheimer's Coordinating Center; NA-ADNI, North-American Alzheimer's Disease Neuroimaging Initiative; NB, Naive Bayes; NBP, network-based approach; NC, normal control; NCH-51, drug candidate NCH-51; NeuroPM-box, Neuroinformatics for Personalized Medicine toolbox; NfL, neurofilament light; NL, normal; NMF, non-negative matrix factorization; OASIS, Open Access Series of Imaging Studies; OOB, out-of-bag; PBMCs, peripheral blood mononuclear cells; PCST, Prize Collecting Steiner Tree; PFI, permutation feature importance; p-tau, phosphorylated tau; p-tau217, phosphorylated tau at threonine 217; PPI, protein–protein interaction; RBM, restricted Boltzmann machine; RF, random forest; RNA-Seq, RNA sequencing; ROS/MAP, Religious Orders Study and Rush Memory and Aging Project; RNN, recurrent neural network; RMSE, root mean square error; scDRS, single-cell Disease Relevance Score; scRNA-Seq, single-cell RNA sequencing; SEGCN, self-ensembling Graph Convolutional Network; SHAP, SHapley Additive exPlanations; SILAC, stable isotope labelling by amino acids; SMOTE, synthetic minority over-sampling technique; SNPs, single-nucleotide polymorphisms; SPE, specificity; SUGAR, spherical ultrafast graph attention framework for cortical surface registration; SUVR, standardized uptake value ratio; SVM, support vector machines; SVR, support vector regression; TAMPOR, tunable median polish of ratio; t-SNE, t-distributed stochastic neighbor embedding; TIMP1, tissue inhibitor of metalloproteinases-1; TMT, tandem-mass-tag; TMT-MS, tandem-mass-tag mass spectrometry; TPR, true-positive rate; UKB, UK Biobank; VaD, vascular dementia; VBpMKL, variational Bayes approximation with probabilistic multiple kernel learning; VLM, vision–language model; vol, brain volume; VRNN, variational recurrent neural network; WGAN-WP, Wasserstein generative adversarial network with weight penalty; WGCNA, weighted gene co-expression network analysis; WIMOAD, weighted integration of multi-omics data for Alzheimer's disease; XGBoost, extreme gradient boosting; SNF, Similarity Network Fusion; sMRI, structural magnetic resonance imaging; fMRI, Functional MRI; PET, positron emission tomography; GEIDI, Genotype-Expression-Imaging Data Integration.

### 4.1 Machine learning approaches

Machine learning methods have become indispensable for integrating and analyzing multi-omics datasets in AD research. These approaches provide robust frameworks for managing the inherent complexity, heterogeneity, and high dimensionality of omics data, while uncovering meaningful biomarkers and therapeutic targets.

Established machine learning techniques such as support vector machines (SVMs), random forests, gradient boosting, and elastic-net regularized regression have been successfully applied to integrate multi-omics data with clinical and neuroimaging variables. These methods are particularly well-suited for capturing etiological and clinical heterogeneity in AD, supporting both disease stratification and individualized prediction. Supervised learning approaches, including SVMs and random forests, have demonstrated high performance in differentiating AD phenotypes from controls. For example, modularity-constrained logistic regression applied to the Religious Orders Study and Rush Memory and Aging Project (ROS/MAP) cohort identified functionally connected single-nucleotide polymorphisms (SNPs), genes, and proteins predictive of AD, and linked these molecular features to neuroimaging and cognitive outcomes^275^. Similarly, random forest classifiers have highlighted plasma p-tau and *α*-synuclein as predictive biomarkers of progression from mild cognitive impairment (MCI) to dementia, findings that have been experimentally validated^276^. Elastic-net models further prioritize cross-omic biomarkers, demonstrating the value of ensemble strategies in AD research^277^. Graph-based methods, such as graph convolutional networks (GCNs), enable the modeling of intricate inter-omic relationships. The staged GCN with uncertainty quantification (SGUQ) has integrated genomic, proteomic, and clinical data while reducing computational overhead and maintaining predictive accuracy^278^. These approaches align well with biological systems, where molecular entities and interactions are inherently network-structured. In addition, Bayesian factor analysis and sparse canonical correlation analysis (SCCA) address data heterogeneity by uncovering shared molecular signatures and subgroup-specific drivers. For instance, SCCA has revealed immune-related expression patterns in RNA-Seq datasets from specific brain regions, offering biologically interpretable insights into disease progression^279^, ^280^, ^281^.

Despite recent progress, several methodological challenges limit clinical translation. A major concern is the limited interpretability of many machine learning models, particularly deep neural networks and ensembles. Although explainable AI (XAI) techniques such as SHapley Additive exPlanations (SHAP), Local Interpretable Model-Agnostic Explanations, and layer-wise relevance propagation are gaining traction, comparative studies have shown inconsistent outputs across methods, highlighting the need for application-specific validation^282^. Class imbalance in public datasets also affects sensitivity. Most cohorts include more cognitively normal or mildly impaired individuals than confirmed AD cases, skewing predictions. Techniques like the IMBALMED framework, which ensembles models using various resampling or cost-sensitive learning strategies, have improved F_1_ scores in the Alzheimer's Disease Neuroimaging Initiative (ADNI) dataset. Generative adversarial oversampling has similarly reduced false negatives, though strict controls are required to avoid data leakage^252^^,^^253^. Inconsistent preprocessing also hinders reproducibility. Decisions about normalization or batch correction can dramatically alter biomarker rankings. Tools such as TAMPOR, which apply standardized, containerized workflows, help address this. However, fewer than one-third of published multi-omics AD studies release full code and preprocessing parameters, limiting independent validation^254^. Overfitting remains a persistent issue due to the imbalance between feature dimensionality and sample size. Nested cross-validation has mitigated this problem in transcriptomic classifiers, increasing external accuracy by up to 15%^283^. Case studies have illustrated both the promise and limitations of machine learning in multi-omics AD research. One study combining proteomic and metabolomic data identified dysregulated sphingolipid and ceramide metabolism in *APOE ε4* carriers, findings supported by post-mortem analyses. However, predictive accuracy declined in ethnically diverse cohorts, underscoring the importance of ancestry-inclusive validation^41^^,^^159^^,^^284^

Looking forward, clinically viable models will require a combination of robust explainability tools, harmonized preprocessing protocols, balanced datasets, and multi-site validation. Initiatives such as the MINDSETS project, which integrates omics, imaging, and cognitive data across international cohorts, exemplify the type of transparent and scalable framework needed to transition machine learning prototypes into real-world diagnostic and prognostic applications^255^^,^^282^.

### 4.2 Deep learning architectures

Deep learning has emerged as a powerful approach for integrating and analyzing multi-omics datasets, offering unprecedented insights into the molecular mechanisms underlying AD. By harmonizing genomics, transcriptomics, proteomics, and epigenomics data, deep learning models provide a comprehensive and scalable framework that overcomes limitations of traditional single-omics methods.

Several innovative architectures have shown promise in addressing the high-dimensional, low-sample-size (HDLSS) nature of multi-omics data. Joint Deep Semi-Non-Negative Matrix Factorization (JDSNMF) captures shared latent features across omics layers, enabling the identification of biologically relevant modules for disease prediction^243^. Deep Belief Networks (DBNs) integrate gene expression and methylation features to improve classification accuracy, while Explainable Variational Autoencoders (E-VAEs) reveal interpretable genomic-transcriptomic interactions linked to AD pathways^237^^,^^285^. Advanced models such as the multi-omics data fusion network (MoFNet) incorporate prior biological knowledge to dynamically link SNPs, genes, and proteins. This facilitates the identification of subnetworks involved in processes like neuroinflammation and synaptic dysfunction^286^. Additionally, scGraph2Vec uses graph neural networks to extract biologically meaningful gene embeddings from single-cell omics, elucidating tissue-specific regulatory mechanisms^265^. Generative models like CE-GAN simulate brain network evolution and have achieved high accuracy in risk prediction^287^. Furthermore, frameworks like SDGCCA capture non-linear correlations across omics modalities, facilitating the discovery of robust cross-layer biomarkers^288^.

Despite their predictive power, deep learning models often suffer from limited interpretability. Convolutional neural networks applied to PET scans may achieve high area-under-the-curve scores, but their saliency maps frequently fail to align with known pathological regions, reducing clinician trust. Techniques such as attention layers, SHAP, and patch-based visualizations aim to localize voxel- or gene-level contribution, yet benchmarking studies indicate that their outputs can be inconsistent and model-dependent^256^^,^^282^^,^^289^. Deep learning frameworks are also computationally intensive. Multimodal imaging workflows, such as those harmonized by DeepPrep, often require terabytes of storage and high-end GPU infrastructure^258^. Although model-parallel optimization and memory-efficient training have reduced some of these burdens, resource constraints remain a significant barrier, particularly for smaller research teams^290^^,^^291^. Overfitting is another concern, driven by the disparity between the number of features and available samples. Techniques such as nested cross-validation, dropout, early stopping, and weight decay have been effective in mitigating overfitting and improving external prediction accuracy by up to 15%^257^^,^^292^. Application-specific studies illustrate both the strengths and limitations of deep learning. A study integrating metabolomics and proteomics in *APOE ε4* carriers revealed disruptions in sphingolipid and glycerophospholipid pathways. However, the kernel-based model used was non-transparent and performed poorly in non-European cohorts, highlighting the challenge of cross-population generalizability^159^^,^^293^. This limitation is common in AD research. Predictive signatures derived from ADNI datasets often underperform in community-based cohorts that differ in ancestry, education level, and comorbid conditions^259^. Although the ADNI-4 initiative aims to address demographic imbalances, most current models lack external validation in diverse populations. This raises concerns about their robustness and real-world applicability^294^^,^^295^.

To enhance the translational relevance of deep learning in AD, future efforts must prioritize interpretability, reproducibility, and validation. Embedding XAI mechanisms directly within model architectures can improve alignment between feature attributions and predictive objectives, thereby facilitating clinical interpretation and regulatory confidence. At the same time, releasing standardized, containerized preprocessing workflows with complete parameter documentation is essential to ensure methodological transparency and enable reproducibility across studies. Validation using large, prospective, and demographically diverse cohorts is equally critical, as many current models struggle to generalize beyond homogenous populations. To address these limitations, biologically informed architectures, such as graph neural networks that encode pathway-level priors, offer a promising avenue for integrating domain knowledge while preserving predictive performance^228^. These interpretable and integrative deep learning frameworks have the potential to form a robust foundation for AI-driven diagnostic and therapeutic tools in AD, ultimately bridging the gap between complex multi-omics analyses and clinically actionable insights.

### 4.3 Network-based methods

Network-based approaches have become indispensable in integrating multi-omics data to elucidate the complex molecular mechanisms of AD. These methods represent biological entities such as genes, proteins, or metabolites as nodes, and define their interactions as edges, thereby facilitating a systems-level understanding of disease pathogenesis^237^^,^^251^^,^^296^^,^^297^.

Early implementations often relied on correlation-based networks that inferred pairwise associations among features. While these models were straightforward and interpretable, they could not capture higher-order interactions or context-specific dependencies^298^. Subsequently, methods such as Weighted Gene Co-expression Network Analysis (WGCNA) enhanced network granularity by identifying modules of highly co-expressed genes, offering biologically relevant insights into co-regulated pathways implicated in AD^244^^,^^299^. However, WGCNA's reliance on linear correlations and scale-free assumptions has drawn criticism for oversimplifying the nonlinear and heterogeneous nature of molecular interactions^300^. To address these limitations, recent advancements have incorporated kernel-based and mutual information-based metrics, uncovering hidden relationships missed by linear models^301^^,^^302^. Integration with protein–protein interaction (PPI) networks has further refined AD models by connecting transcriptomic and proteomic layers, particularly when combined with tissue-specific data or prior biological knowledge^303^, ^304^, ^305^. Bayesian networks and Markov random fields offer probabilistic frameworks capable of modeling causal relationships under uncertainty, although these approaches are computationally intensive and often require extensive priors for convergence^306^^,^^307^. More sophisticated strategies involve multi-layer or multiplex network models that represent each omics layer as a distinct stratum and encode cross-layer relationships to capture inter-omic dependencies^308^^,^^309^. These architectures reveal interactions such as the link between genetic variants and metabolic alterations, but their practical application is often constrained by computational demands and sensitivity to parameter choices^251^^,^^310^^,^^311^. Single-cell data integration has introduced new opportunities for cell-type-specific network inference. Tools like sc2GWAS connect genome-wide association loci to cellular transcriptomic signatures, identifying microglia and astrocytes as key mediators in AD^151^^,^^261^. Deep learning-based approaches, including Deep Belief Networks and Graph Neural Networks (GNNs), have further expanded the capacity to model nonlinear, high-dimensional interdependencies across omics layers^237^^,^^312^^,^^313^.

Despite their promise, several barriers limit the clinical translation of network-based models. First, interpretability remains a core challenge. High-performing architectures such as GNNs and deep generative models often provide limited transparency regarding feature attribution, and existing explainability tools such as SHAP or attention mechanisms yield inconsistent results^228^^,^^314^^,^^315^. Second, computational accessibility poses a major hurdle. Multi-layer network construction and inference often require high-performance infrastructure, restricting participation to well-resourced institutions^260^. Third, reproducibility is hindered by variability in preprocessing pipelines, parameter tuning, and network construction choices. Small changes in normalization, imputation, or similarity metrics can substantially alter network topology and derived biological insights^316^^,^^317^. Interpreting network modules introduces additional complexity. Statistically robust clusters may not align with known biological pathways and can group unrelated genes. Moreover, commonly used backbone networks like PPIs may include context-agnostic interactions, confounding functional interpretations. For instance, findings from the Accelerating Medicines Partnership–Alzheimer's Disease (AMP-AD) consortium originally attributed to immune-metabolic hubs were later found to reflect cell composition shifts rather than genuine regulatory networks^318^^,^^319^. Single-cell network inference holds potential to resolve these ambiguities but faces its own limitations, including noise sensitivity and reliance on aggressive data denoising strategies^261^^,^^315^.

To overcome these challenges, the field is advancing toward standardized and collaborative solutions. Initiatives such as AMP-AD and ADNI now promote open-source software, harmonized preprocessing workflows, and public sharing of network outputs to facilitate benchmarking and reproducibility. Hybrid frameworks that embed mechanistic priors into machine learning models, such as GNNs enriched with biological knowledge graphs, offer a path to balancing predictive power with interpretability^228^. For network-based approaches to achieve clinical relevance, future efforts must include rigorous external validation across diverse populations, collaborative iteration between computational scientists and experimental biologists, and equitable access to computational infrastructure. These efforts will be essential for translating network-derived insights into actionable tools for early diagnosis, mechanistic understanding, and therapeutic targeting in AD.

## 5 AI-driven multi-omics applications in AD

The integration of AI with multi-omics technologies has significantly advanced AD research by enabling comprehensive and high-resolution analyses of its intricate molecular landscape. These integrative approaches have shown considerable promise across key domains, including early diagnosis, mechanistic elucidation, biomarker discovery, therapeutic development, and patient stratification (Fig. 4, Table 3^25^^,^^53^^,^^58^^,^^226^^,^^237^^,^^251^^,^^253^^,^^255^^,^^261^, ^262^, ^263^, ^264^, ^265^, ^266^, ^267^, ^268^, ^269^, ^270^, ^271^, ^272^, ^273^, ^274^^,^ ^276^^,^^278^^,^^283^^,^^287^^,^^289^^,^^319^, ^320^, ^321^, ^322^, ^323^, ^324^, ^325^, ^326^, ^327^, ^328^, ^329^, ^330^, ^331^, ^332^, ^333^, ^334^, ^335^, ^336^, ^337^, ^338^, ^339^, ^340^, ^341^, ^342^, ^343^, ^344^, ^345^, ^346^, ^347^, ^348^, ^349^, ^350^, ^351^, ^352^, ^353^, ^354^, ^355^, ^356^, ^357^, ^358^, ^359^, ^360^, ^361^, ^362^, ^363^, ^364^).

**Table 3:** The application of AI models in the integration of multi-omics for AD research.

AI models	Clinical application and outcomes	Key advances	Challenges and future directions	Evaluation metrics	Ref
B-HEALED	Clinical: 345 individuals from 7 cohorts; distinguish 249 AD (82 prodromal, 83 mild) from 96 non-AD disorders; follow-up showed MMSE decline and CDR increase; 82.9% received CSF or PET imaging, with 61.9% amyloid-positive; NP: MMSE decline (−2.7/year) and CDR increase (+0.3/year) during follow-up	Integrates 19 blood-based proteins and metabolites with age to achieve high diagnostic specificity across multicenter cohorts	Validates in larger, diverse cohorts; standardizes assays; assesses longitudinal performance	Specificity: 0.93 (internal), 0.92 (external) Sensitivity: 0.654 (internal), 0.524 (external) AUC: 0.819 (internal), 0.718 (external)	320
RF; SVM	Clinical: 273 AIBL and 82 ADNI participants; predicts high *vs*. low neocortical A*β* burden; validated against PET SUVR threshold and applied to non-imaged AIBL subjects; NI: PET SUVR	Identify an 8-marker blood panel predictive of A*β* status across cohorts	Address missing biomarker data; scale to population-level screening	AUC: 0.84 (RF), 0.84 (SVM) Sensitivity: 0.80 (RF), 0.74 (SVM) Specificity: 0.79 (RF), 0.76 (SVM)	321
DBN	Clinical: 838 subjects (513 AD, 325 controls) from multi-omics datasets; classifies AD *vs*. control	Combines DEGs and DMPs using Jaccard similarity; applies ensemble feature selection and Bayesian-optimized DBN for AD classification	Harmonize multi-omics across platforms; expand access to longitudinal data	Accuracy: 0.8466 (internal, 5-fold CV), 0.82 (external)	237
CE-GAN	Clinical: LMCI subjects from ADNI; predicts progression risk from stable to prodromal AD using fMRI and SNP data; NI: fMRI ROI	Integrates ROI-gene community networks with entropy-guided GAN evolution; identifies risk ROIs and genes relevant to AD progression	Limited multimodal sample size; requires broader validation and interpretability improvements	Accuracy: 0.9167; Sensitivity: 0.8967; Specificity: 0.9474; AUC: 0.9183	287
LR; RF; SVM	Clinical: 116 subjects from Taipei-VGH (49 AD, 28 P-MCI, 39 S-MCI); predicts progression from MCI to AD using blood transcriptomics and cognitive tests including MMSE, WMS-LM, CVVLT, TMT-B, TY-CFT, BNT, VF	Identify an 8-gene blood transcriptomic signature to predict MCI conversion; highlight mitochondrial and sex-specific pathways	Validate in larger, diverse cohorts; explore mechanistic underpinnings	Accuracy: 0.85 (AD *vs*. S-MCI), 0.88 (AD *vs*. P-MCI) AUC: 0.94 (AD *vs*. S-MCI), 0.92 (AD *vs*. P-MCI)	283
SGUQ	Clinical: 351 subjects from ROS/MAP (182 AD, 169 NC); Classify AD *vs*. NC using integrated mRNA, DNA methylation, and miRNA data	Integrates mRNA, DNA methylation, and miRNA *via* staged GCN with uncertainty quantification; enables cost-efficient diagnosis through selective omics layering	Require larger datasets and improved scalability for clinical translation	Accuracy: 0.858 (tri-omics), 0.8018 (mRNA-only) F1: 0.85 (tri-omics) AUC: 0.86 (tri-omics), 0.80 (mRNA-only)	278
MPxgb	Clinical: In silico prediction and experimental validation using human AD iPSNs, postmortem brain tissue, and inflammation-induced tau phosphorylation assays	Identifies five AD-risk genes; *CRTAM* and *SCGB3A1* linked to *TREM2*–*TYROBP* and IL–1*β*–TNF*α* immune pathways; nine genes reduced tau phosphorylation *via* siRNA knockdown	Address noisy negative training data; validate findings in broader human populations and multi-omics platforms	AUC-ROC: 0.91 (CV, 0.93 (test set) Sensitivity: 0.87 Specificity: 0.80	322
NETTAG	Clinical: In silico prediction and validation using AD GWAS loci, transcriptomic and proteomic datasets, and EHR-based population analyses	Integrates GWAS and nine regulatory features into PPI networks to identify 156 AD-risk genes enriched in immune and druggable pathways; validates expression in AD brains and glia; identifies repurposable drugs, including gemfibrozil	Address limitations in PPI completeness and population-specific GWAS data; enhance interpretability and validation across brain-specific omics	AUC: 0.81 (AlzGene); 0.80 (DistiLD); 0.72 (DISEASES-knowledge); 0.78 (TIGA)	323
LR; RF; SVM; MLP	Clinical: 386 MCI participants (93 cMCI, 103 sMCI); predicts progression risk using proteomic, metabolomic, and cognitive features, including MMSE and priority language z-scores	Identify oleamide as a lipid marker secreted by activated microglia; uncover inflammation and protein degradation pathways involving CFP, PI15, SNCA, and JPH3	Validate in independent cohorts; investigate the mechanistic role of oleamide and microglial EVs in neuroinflammation	Accuracy: 0.882 (SVM, proteins), 0.854 (SVM, metabolites) AUC: 0.64 (cMCI *vs*. sMCI) Sensitivity: 0.614 Specificity: 0.582	276
EnsembleOmicsAE	Clinical: 559 postmortem brain samples from Banner, ROS/MAP, and MSBB (AD, MCI, control); model trained on control *vs*. AD groups	Identifies AE signaling modules through ensemble autoencoders; reveals age-specific regulation of integrin signaling, cell adhesion, and neuronal differentiation pathways involving *VIM*, *MAPK1*, and *MAPK3*	Limited integration with genetic data; requires functional validation and optimization for clinical interpretability		324
Bayesian clustering with digital deconvolution	Clinical: 278 postmortem AD brain samples (255 AD, 23 control); identify 4 AD molecular subtypes with distinct clinical and neuropathological features	Identifies Knight-C4 subtype with severe neuronal loss, astrogliosis, reduced metabolomic signatures, and dysregulation in synaptic, lysosomal, and mTOR pathways	Expands multi-ethnic cohorts; validates subtype-specific biomarkers for clinical staging and precision therapy	DESeq2 FDR <0.05 for DE gene analysis	58
TransComp-R	Clinical: 80 AD and 173 control subjects (human postmortem hippocampus); model integrates transcriptomic data from mouse AD, T2D, AD × T2D with human data	Identifies estrogen and inflammatory pathways, including *IL6*, *JAK*, and *STAT* signaling, as cross-species predictors of AD outcomes; uncovers sex-specific translation despite male-only mouse data	Limited sample sizes; only homologous gene pairs used; preclinical findings require experimental validation	*P*-value: 0.0047 (T2D PC2), 0.0042 (T2D PC3), 0.0130 (AD × T2D PC3, 100 × 5 fold CV)	325
Deep-learning classifier	Clinical: 634 ROS/MAP donors with AD or control diagnosis; validated on 266 Mayo and 214 MSBB samples across six brain regions	Constructs pseudo-temporal trajectory for AD progression using RNA-seq; identifies 593 index genes grouped into four cell-type-specific modules linked to vascular, metabolic, neuronal, and glial dysfunction	Extend model to peripheral tissues and additional omics layers; improve interpretability and causal inference		326
PathFinder	Clinical: ScRNA-seq from *APOE4* astrocyte-specific AD mouse model (TAFE4_tam *vs*. TAFE4_oil); reveals *APOE4*-driven neuron–astrocyte–microglia signaling networks	Identifies intra- and inter-cell signaling alterations in autophagy, lipid metabolism, and JAK–STAT signaling; uncovers *MIF–EGFR* and *NLGN1–NRXN1* interactions linked to neuroinflammation and degeneration	Require larger multi-condition scRNA-seq datasets; enhance de novo discovery beyond predefined ligand-receptor databases	AUC: 0.73 (ex), 0.71 (mic), 0.65 (ast) F1: 0.68 (ex), 0.70 (mic), 0.69 (ast) Accuracy (5-fold CV): 0.67 (ex), 0.67 (mic), 0.62 (ast)	327
M3NetFlow	Clinical: 128 postmortem brain samples from ROS/MAP (64 AD, 64 non-AD); integrate transcriptomics, methylomics, proteomics, and CNV data to classify AD and identify novel biomarker signatures	Infers multi-hop, multi-pathway signaling networks using attention-based GNNs; identifies top-ranked AD-associated genes enriched in MAPK, JAK–STAT, PI3K–Akt, NF-*κ*B, and FoxO pathways	Require broader cohort validation; improve scalability and integration with ontology-guided pathway modules	Pearson correlation: 0.6734 ± 0.0512 on ROS/MAP (5-fold CV)	25
DIABLO	Clinical: 455 postmortem samples from ROS/MAP (307 MCI/AD, 148 controls); model integrates SNPs, RNA, CpGs, and proteomics to classify AD	Identifies 62 features across omics layers, including Tau peptide, SLC6A12 transcript, and GDF10 with strong cross-omics correlations; outperforms single-omics models with 90% Balanced Accuracy	Require validation in independent cohorts; incorporate additional omics and refine cross-layer biological network interpretation	Accuracy: 0.95; Balanced Accuracy: 0.90; Sensitivity: 0.96; Specificity: 0.83; F1: 0.95	328
JADBIO AutoML	Clinical: Public multi-omics blood datasets; 48 AD *vs*. 22 controls (miRNA), 134 AD *vs*. 100 controls (mRNA), 25 AD *vs*. 37 controls (proteomics); Identifies accurate, low-dimensional diagnostic signatures for blood-based AD screening	Constructs high-performing biosignatures using automated ML; miRNA signature with three predictors achieved best accuracy; supports cost-effective, minimally invasive diagnostic assays	Requires external validation across independent cohorts; needs integration with broader clinical and longitudinal datasets	AUC: 0.975 (miRNA, SVM), 0.846 (mRNA, RF), 0.921 (proteomics, Ridge LR)	329
ADAS-viewer	Clinical: Multi-omics data (RNA-seq, WGS, methylation, miRNA) from 7 brain regions across 3 AMP-AD cohorts (Mayo, ROS/MAP, MSBB); supports region-specific transcript and splicing analysis in AD *vs*. controls	Enables integrated exploration of transcript isoforms, eQTLs, sQTLs, methylation, and miRNA associations; reveals brain region-specific molecular alterations and links to Braak stage and dementia severity	Limited omics layers in certain regions; expansion and functional validation needed for broader applicability		330
GNNRAI	Clinical: 228 samples with both transcriptomics and proteomics data from ROS/MAP; classifies AD *vs*. control using multi-omics from the dorsolateral prefrontal cortex	Leverages knowledge-graph-based GNNs to integrate transcriptomics and proteomics; identifies key biomarkers; reveals biodomain interactions including lipid–mitochondrial metabolism, synapse, and apoptosis	Requires integration of additional modalities; needs improved subgraph interpretability methods	Validation: outperformed MOGONET in 13/16 AD biodomains	314
WGCNA; gene-miRNA network	Clinical: 329 brain samples from GSE63063, spanning normal (104), MCI (80), and AD (145) stages; Identify stage-specific gene and miRNA signatures for potential early diagnosis	Construct co-expression modules and gene–miRNA bipartite networks across AD stages; proposed 9 novel genes and 6 novel miRNAs as candidate biomarkers not previously reported in AD studies	Require experimental and clinical validation; expand toward mechanistic dissection and therapeutic development		331
M-Lasso	Clinical: 262 ROS/MAP postmortem brain samples (115 CN, 67 MCI, 80 AD); integrated SNP, RNA-seq, and proteomics data to identify multi-omic subnetworks predictive of cognitive decline	Identifies a 276-feature functionally connected subnetwork including *APOE*, *EP300*, GRB2, and PIK3R1; reveals trans-omic cascade from SNP to protein *via* gene regulation; enriched in PI3K-Akt, MAPK, and insulin signaling pathways	Limited to subjects with complete multi-omics data; lacks integration with imaging; interpretability of less-connected features remains difficult	RMSE: 0.935; MAE: 0.725 (5-fold CV); Features selected: 650 (incl. 255 SNPs, 339 genes, 56 proteins)	332
LASSO	Clinical: 80 plasma samples from SAND cohort (40 CN, 20 MCI, 20 AD); integrated proteomics, metabolomics, lipidomics to stratify AD progression	Identifies top protein and metabolite biomarkers with AUC ≥0.85; reveals arginine, alanine, glutamate, pyruvate pathway disruptions in MCI/AD	Requires biomarker validation in independent cohorts; unclear isoform specificity for some proteins; further integration with imaging data recommended	AUC: 0.72 (protein model), 1.00 (base + biomarker), 0.86 (Hypoxanthine) Sensitivity: 0.90 (Hypoxanthine) Specificity: 0.80 (Hypoxanthine)	333
Integrative network modeling (WGS, RNA-seq, BN, MEGENA and EMUDRA)	Clinical: 364 MSBB postmortem brain samples; stratified by CDR (0–5), Braak stage, CERAD score, and amyloid plaque density; samples span normal, MCI, and AD stages	Identify *ATP6V1A* as a key neuronal driver *via* BN and MEGENA; observe downregulation across LOAD stages; validate in hiPSC-derived neurons and Drosophila; rescue neuronal phenotypes and restore *ATP6V1A* expression with NCH-51	Requires validation in mammalian models; lacks multi-omics integration at the single-cell level; the drug mechanism of NCH-51 remains unclear		251
WGCNA; SCENIC; PPI network proximity modeling	Clinical: 230 brain samples from PFC, CR, VC regions (129 AD, 101 CN) across GSE44768, GSE44770, GSE44771; Identify AIM (A*β*-induced neuroinflammation module) highly expressed in AD; ssGSEA score significantly higher in AD across regions	Identify AIM comprising 177 genes enriched in neuroinflammation and neuronal death; AIM score negatively correlated with neuron percentage (*R* = −0.81); associated with Mic1 microglia subtype; Identify 7 TF biomarkers and 20 potential repurposing drugs, including ponatinib, ibrutinib	Limited to transcriptomic integration; requires protein-level validation; the mechanisms of candidate drugs need experimental confirmation	PR-AUC: 0.943 (5-fold CV	334
TACA	Clinical: >1.1 million sc/snRNA-seq profiles across 26 datasets from brain samples (hippocampus, cortex, cerebellum); covers AD, MCI, and CN individuals	TACA creation, 1.1 million single-cell profiles, 1400 differential analyses, 12 interactive tools	Integrates more multi-omics layers; improves metadata completeness; validates predicted drugs in experimental models		335
Random forest	Clinical: RNA-seq data from 638 ROS/MAP postmortem samples used to predict Braak stage, CERAD score, COGDX, and cognitive status at death	Uses gene regulatory network features to predict clinical phenotypes, including Braak, CERAD, and COGDX with >60% accuracy	Requires broader validation across independent cohorts; model complexity from scRNA integration limits generalizability	Balanced Accuracy: 0.57 to 0.68 (AD gene prediction, 5 × 10 CV) Accuracy: >0.60 (phenotype prediction, top 0.05 ranked genes: COGDX, Braak, CERAD, DCFDX)	336
DRIAD	Clinical: AMP-AD postmortem RNA-seq data (MSBB and ROS/MAP); samples categorized by Braak stage into early (1–2), intermediate (3–4), and late (5–6); DRIAD trained predictors for early *vs*. late stages	Links drug-induced gene signatures to AD severity; screened 80 kinase inhibitors; identifies JAK, ULK, NEK targets and autophagy pathways	Requires *in vivo* validation; assesses BBB penetrance and toxicity; integrates more omics		337
MultiDCP	Clinical: 46 AD patients from AMP-AD; individualizes drug response predicted based on transcriptomic profiles; reveals 3 molecular subtypes with distinct pathway enrichment	Develops MultiDCP for dose- and context-specific prediction of chemical-induced gene expression and cell viability; applies to personalized AD drug repurposing; identifies candidate drugs reversing patient-specific AD signatures	Model currently limited to 978 landmark genes; lacks integration with proteomics and imaging data; requires experimental validation in patient-derived models	Pearson: 0.493 (gene expression, leave-new-cell-out); 0.803 (cell viability) Spearman: 0.486 (gene expression, leave-new-cell-out); 0.780 (cell viability) Precision@10: 0.496/0.476 (up/down) AUC: 0.697 (up), 0.707 (down); PR-AUC: 0.127 (up), 0.123 (down)	338
Bayesian network modeling; network proximity scoring	Clinical: 7.23 million individuals from MarketScan claims; pioglitazone cohort (*n* = 101,650) showed reduced AD risk (HR = 0.916, *P* = 0.005)	Identify 103 AD risk genes through Bayesian multi-omics inference; prioritize 130 repurposable drugs using proximity to disease modules; validate top drugs *in vitro* and population cohorts	Validate ARGs in biological assays; incorporate biomarker-confirmed AD cases for clinical precision		339
AlzGPS	Clinical: Integrated >100 multi-omics datasets across species and brain regions; drug-dataset associations curated from ∼1000 AD clinical trials; drug repurposing predictions supported by case studies; NP: Clinical evidence extracted for top candidate drugs from 292 studies	Curates DNA, RNA, protein, and metabolite profiles from AD patient and model datasets; prioritizes 3000 drugs using network proximity to AD modules; enables endophenotype-specific MOA network visualization	Expands with sequencing from underrepresented populations; improves PPI completeness; integrates allele-specific and longitudinal data		340
DRPS/C	Clinical: RNA-seq (*n* = 159), microarray (*n* = 108), and proteomic (*n* = 17) AD datasets from Synapse, ArrayExpress, and PRIDE; predicts 31 anti-AD drug candidates across transcriptomic and proteomic signatures	Constructs DGPSD using 61,019 drug-induced gene signatures from 1520 compounds; identifies four nervous system drugs targeting sodium channels and monoamine oxidase pathways	Requires validation of drug efficacy *in vivo* and clinical trials; expand DRPS/C framework to integrate additional omics layers and patient-level precision medicine	AUC for high DRPC class (GBM): 0.708	341
TRS	Clinical: GWAS from 27 studies, RNA-seq from 1699 brains, proteomics from 1188 brains; assesses 600 AMP-AD targets and 192 drug targets; NP: Included cognition-linked genetic traits	Ranks AD risk genes by integrating GWAS, RNA, and protein data; classifies targets into 19 domains, including synapse, metabolism, and immunity	Expands to imaging and metabolomics; applies to AD subtypes; refines domain-level resolution		342
Iterative WGCNA; Subtype clustering; Linear mixed model	Clinical: 1258 postmortem brain samples from ROS/MAP, Mayo, and MSBB; stratified AD cases into molecular subtypes by region; subtype B in ROS/MAP associated with protective variant rs1990620 in TMEM106B NP: No significant difference in Braak stage, CERAD score, MMSE, or cognitive decline across subtypes	Identify 68 transcriptomic submodules across six brain regions; uncover subtype-specific modules linked to immune suppression and synaptic function; reveal new genetic modifiers such as TMEM106B and RBM39	Validate subtype dynamics across disease course; integrate proteomic and metabolic data; apply to subtype-targeted therapy development		343
Perspective analytics	Clinical: 380 AD post-mortem brains (GSE84422), six molecular classes; NP: low/high dementia label	Learns non-linear gene interactions from small, high-dimensional data; outputs explainable driver-gene signatures that reveal AD heterogeneity beyond conventional analyses	The proprietary NetraAI limits reproducibility; future work focuses on therapeutic translation of its findings	Leave-out cross-validation accuracy ≥0.75 for each perspective model; no AUC, sensitivity or specificity reported	344
RF; LASSO; SVM	Clinical: 737 AD samples from 8 GEO datasets; Identify 3 metabolic subtypes; MCA associated with higher *APOE4* frequency, *β*-secretase, *γ*-secretase, NFTs, and Braak	Define metabolic AD subtypes with distinct immune and metabolic signatures; Identify 8 feature genes; validated with qRT-PCR and diagnostic nomogram	Validate gene expression in human cohorts; clarify inconsistent KCNC1 results; explore the mechanism of the metabolism-immunity link	AUC (training/testing): Nomogram: 0.788/0.770 *GFAP*: 0.729/0.708 *EZR*: 0.692/0.698 *COLEC12*: 0.656/0.677 *KIAA0513*: 0.652/0.692 *CYB5R3*: 0.698/0.575 *DARS*: 0.560/0.566 *KCNC1*: 0.558/0.566 *TST*: 0.557/0.584	345
ConvAE	Clinical: 1.6M patients from Mount Sinai Health System; stratifies 3 AD subgroups across early-onset, late-onset with cerebrovascular disease, and mild-to-moderate AD; NP: Subgroups defined by dementia severity and symptom patterns; NI: MRI and PET used for differential diagnosis in the early-onset group	Develops ConvAE to learn unsupervised patient representations from raw EHRs; reveals meaningful AD subtypes reflecting disease onset, progression, and comorbidities	Improves EHR data preprocessing; reduces noise and bias		346
Consensus clustering	Clinical: 108 cognitively normal adults from Knight ADRC; stratified into three proteomic clusters with distinct progression risk; NP: Longitudinal CDR and MMSE used to assess cognitive decline across clusters; NI: MRI cortical thickness, WMH volume, PET-PiB and PET-AV1451 used to assess amyloid and tau pathology	Identifies proteomic subtypes predictive of preclinical AD progression; cluster 1 enriched for *APOE4* and tau PET positivity; cluster 3 showed greater WMH burden and atrophy	Validates clusters in larger multi-ethnic cohorts; integrates metabolomics and transcriptomics; links clusters to treatment response		347
NMF clustering	Clinical: 425 A*β*^+^ individuals from EMIF-AD MBD and ADNI cohorts; identify 3 CSF proteomic subtypes; NP: MMSE and domain-specific tests; CDR-SB tracked progression; NI: Cortical atrophy in hippocampus, temporal cortex, and precuneus; posterior cingulate thinner in subtypes 2 and 3	Defines subtypes with hyperplasticity, immune activation, and BBB dysfunction profiles; subtype 2 had 2.5-fold higher risk of progression than subtype 1; subtypes differed in tau, APP, and inflammation-related proteins	Validates across diverse populations; integrates plasma markers; explores subtype-specific therapeutic strategies		348
Blood transcriptome-based module model	Clinical: 422 individuals in discovery and 64 in validation cohort; distinguish 214 AD from 208 NC, 26 AD from 38 NC; NP: Module scores correlate with MoCA NI: Associated with hippocampal and amygdala volume	Identifies 6 gene modules; enables high-accuracy AD classification and stratification beyond ATN biomarkers	Validates in larger cohorts; examines cross-ethnic generalizability; integrates with proteomic and longitudinal data	AUC (discovery/validation): 0.990/0.897 (M02) 0.983/0.927 (M03) 0.998/0.920 (M15) 0.998/0.920 (combined model)	349
PCA; bicluster detection	Clinical: 2739 AD *vs*. 5478 NC (UKB); 500 AD *vs*. 470 NC (ADNI); NP: Bicluster 2 linked to faster PACC decline (*β* = −0.60, *P* = 0.004); NI: Bicluster 2 linked to increased CSF p-tau (*β* = 0.45, *P* = 0.041)	Identify replicable genetic subtypes; bicluster 2 shows aggressive progression	Interpret non-coding SNPs; validate in diverse populations; assess functional impacts		350
sPLS-DA	Clinical: 94 asymptomatic individuals; 48 amyloid+, 46 amyloid−; NI: Amyloid PET SUVr; correlated with CSF A*β*_42_ and p-tau	Identifies blood-based signature (MCFA, 4-nitrophenol, 64 transcripts); achieves 99.4% prediction for amyloid positivity	Small sample size; limited generalizability; no cognitive follow-up	AUC: 0.978 (metabolomics), 0.881 (transcriptomics), 0.732 (lipidomics), 0.994 (metabolomics + transcriptomics), 0.978 (metabolomics + lipidomics), 0.866 (transcriptomics + lipidomics)	351
Similarity network fusion; Spectral clustering	Clinical: 1313 ROS/MAP participants; 513 with 3-modal data; subtypes associated with cognitive decline; NP: Subtype 5 showed fastest decline in global cognition (*P* = 5.9 × 10^−3^)	Identify ageing subtypes integrating RNAseq, DNA methylation, histone acetylation; subtype 5 linked to poor cognitive outcomes	Optimal cluster number inconsistent across metrics; limited diversity; no standard cognitive biomarkers used		352
Integrative single-nucleus multi-omics	Clinical: 24 individuals (12 LOAD, 12 control, *APOE ε3/ε3*); 209,518 nuclei (RNA), 79,771 nuclei (ATAC), temporal cortex	Defines 518 LOAD-associated CCANs across 15 cell types; identifies cCRE-linked DEGs and TFs; prioritizes 20 regulatory SNPs	ATAC–RNA cluster-matching accuracy reported as 75.6%; no functional validation of regulatory SNPs; limited generalizability across brain regions		319
sc2GWAS	Clinical: 784 GWAS traits (∼5000 studies); 149 scRNA-seq datasets from ∼900 donors; ∼6.3 million cells analyzed; AD case study based on GSE207629 (brain immune cells)	Enables trait–cell mapping by integrating GWAS and single-cell data; identifies AD-enriched cell types and driver genes	Limited to scRNA-seq; lacks proteomic/epigenomic integration; trait–cell scores depend on GWAS and cell-type resolution		261
MOSEGCN	Clinical: ROS/MAP (AD: 182, Control: 169); BRCA (*n* = 875, 5 subtypes); GBM (*n* = 274, 4 subtypes); all with mRNA, miRNA, and DNA methylation	Demonstrates robust multi-omics classification across AD and cancers; identifies AD-related biomarkers	Lacks clinical/longitudinal validation; omics types are limited; no imaging integration	ROS/MAP: Accuracy: 0.8300; AUC: 0.832; F1: 0.827	262
MINDSETS	Clinical: ANMerge dataset; 1702 participants including AD (612), MCI (425), CTL (425), VaD (240); used longitudinal MRI, genotype, clinical, and MMSE data; NP: MMSE scores included (AD: mean 20.8, VaD: 22.6, MCI: 27.1, CTL: 29.1); NI: Longitudinal MRI scans at 0, 3, 12 months; segmented into 32 brain regions using SynthSeg and PyRadiomics	Achieves 89.25% accuracy in AD *vs*. VaD classification; model interpretable *via* SHAP; captures treatment response in MCI with longitudinal monitoring	Model not validated on external cohorts; longitudinal data introduces variability; requires tuning for other populations	Accuracy: 0.8925; AUC: 0.8361; F1: 0.8113	255
MSBB multi-omics platform	Clinical: 364 postmortem brain donors (Control, MCI, AD); Braak score: 2.1–5.1; CDR: 0–5; sex: 238 female, 126 male; AOD: 61–108 (mean: 84.7 ± 9.7); ethnicities: European (*n* = 301), African American (*n* = 36), Latino (*n* = 25), Asian (*n* = 1); NI: Neuropathological imaging data (plaque density, Braak staging) across 4 brain regions	Provides matched WGS, WES, RNA-seq (from BM10, BM22, BM36, BM44), and proteomics (BM10); highly curated clinical metadata; key resource for AD network modeling	Not model-based; no prediction task; requires external modeling; RNA–protein correlation low; batch effects in proteomics require correction		353
Federated LSTM with FedAvg	Clinical: 44,899 MCI patients; 6391 converted to AD; data from OneFlorida+ (6 simulated sites: North/Central/South Florida, Alabama, Georgia, Other); subgroup analysis by 6 m, 12 m, 24 m, unrestricted cohorts	Demonstrates the feasibility of federated learning for AD risk prediction across real-world, privacy-constrained settings; identifies key features (BMI, vitamin B12, BP, HDL, cholesterol) *via* SHAP	Excludes imaging/notes; EHR data may be incomplete; diagnostic miscoding possible; limited generalizability beyond southeastern U.S.	AUC (personalized): 0.794 (North FL), 0.744 (South FL); AUC (global): 0.837 (South FL)	263
Tri-COAT	Clinical: ADNI cohort; total *n* = 494 (slow: 177, intermediate: 302, fast: 15); grouped by 2-year MMSE change using baseline imaging, SNPs, and 7 cognitive scores; NI: Baseline T1-weighted MRI segmented into 72 ROIs	Enables early subtype prediction using baseline-only data; interpretable attention linking CD2AP–TRABSCOR–temporal gyrus; distinguishes fast progression subtype	Limited generalizability beyond ADNI; small fast-subtype sample (*n* = 15); no PET or transcriptomic integration	AUROC: 0.734 ± 0.076	264
SEA-AD atlas	Clinical: 84 postmortem donors (age: 65–108, mean 88; 51 female, 33 male); stratified by ADNC (9 no AD, 12 low, 21 intermediate, 42 high); 31/42 high-ADNC donors had dementia; NP: Longitudinal cognitive testing across 4 domains; memory decline slope: −0.15 in severely affected *vs* −0.11 in others	Integrates snRNA-seq, snATAC-seq, snMultiome, and MERFISH; maps 3.4 million nuclei to 139 supertypes; defines pseudoprogression score (CPS) to track disease trajectory	Not AI-based; no predictive model; analysis limited to MTG and A9 regions; lacks PET/MRI integration		354
MADDi	Clinical: ADNI cohort; 239 participants with all 3 modalities (CN: 165, MCI: 39, AD: 35); NP: 29 clinical features (e.g., memory, executive function, language tests); F1 per class: AD = 100%, MCI = 76.66%, CN = 97.81%; NI: T1 MRI (3 slices per patient), standardized by ADNI; 551 total MRI scans used for training	Integrates structured clinical, genetic (SNP), and imaging features *via* cross-modal attention; interpretable attention layers highlight key modalities	Small sample size for MCI and AD in the overlap group; excludes PET, longitudinal data, or unstructured text; limited external validation	Accuracy: 0.9688 F1: 0.9141 Class-wise F1: 1.00 (AD)/0.7666 (MCI)/0.9781 (CN)	266
Federated GEIDI	Clinical: ADNI cohort, *n* = 697 (AD: 96; MCI: 366; CU: 235); imaging and blood samples matched within 5 months; NP: MMSE (AD: 21.8 ± 4.1; MCI: 28.0 ± 1.7; CU: 29.1 ± 1.2)	Integrates gene expression and imaging *via* genotype-specific stratification; identifies XRCC1, SEC14L2 as subgroup-specific markers	No patient-level prediction; limited to two ROIs; no external validation	Hypergeometric p: 0.00039 (*APOE*/*HIPP*), 0.017 (rs942439) TPR: up to 0.61 (SNP detection)	267
BIONIC	Clinical: ROS/MAP cohort (TMT proteomics, *n* = 400); ADNI cohort (MCI: 112; mild dementia: 132); stratified by MMSE and CDR; NP: MMSE ≥21 for early AD; CDR memory box score ≥0.5	Reveals stress-response-mediated A*β*–tau modulators (HSPA5, GPNMB^+^ microglia, GFAP^+^ astrocytes); cross-validated in ROS/MAP, MSBB, ACT, and ADNI cohorts	Needs mechanistic validation of GPNMB^+^ microglia; lacks individual-level prognostic capacity; no imaging or PET data		53
Hybrid multimodal DNN + SVR	Clinical: 506 NA-ADNI participants (MCI or mild AD); baseline CDR-SB, MMSE ≥24, A*β*^+^ (CSF or PET); follow-up 2 years	Predicts individual cognitive decline (CDR-SB) and enables AI-driven stratified randomization; reduces allocation bias by 22%, trial sample size by 37%	Higher error in outliers; limited to CDR-SB endpoint; requires validation in real trials with other endpoints	MAE: 1.07; Corr: 0.58	268
NeuroPM-MCM + pTIF	Clinical: ADNI cohort (*n* = 504); each with 4–6 imaging modalities and ≥4 timepoints; longitudinal span = 5.1 ± 3.6 years	Predicts causal propagation of tau/amyloid/glucose alterations *via* brain networks; pTIF derives patient-specific therapy needs from model inversion	Requires high-quality longitudinal imaging; no plasma/PET-independent validation yet	*R*^2^: 0.92 (first TP), 0.71 (last TP) for 6-modal MCM simulation; *P* < 10^−6^	269
10-protein CSF model (LASSO)	Clinical: *n* = 2286 across Knight ADRC, FACE, ADNI, Stanford ADRC; 1254 AD, 750 controls; external validation in Stanford ADRC (*n* = 107); NI: Amyloid PET (*n* = 1127), Tau PET (*n* = 598); model predicts PET+ and AD status	Identifies robust, AT-stratified CSF proteomic signatures; model predicts A+T+, clinical AD, PET+, and progression to AD; replicable across platforms and cohorts	Requires CSF; plasma model needs re-training; lower performance in non-AD dementia (AUC 0.49–0.67)	AUC: >0.98 (biomarker); 0.89 to 0.97 (clinical); 0.90 (PET) OR: 2.03 (95% CI: 1.03–4.32)	270
EMIF-AD MBD cohort	Clinical: 1221 individuals (NC: 492; MCI: 527; AD-type dementia: 202); age: 67.9 ± 8.3; follow-up (mean: 2.3 years) in 758 individuals	Multicenter harmonized database with CSF, plasma, MRI, GWAS, methylation, and metabolomics; enables endophenotype-based biomarker discovery	Heterogeneous data acquisition; partial missingness across modalities; no direct prediction model		355
Graph diffusion-based AD gene network	Clinical: Gene expression datasets (microarray GSE1297, scRNA-seq GDS1979, snRNA-seq AD00204); no individual diagnosis; NI: Not used; analysis based on regulatory topology of 2363 AD genes in an 11K-gene consensus network	Constructs a robust AD-specific network from 15 datasets using multi-layer graph diffusion; reveals hub/periphery AD gene functions; identifies PON3 as an influential peripheral AD gene	No subject-level prediction; lacks clinical/diagnostic labels; gene-level inference only		226
Multi-omics mediation model	Clinical: 166 participants (HC: 61, MCI: 75, AD: 30); age: 50–85; MoCA + MMSE + AVLT; AD diagnosed per NIA-AA and DSM-IV; NP: MMSE (HC: 28.7, MCI: 25.8, AD: 14.7); AVLT 4-domain scores (all *P* < 0.001)	First integrative model to define microbiota → metabolites → brain → cognition mediation pathways in AD; reveals progressive alterations in 10 microbial taxa, 26 metabolites, and 74 GM/WM regions	Cross-sectional; no AI/predictive modeling; diagnosis lacks PET or fluid biomarkers; not externally validated		356
MFDDA	Clinical: ADNI cohort, *n* = 1171 (HC, EMCI, LMCI, LOAD); analyzed 7700+ multimodal brain images + 146 plasma & 87 CSF markers; NP: MMSE used to define symptom severity; stratified into 4 states (HC–EMCI–LMCI–LOAD); NI: A*β* PET, FDG-PET, ASL MRI, structural MRI, resting-state fMRI; 78 grey matter regions assessed	Proposes generative spatiotemporal trajectories of biomarker abnormality; finds vascular dysregulation precedes A*β*, metabolism, function, atrophy; identifies early plasma biomarkers (IP-10, PAPP-A, hFABP)	Does not build predictive models; not validated on external cohorts; linearity assumption may oversimplify complex interactions		357
Ceramide-based prognostic model	Clinical: 63 participants with baseline blood; NC (*n* = 25), MCI (*n* = 17), AD (*n* = 21); 71 completed 1-year follow-up; NP: MMSE (NC: 28.8, MCI: 27.2, AD: 22.2); detailed battery: ADAS-Cog, CVLT, WMS, Trails A/B	Plasma ceramides C22:0 and C24:0 predict 1-year cognitive decline and hippocampal volume loss in MCI; an early, non-invasive blood-based biomarker of AD progression	Limited sample size; unclear laterality mechanism (right hippocampus only); no model generalizability across cohorts		358
Colocalization + mediation analysis	Clinical: UK Biobank imaging-genetics cohort, *n* = 38,195; AD-by-proxy: 9788, controls: 28,407; NI: T1 MRI-derived volumetric endophenotypes from 145 brain ROIs (MUSE), especially entorhinal cortex	Discovery SNP rs6585827 upregulates BTBD16 in oligodendrocytes, reduces atrophy in the entorhinal cortex, and protects against AD	Lacks patient-level prediction; findings rely on AD-by-proxy; no deep learning integration		386
RF with SHAP	Clinical: 810 unique participants; OASIS-3; 5 labels (CN: 2248, AD: 669, Non-AD: 106, Uncertain: 287, Others: 26); NP: MMSE, Logimem, Digit Span, Boston, WAIS, Trail A/B	First five-class explainable ML model integrating clinical, cognitive, and structural MRI data; SHAP identifies Memory, Sumbox, Judgment, etc., as top features	Class imbalance (solved by SMOTE); not externally validated; shallow models used (no DL)	Accuracy: 0.9881; Precision: 0.9894; Recall: 0.9879; F1: 0.9875; AUC: 0.9997	271
ChatGPT-driven drug repurposing hypothesis	Clinical: VUMC and All of Us cohorts; 2 EHR datasets totaling ∼3.2 M patients; 10 drugs tested in matched cohorts age ≥65; NP: AD diagnosed using ICD codes (PPV 94%); AD onset tracked for 10 years	ChatGPT-generated drug list validates in real-world data; metformin, simvastatin, and losartan are associated with reduced AD risk (HR: 0.67, 0.84, 0.76); meta-analysis supported the top 3 drugs	No mechanism exploration; ChatGPT prioritization based on literature frequency, not biological evidence; no new AI training	HR: 0.67 (metformin, *P* = 6.4 × 10^−5^); 0.84 (simvastatin, *P* = 0.024); 0.76 (losartan, *P* = 0.017)	360
Transformer-based multimodal model	Clinical: 51,269 participants across 9 cohorts (ADNI, NACC, FHS, etc.); test: 12,950; predicts NC, MCI, DE, 10+ dementia etiologies; NI: T1/T2/FLAIR/SWI/DWI MRI; PET (A*β*, tau, FDG); DaTscan; supports inference with partial imaging	Enables accurate differential diagnosis of mixed/multi-etiology dementia using incomplete data; interpretable *via* SHAP and aligned with postmortem/pathology	Lower AUPR in rare conditions; modest generalization on FHS data; no staging (CDR levels merged); class imbalance	AUROC: 0.96 (micro), 0.94 (weighted); AUPR: 0.70 (micro); AI-augmented neurologist AUROC: 0.2625, AUPR: 0.7323	272
Multiomics clustering and subtyping using ML	Clinical: ADNI cohort (*n* = 377 for structural MRI; *n* = 130 for fMRI); includes cognitively normal, MCI, and AD dementia patients	Identify consistent anatomical and functional subtypes across datasets; shows alignment with known clinical variants; potential to define predictive multiomics biotypes for stratified medicine	Lack of standardization in subtyping; subtypes often overlap; limited external validation; clinical translation depends on integration with biomarkers like PRS and metabolite panels		361
Joint model for longitudinal + survival data	Clinical: 384 MCI patients from ADNI-1; mean age: 74.7; MMSE: 24–30; *APOE4*^+^ 54.2%; 200 converted to AD during 2.3 years mean follow-up; remaining 184 non-converters.; NP: ADAS-Cog13, RAVLT, MMSE, CDR-SB, MoCA.; NI: FDG-PET, MRI volumetrics (hippocampus, middle temporal gyrus, entorhinal cortex)	Joint modeling approach incorporates time-varying markers with survival data; ADAS-Cog13 identify as the strongest longitudinal predictor of AD conversion; cognitive markers outperform imaging in discrimination power over time	Imaging markers (FDG-PET, MRI) show lower discriminative power in later stages; CSF data excluded due to limited availability; joint model supports only a single longitudinal marker at a time; external validation needed	HR: 2.92 (ADAS-Cog13, 95 % CI 2.33 to 3.66); 3.16 (RAVLT); 1.95 (FAQ); 2.11 (FDG-PET) AUC: 0.74 to 0.86 (ADAS-Cog13); 0.76 to 0.85 (RAVLT) DDI: 0.79 (ADAS-Cog13, 6 to 12 months)	362
VBpMKL + MCI-CPS	Clinical: ADNI-1 (*n* = 125) and ADNI-GO/2 (*n* = 98) MCI patients; 3-year conversion prediction to AD; subtype-specific modeling; NI: 103 sMRI ROI volumes	Incorporate molecular subtype classification to enhance prediction; VBpMKL handles multimodal data (SNP, mRNA, sMRI); outperforms SVM, RF, logistic regression	Limited generalizability due to small sample size; requires validation on larger datasets; interpretability of kernel weights is still challenging	AUC: 0.8581 (subtype I), 0.8623 (subtype II), 0.83/0.78 (overall, internal/external) Accuracy: 0.7920 Sensitivity: 0.8125 Specificity: 0.7792	273
CNN + LRP	Clinical: 193 AD patients and 151 healthy controls from ADNI; 969 structural MRI scans; diagnosis based on ADNI clinical criteria; NI: relevance maps	First to apply LRP to 3D CNNs for MRI-based AD classification; generates individual heatmaps indicating decision-relevant brain regions; relevance scores correlated with hippocampal volume	Model trained only on AD *vs* HC; generalizability to MCI or early-stage detection not assessed; dependence on model quality for LRP interpretability; no cross-modal validation	Test accuracy: 0.8796; Validation accuracy: 0.9100; Correlation between LRP relevance and hippocampal volume: *ρ* = −0.560, *P* < 10^−3^	363
Non-visual XAI approaches	Clinical: describes applications on modalities such as MRI; X-ray; CT in secondary examples	Provide a structured taxonomy of non-visual XAI techniques in medical imaging, including case-based, textual, and auxiliary methods; integrate uncertainty estimation and model transparency into clinical workflows	Lack of clinical validation and integration studies; subjective perception of explanation quality; absence of standardized evaluation protocols; challenges in selecting appropriate XAI methods per clinical use-case		364
MCAD	Clinical: Diagnosis of AD, MCI, and CN using 467 multi-modal (sMRI, FDG-PET, CSF) data from 364 subjects from ADNI-1 and ADNI-2 datasets; NP: CN (*n* = 110, 156 scans), MCI (*n* = 125, 154 scans), AD (*n* = 129, 157 scans); age, MMSE, CDR-SOB mean (SD) reported; NI: sMRI and FDG-PET scans	Novel framework leveraging cross-attention for efficient and accurate AD diagnosis by learning inter-modality interactions; employs objective function (modality alignment loss, classification loss) to reduce heterogeneity	Existing models lack cross-modal interaction and ignore modality variations; challenges include effectively capturing inter-modality interaction and reducing modality discrepancy	AD *vs*. CN: ACC: 0.9107 ± 0.0382 SEN: 0.9103 ± 0.0478 SPE: 0.9107 ± 0.0647 F1: 0.9111 ± 0.0381 AUC: 0.9407 ± 0.0316; AD *vs*. MCI *vs*. CN: ACC: 0.6403 ± 0.0249 SEN: 0.6385 ± 0.0241 SPE: 0.8200 ± 0.0124 F1: 0.6185 ± 0.0158 AUC: 0.7677 ± 0.0422	274
Patch-based CNN	Clinical: Uses ADNI dataset to classify AD, MCI, and CN subjects using T1-weighted MRI and PET; regions such as the hippocampus and entorhinal cortex are identified as key contributors *via* Grad-CAM	Develops an interpretable CNN model that classifies AD by analyzing image patches and highlights disease-relevant ROIs	Limited to neuroimaging modalities; omics and clinical data not integrated		289

Note: A*β*, amyloid beta; AD, Alzheimer's disease; ADAS-Cog, Alzheimer's Disease Assessment – Scale Cognitive Subscale; ADNI, Alzheimer's Disease Neuroimaging Initiative; AE, autoencoder; AIM, A*β* induced neuroinflammation module; AMP-AD, Accelerating Medicines Partnership – Alzheimer's Disease; *APOE*, apolipoprotein E; APP, amyloid precursor protein; ATN, Amyloid, Tau, and Neurodegeneration; AUC, area under the ROC curve; AVLT, Auditory Verbal Learning Test; BBB, blood–brain barrier; BN, Bayesian probabilistic causal Network; BNT, Boston naming test; CDR, Clinical and Dementia Rating; CDR-SB, Clinical and Dementia Rating–Sum of Boxes; CN, cognitively normal; CNV, copy-number variation; COGDX, cognitive status at the time of death; CR, cerebellum; CSF, cerebrospinal fluid; CV, cross-validation; CVVLT, Chinese version verbal learning test; DBNs, Deep belief networks; DEG, differentially expressed gene; DIABLO, Data Integration Analysis for Biomarker discovery using the Latent cOmponents; DLPFC, dorsolateral prefrontal cortex; DMPs, differentially methylated positions; DRIAD, drug repurposing in Alzheimer's disease; EHRs, electronic health records; EMUDRA, Ensemble of Multiple Drug Repositioning Approaches; eQTL, expression quantitative trait loci; FAQ, functional activities questionnaire; FedAvg, federated averaging; fMRI, functional magnetic resonance imaging; GBM, glioblastoma multiforme; GCNs, graph convolutional networks; GEO, Gene Expression Omnibus; GNNRAI, GNN-derived representation alignment and integration; GWAS, genome-wide association studies; HDL, high-density lipoprotein; hiPSC, human induced pluripotent stem cell; HR, hazard ratio; IL, Interleukin; iPSNs, induced pluripotent stem-cell-derived neurons; JADBIO, Just Add Data Bio; LASSO, least absolute shrinkage and selection operator; LR, logistic regression; LRP, layer-wise relevance propagation; MAE, mean absolute error; MAPK, mitogen-activated protein kinase; MCI, mild cognitive impairment; MEGENA, multiscale embedded gene coexpression network analysis; MLP, multilayer perceptron; MMSE, Mini-Mental State Examination; MoCA, Montreal Cognitive Assessment; NP, Neuropsychological; NI, Neuroimaging; NFTs, neurofibrillary tangles; NMF, non-negative matrix factorization; S-MCI, stable MCI; P-MCI, progressive MCI; PCA, principal component analysis; PFI, permutation feature importance; PI3K, phosphoinositide 3-kinase; PPI, protein–protein interaction; PR-AUC, precision-recall area under curve; RF, random forests; RNA-Seq, RNA sequencing; RMSE, root mean square error; ROI, region of interest; ROS/MAP, Religious Orders Study and Rush Memory and Aging Project; scRNA-Seq, single-cell RNA sequencing; SGUQ, staged graph convolutional network with uncertainty quantification; SHAP, SHapley Additive exPlanations; SMOTE, synthetic minority over-sampling technique; SNPs, single-nucleotide polymorphisms; ssGSEA, single-sample gene set enrichment analysis; SUVR, standardized uptake value ratio; SVM, support vector machine; TACA, The Alzheimer's Cell Atlas; TF, transcription factor; TMT, tandem-mass-tag; TMT-B, trail making test part B; VF, verbal fluency; WGCNA, Weighted Gene Co-expression Network Analysis; WMH, white matter hyperintensities; XAI, explainable AI; AIBL, Australian Imaging Biomarkers and Lifestyle; PRS, polygenic risk scores; LSTM, long short-term memory; WES, whole-exome sequencing; WGS, whole-genome sequencing; PiB, Pittsburgh compound B; PET, positron emission tomography; p-tau, phosphorylated tau; ATP6V1 A, ATPase catalytic subunit A; *TREM2*, triggering receptor expressed on myeloid cells 2; GNNs, graph neural networks; sMRI, structural magnetic resonance imaging; WMS, Wechsler memory scale; WMS-LM, Wechsler memory scale-logical memory; TY-CFT, Taylor complex figure test; DGPSD, Drug-induced Gene Perturbation Signature Database; TRS, target risk score; PACC, Preclinical Alzheimer's Cognitive Composite; MCFA, medium-chain fatty acids; CCANs, cis co-accessibility networks; cCRE, candidate cis regulatory element; TPR, true-positive rate; GEIDI, Genotype-Expression-Imaging Data Integration; ATAC, assay for transposase-accessible chromatin; *SNCA*, alpha-synuclein; MOSEGCN, Multi-Omics Semi-Supervised Edge-to-Graph Convolutional Network; MINDSETS, Multi-omics Integration with Neuroimaging for Dementia Subtyping and Effective Temporal Study; UKB, UK Biobank; CNN, Convolutional Neural Network.

### 5.1 Early diagnosis and prediction

AD progresses insidiously through a prolonged preclinical phase, during which neuropathological changes accumulate in the absence of overt cognitive decline^80^^,^^365^^,^^366^. Identifying reliable and minimally invasive biomarkers during this asymptomatic window is critical for timely intervention. Recent advances in AI-driven multi-omics analysis have shown substantial promise in identifying novel preclinical biomarkers and constructing risk prediction models that outperform traditional single-omics or clinically based strategies^137^^,^^367^. Emerging approaches now integrate multi-omic layers with enriched clinical contexts, enabling more precise and individualized risk forecasting^124^^,^^159^^,^^368^.

Early studies employed algorithms such as random forests and support vector machines on modestly sized multi-omics datasets^369^, ^370^, ^371^. While these methods offered computational efficiency and interpretability, they often required extensive manual feature selection and were limited in capturing complex inter-omic interactions. The advent of deep learning models, including autoencoders, GNNs, and DBNs, has enabled direct learning from high-dimensional poly-omic matrices. These models have uncovered plasma proteomic and metabolomic signatures associated with amyloid burden, tau pathology, and cognitive resilience in presymptomatic individuals^372^, ^373^, ^374^. For instance, sliding-window association testing identified the lipid Cer(d16:1/22:0) as a significant feature, yielding a classification accuracy of 80.8% and an area under the ROC curve (AUC) of 0.808^375^. The B-HEALED model, which integrates blood-based multi-omics data, achieved 93.0% specificity in predicting prodromal AD, surpassing traditional amyloid-based diagnostic approaches^320^. DBNs have also proven effective in addressing the high-dimensional, low-sample-size challenge inherent to multi-omics datasets, demonstrating superior performance in feature selection and classification compared to conventional methods such as SVM-RFE^237^. Complementary approaches such as CE-GAN, which integrates fMRI and SNP data, have achieved over 91% accuracy in risk prediction tasks while maintaining interpretability^287^. Notably, the GNNRAI framework combines transcriptomic and proteomic graphs from the ROS/MAP cohort, employing a set-transformer for modality alignment. This model achieved a mean cross-validation accuracy of 0.83 across sixteen AD-relevant biological domains and outperformed the widely used MOGONET pipeline in external validation. SHAP-based interpretation further revealed lipid metabolism and synaptic signaling as key discriminative pathways, underscoring the model's biological relevance^228^.

Modern AI frameworks increasingly integrate multi-omics datasets with demographic, cognitive, and neuroimaging features to enable comprehensive cross-domain feature representation. Advanced architectures such as transformer-based encoders, variational autoencoders, and Similarity Network Fusion (SNF) frameworks are applied to jointly embed heterogeneous data sources. These sources include clinical assessments such as the Mini-Mental State Examination (MMSE), Clinical Dementia Rating–Sum of Boxes (CDR-SB), and the Alzheimer's Disease Assessment Scale–Cognitive Subscale (ADAS-Cog), along with neuroimaging markers including hippocampal volume, cortical thickness, and standardized uptake value ratios from amyloid and tau positron emission tomography imaging. When combined with transcriptomic, proteomic, metabolomic, and epigenomic layers, these models allow the identification of complex interdependencies across biological, cognitive, and structural domains that contribute to disease progression^262^^,^^352^. The MINDSETS model exemplifies this trend by integrating radiomic features from longitudinal MRI scans with polygenic risk scores, proteomics, and clinical assessments, achieving 89% diagnostic accuracy in distinguishing AD from vascular dementia^255^. Additionally, multimodal attention networks such as Tri-COAT demonstrate that hippocampal atrophy modulates the predictive contribution of lipidomic and genomic features, bridging molecular changes with regional neurodegeneration^264^. Temporal models employing convolutional or recurrent layers to align longitudinal neuropsychological trajectories with repeated blood-omics data have shown strong performance in individualized risk estimation. A recent long short-term memory (LSTM) model that fused demographics, serial cognitive assessments, and MRI data achieved an AUC of 0.93 ± 0.06 in predicting MCI-to-AD progression over two years^376^. Concurrently, federated learning has emerged as a privacy-preserving strategy for multi-center training. An LSTM model trained on 44,899 MCI cases across the OneFlorida+ network achieved improved generalizability, with AUC increasing from 0.72 in single-site models to 0.76 in the federated setting. This approach also identified clinically accessible predictors such as BMI, vitamin B12, and systolic blood pressure, highlighting its translational potential^263^.

Advanced computational pipelines now aim to integrate multi-omics with demographic, genetic, and lifestyle variables to improve the accuracy of individualized disease trajectory prediction^281^^,^^377^^,^^378^. While traditional models such as logistic regression and Cox proportional hazards provided initial insights, they are limited in handling high-dimensional, time-dependent data^379^. Contemporary methods, including recurrent neural networks and Bayesian deep learning, address these challenges by enabling dynamic risk estimation and uncertainty quantification. The B-HEALED platform, when combined with amyloid status and neuroimaging or CSF data, achieved 100% specificity for prodromal AD diagnosis^320^. Similarly, transcriptomic studies have identified concise gene panels with high diagnostic utility; for example, an eight-gene signature distinguished progressive from stable MCI with an AUC of 0.93^283^. Automated platforms such as JADBIO facilitate biomarker discovery by streamlining machine learning pipeline optimization and feature selection^329^. The WIMOAD framework integrates paired gene expression and DNA methylation profiles using stacked meta-learners, achieving an accuracy of 0.89 and AUC of 0.92 in cross-validation within the ADNI cohort. SHAP-based analysis highlighted ORMDL3 methylation and SLC1A2 expression as key features, providing experimentally testable insights and enhancing model interpretability^227^.

Despite these advances, key challenges remain. The reliance on large, high-quality datasets and the opaque nature of many AI models hinder widespread clinical adoption. Enhancing model transparency through methods such as attention mechanisms and layer-wise relevance propagation is crucial^380^. Additionally, validating models across diverse cohorts and harmonizing multi-omics data formats are necessary steps to ensure reproducibility. Emerging spatial proteomics technologies, such as DISCO-MS, offer the potential to create comprehensive multimodal atlases that link circulating biomarkers with regional neuropathology^381^. Overcoming these hurdles will be essential to fully realize the potential of integrated AI frameworks in the early detection and personalized prediction of AD.

### 5.2 Mechanism exploration

The multifactorial etiology of AD necessitates innovative analytical frameworks capable of disentangling its complex molecular architecture^128^^,^^156^. The application of state-of-the-art AI and machine learning algorithms has significantly enhanced our capacity to investigate AD pathogenesis. In particular, AI-driven multi-omics approaches are increasingly utilized to elucidate disease-associated mechanisms, evolving from early integrative pipelines into sophisticated deep learning frameworks^353^. These methodologies show great potential in advancing our understanding of AD by uncovering novel biomarkers, molecular pathways, and regulatory networks involved in disease initiation and progression.

Initial efforts employed classical machine learning models such as iClusterBayes to integrate transcriptomic, proteomic, metabolomic, and lipidomic datasets^382^. This integrative analysis revealed molecular subtypes of AD that correlated with neuropathological severity and cognitive decline, implicating key biological pathways including endocytosis and cholesterol metabolism^58^. Although these approaches successfully identified signatures associated with amyloid and tau pathologies, they often oversimplified the high-dimensional and non-linear relationships inherent to multi-omics data^383^. Additionally, conventional models such as random forests and support vector machines were limited in their ability to manage data variability, batch effects, and missing values^384^. Nonetheless, these foundational studies established a critical framework for examining multilayered molecular alterations in AD and set the stage for the adoption of more advanced AI techniques.

The transition to deep learning models has enabled more sophisticated analyses of complex biological data. Tools such as scGraph2Vec and NETTAG exemplify this shift. scGraph2Vec integrates single-cell RNA sequencing with gene interaction networks, while NETTAG combines deep learning with GWAS data to prioritize disease-relevant pathways and genes^265^^,^^323^. For example, NETTAG highlighted microglial gene networks and immune-related pathways, facilitating novel drug repurposing strategies, including the identification of gemfibrozil as a potential therapeutic agent^323^. Furthermore, autoencoder-based models such as EnsembleOmicsAE have been employed to reduce dimensionality in proteomic datasets, uncovering latent features linked to disease progression. This approach revealed cell adhesion and integrin signaling pathways not readily detected by linear models^324^. Recent integrative studies have expanded these insights using single-cell and spatial omics technologies. A large-scale analysis of 84 post-mortem AD brains employed single-nucleus RNA sequencing, spatial transcriptomics, and epigenomics to map cellular trajectories across an AD pseudoprogression continuum. Early disease stages were marked by inflammatory microglia, reactive astrocytes, and oligodendrocyte precursor-driven remyelination, while later stages exhibited vulnerability in excitatory neurons and VIP/PVALB interneurons^354^. These findings underscore the value of spatially resolved multi-omics in delineating cell-type-specific dynamics and identifying stage-specific therapeutic targets.

Building on omics-centric methodologies, next-generation AI frameworks integrate molecular data with neuroimaging and cognitive assessments to model mechanistic cascades from genetic variation to neurodegeneration. The MADDi cross-modal attention network exemplifies this paradigm by linking structural MRI, genome-wide SNP profiles, and longitudinal clinical data. Its attention maps demonstrate that hippocampal thinning amplifies the effect of inflammation-related SNP clusters on memory decline, suggesting an immune-mediated feedback mechanism^266^. In a federated learning context, the GEIDI framework connects genotype-dependent transcriptomic alterations to cortical thickness patterns, identifying synaptic vesicle genes implicated in temporal lobe atrophy and outperforming traditional eQTL-based inference models^267^. Complementary research in non-human primates has produced a macaque brain atlas integrating MRI-derived cortical thickness with bulk and single-nucleus transcriptomic data. This work revealed a mitochondrial oxidative phosphorylation module whose expression gradient mirrors prefrontal cortical thinning, highlighting metabolic vulnerability as a regional determinant of atrophy^359^. Collectively, these integrated models demonstrate how embedding multi-omics within neuroimaging and clinical contexts can reveal causal pathways linking molecular dysregulation to structural and cognitive decline. High-throughput integration of transcriptomic, proteomic, and metabolomic profiles from post-mortem AD brains has exposed molecular heterogeneity across cortical regions. Network-based modeling approaches have identified critical regulators of neuronal dysfunction, such as ATPase catalytic subunit A (ATP6V1A), whose therapeutic potential was validated *via* CRISPR-based experiments^251^. Single-cell omics further refines our understanding of cellular heterogeneity, showing that astrocytic dysregulation contributes to tau pathology and mitochondrial impairment^280^.

Epigenetic modifications have also emerged as key regulators in AD pathogenesis. Combined analyses of ChIP-seq and RNA-seq data have linked histone acetylation marks, including H3K27ac and H3K9ac, to transcriptional dysregulation affecting synaptic and chromatin-related genes^104^. Deep learning models such as BIONIC have facilitated mechanistic dissection by integrating ROS/MAP proteomic data with PPI networks. This analysis highlighted the roles of GPNMB-positive microglia and astrocytic markers like IBA1 and GFAP in mediating the amyloid–tau cascade. The development of interpretable network-based resources through such frameworks provides not only testable hypotheses but also strategic insights into cell-type-specific therapeutic targeting^53^.

Despite these promising developments, several challenges persist. The limited interpretability of deep learning models, inconsistencies in multimodal data acquisition protocols, and the computational intensity of large-scale analyses continue to impede broader application. Addressing these issues requires transparent AI models, federated-learning frameworks, and scalable cloud-native infrastructures^385^. Looking ahead, the convergence of single-cell and spatial multi-omics with ultra-high-field MRI and comprehensive cognitive testing is poised to enable fine-grained spatiotemporal dissection of neuroinflammation, tau aggregation, and metabolic disruption^386^. Cross-species modeling platforms such as TransComp-R further underscore the translational potential of aligning conserved molecular pathways between preclinical models and human patients, paving the way for more effective therapeutic strategies.

### 5.3 Novel biomarker identification

Advancements in AI-driven multi-omics analysis have significantly accelerated the identification of novel biomarkers for AD, facilitating a transition from reliance on single diagnostic indicators to the construction of comprehensive and dynamic disease signatures^387^. The emergence of multi-modal biomarker panels and dynamic biomarker profiling has proven instrumental in capturing the multifaceted nature of AD pathology, thereby enhancing early diagnosis and enabling precision-targeted therapeutic strategies^388^^,^^389^.

The concept of multi-modal biomarker panels arises from the understanding that AD is underpinned by complex molecular and cellular alterations spanning genomics, transcriptomics, proteomics, metabolomics, and neuroimaging^159^. The integration of these heterogeneous data modalities allows for the dissection of disease heterogeneity and the identification of robust biomarkers that reflect diverse pathological pathways. This integrative approach is particularly crucial in light of individual variability in molecular drivers, where single biomarkers often fail to represent the full complexity of disease mechanisms.

Platforms such as ADAS-Viewer exemplify the potential of spatially resolved omics analysis, enabling the identification of region-specific transcriptomic and epigenetic alterations associated with AD onset and progression^330^. These tools facilitate the mapping of vulnerable brain regions and the development of localized therapeutic interventions. Concurrently, AI methodologies such as graph neural networks leverage known biological interactions, including gene–gene and protein–protein relationships, to enhance biomarker discovery. These models not only improve predictive performance but also elucidate interconnected molecular subnetworks that mediate broader disease processes^314^^,^^332^.

Recent multimodal studies have demonstrated how peripheral molecular signatures can be linked to *in vivo* brain changes. For instance, machine learning models trained on baseline plasma ceramide species, including C22:0 and C24:0, successfully predicted one-year hippocampal atrophy in individuals with amnestic mild cognitive impairment, suggesting that blood-based lipidomic markers can anticipate neurodegenerative changes prior to clinical dementia^358^. Similarly, the federated GEIDI framework integrates transcriptomic and GWAS data with structural MRI, revealing genotype-dependent gene expression effects on cortical thickness and identifying synaptic vesicle-related genes implicated in temporal lobe atrophy^267^. At a broader systems level, integrative studies have combined large-scale brain imaging data with plasma and CSF proteomic profiles to generate dynamic abnormality indices. These models charted sequential disruptions in vascular function, amyloid deposition, and cognitive decline^357^. A separate analysis involving CSF proteomics across six international cohorts and over 2000 protein targets achieved an AUC exceeding 0.90 in predicting AD biomarker positivity, demonstrating applicability across both symptomatic and asymptomatic populations^270^. Furthermore, multimodal studies combining gut microbiome sequencing, fecal metabolomics, and neuroimaging have revealed bidirectional mediation pathways, establishing mechanistic links between gut dysbiosis, lipid metabolism, and progressive brain atrophy^356^. These findings underscore the value of integrating peripheral and central data streams to trace biological cascades underlying AD progression.

AI-based analyses have also uncovered molecular heterogeneity within the AD population. High-throughput transcriptomic and proteomic studies have delineated at least four molecular subtypes of AD, each characterized by distinct immune, metabolic, and inflammatory signatures that correlate with clinical trajectories^58^. This stratification has significant implications for risk assessment, trial design, and personalized treatment development. Deep multi-omics models such as MoFNet illustrate how integrating DNA, RNA, and protein expression reveals distinct pathways related to neurotransmission and immune regulation, thereby moving the field toward individualized mechanistic understanding^286^.

Static biomarker panels, although informative, often lack the temporal resolution necessary to capture the dynamic course of AD. AI-enabled time-series modeling and dynamic Bayesian inference now allow researchers to monitor longitudinal changes in biomarker levels. For example, modularity-constrained Lasso has been used to link genetic variants to transcriptomic and proteomic alterations that collectively influence cognitive function^332^. Integrated temporal analyses of transcriptomic and lipidomic data have further revealed co-expression modules associated with disease severity and progression^58^^,^^280^. Changes in tau phosphorylation and markers of synaptic dysfunction have emerged as reliable early indicators of neurodegeneration. Longitudinal transformer-based models aligning cognitive assessments with cerebrospinal tau levels and DTI have identified change-point patterns that signal imminent cognitive decline. Systems biology platforms like M3NetFlow map genetic variants to molecular pathways and phenotypic outcomes, identifying key regulatory nodes and optimal windows for intervention^25^.

Despite these advancements, the integration and interpretation of large-scale multi-omics datasets remain challenging. Data heterogeneity, cohort-specific biases, and missing values complicate consistent biomarker discovery. Although tools such as Weighted Gene Co-expression Network Analysis and Bayesian clustering provide partial solutions, more robust and scalable approaches are needed to manage the scale and complexity of these data^280^^,^^331^. Single-cell multi-omics has further refined biomarker discovery by enabling cell-type and cell-state-specific resolution, though this granularity increases the computational burden and necessitates improved harmonization protocols^58^. User-friendly platforms like ADAS-Viewer play a critical role in translating these complex analytical outputs into clinically actionable insights by promoting accessibility and collaborative analysis^330^.

In conclusion, AI-driven multi-omics approaches are reshaping the landscape of biomarker discovery in AD. By integrating diverse molecular, imaging, and cognitive domains, these methodologies facilitate earlier and more accurate diagnosis, support precision medicine efforts, and provide mechanistic insights essential for therapeutic development. Continued innovation in computational modeling, data harmonization, and platform accessibility will be pivotal in translating these discoveries into effective clinical interventions.

### 5.4 Drug discovery and development

The application of AI-driven multi-omics strategies is fundamentally transforming the landscape of therapeutic discovery in AD. By integrating genomic, transcriptomic, proteomic, and metabolomic data, these approaches offer a comprehensive perspective on the molecular complexity of the disease. This systems-level integration enables the identification of new therapeutic targets, supports the repurposing of existing drugs, and informs the development of treatments that align with disease-specific molecular mechanisms.

A number of computational frameworks exemplify this paradigm. DRPS/C leverages inverse gene and protein perturbation signatures to predict candidate anti-AD agents, such as bupivacaine and selegiline, both of which have demonstrated neuroprotective effects in preclinical models^341^. Similarly, NETTAG, a network-based deep learning platform, combines GWAS data with the human interactome to prioritize risk genes and identify compounds such as gemfibrozil that have been epidemiologically associated with reduced AD incidence^323^. Other network-centric models, including AlzGPS, map genetic variants onto protein interaction networks to identify modifiable disease nodes. One such node, ATP6V1A, was found to be pharmacologically targetable, with compounds like pioglitazone modulating its associated neuroinflammatory pathways^251^^,^^334^^,^^337^^,^^339^^,^^340^. The Alzheimer's Cell Atlas (TACA) further enhances cell-type-specific drug discovery by aggregating over one million single-cell profiles, facilitating targeted therapeutic development at unprecedented resolution^335^.

The integration of molecular data with clinical, cognitive, and neuroimaging information is now a hallmark of next-generation therapeutic discovery pipelines. The open-access NeuroPM-box platform exemplifies this approach by modeling disease progression using structural and functional MRI, amyloid and tau PET, blood and CSF proteomics, multi-omics genetic data, and longitudinal cognitive assessments^269^. In a proof-of-concept analysis using data from the ADNI, simulations predicted that vascular-modulating agents would most effectively slow hippocampal atrophy in *APOE ε4* carriers, a finding later corroborated by observational data. Similarly, the EMIF-AD Multimodal Biomarker Discovery study integrates plasma metabolomics, CSF proteomics, GWAS data, high-resolution MRI, and detailed clinical follow-up in over 1200 European participants. This comprehensive dataset has already identified blood-based lipid panels that parallel amyloid PET burden and predict cognitive decline, thereby providing pharmacodynamic readouts for future clinical trials^355^. In the context of trial design, a multimodal deep learning model was developed to integrate baseline MRI radiomics, plasma proteomics, and neuropsychological assessments for forecasting individual cognitive trajectories. When applied to simulated randomization schemes, this model enhanced the stratification of fast versus slow cognitive decliners, thereby reducing required sample sizes and increasing statistical power for detecting treatment effects^268^.

Deep learning platforms are also driving innovation in compound screening and combination therapy prediction. Tools such as DeepDrug and MultiDCP exemplify this trend. DeepDrug identifies synergistic therapeutic combinations targeting neuroinflammation and mitochondrial dysfunction^390^, while MultiDCP models dose-dependent gene expression changes, enabling the stratification of patient subgroups based on predicted molecular responses^338^. Although these models accelerate the drug development process, rigorous biological validation remains essential to mitigate potential off-target effects^390^^,^^391^. The Recursive Drug Repositioning Paradigm advances this effort by simulating drug-disease interactions and refining candidate compounds through iterative learning^391^. This strategy has facilitated the discovery of agents like NCH-51, which reverses ATP6V1A-associated neuronal dysfunction^251^. Emerging spatial proteomics technologies further enhance precision targeting. DISCO-MS, for example, maps molecular heterogeneity across three-dimensional brain structures, identifying early pathological signals critical for therapeutic validation^381^. Real-world initiatives, such as MEMORI-AD, integrate clinical, genetic, microbial, and metabolomic data to monitor treatment response, demonstrating the feasibility of multi-omics-informed, AI-driven therapeutic guidance in clinical settings^392^. The use of large language models in drug repurposing represents another promising frontier. A recent study employed GPT-4 to generate and prioritize potential AD therapeutics, followed by retrospective validation in two large-scale electronic health record cohorts from Vanderbilt University and the All of Us Research Program. Metformin, simvastatin, and losartan were each associated with reduced AD incidence after adjusting for confounders, highlighting the utility of generative models in hypothesis generation and prioritization^360^. Building on the original DeepDrug framework, DeepDrug2 addressed somatic mutation biases by reconstructing a signed, heterogeneous biomedical graph focused on germline GWAS associations. Training a graph neural network on this architecture enabled reprioritization of compounds with improved disease specificity. Analysis of 500,000 participants in the UK Biobank revealed significant protective associations for amlodipine, indapamide, and atorvastatin, illustrating how germline-informed embeddings and real-world clinical data can jointly support the identification of actionable therapeutic candidates^240^. Collectively, these developments underscore the transformative potential of AI-integrated multi-omics pipelines in accelerating therapeutic discovery and repurposing for AD. By uniting predictive modeling, mechanistic interpretability, and real-world validation, these frameworks pave the way for precision therapeutics that are biologically grounded and clinically actionable.

Nevertheless, several challenges persist. Data heterogeneity, limited model interpretability, and unequal access to computational infrastructure hinder widespread application. Collaborative efforts such as the Synapse repository and cross-disciplinary consortia are working to harmonize datasets, promote transparency, and align discovery efforts with regulatory frameworks^393^. Ethical considerations remain paramount, particularly concerning data privacy and the equitable distribution of emerging therapeutic innovations^394^. In summary, AI-integrated multi-omics methodologies are reshaping the drug discovery landscape for AD. These approaches facilitate systematic target identification and compound screening, offering a scalable and mechanistically informed path toward clinical translation. Realizing their full potential will require sustained investment in validation, interpretability, and regulatory alignment.

### 5.5 Patient stratification and personalized medicine

The convergence of AI and multi-omics technologies is transforming patient stratification and the development of personalized medicine in AD. Multi-omics profiling has revealed substantial molecular heterogeneity in AD, providing critical insights into individual variability and patterns of disease progression. Modern AI-based stratification frameworks now incorporate genetic information, proteomic signatures, and clinical progression metrics to refine subtype classification and guide individualized interventions.

Genetic factors are central to these frameworks. The *APOE ε4* allele remains the most prominent genetic risk factor, strongly linked to both disease susceptibility and progression^395^. In addition to *APOE*, polygenic risk scores (PRS), which aggregate multiple genetic variants across the genome, are increasingly integrated into AI models to capture complex hereditary risk profiles. These genetic variables are often combined with proteomic features that reflect pathological processes such as neuroinflammation, synaptic dysfunction, and mitochondrial impairment. For instance, protein expression patterns associated with microglial activation and synaptic vesicle transport have been employed to define biologically meaningful patient subgroups. Moreover, clinical phenotypes, such as longitudinal trajectories of cognitive decline, neuropsychological scores, and fluid biomarker dynamics, are incorporated to enhance the temporal resolution of stratification.

Large-scale studies such as ROS/MAP project have utilized multi-omic data from dorsolateral prefrontal cortex tissue to identify AD subtypes that exhibit distinct cognitive trajectories. These subtypes differ in lipid metabolism and synaptic function, highlighting the utility of combining molecular and clinical data to capture disease heterogeneity^124^^,^^255^^,^^396^^,^^397^. Complementary analyses of peripheral blood transcriptomes have enabled patient classification into immune and metabolic subgroups, achieving diagnostic performance comparable to established biomarker frameworks like Amyloid, Tau, and Neurodegeneration (ATN)^71^^,^^349^^,^^351^^,^^398^. Advanced integration techniques such as similarity network fusion allow for the joint analysis of transcriptomic, proteomic, DNA methylation, and metabolomic data derived from CSF, blood, and brain tissue. These analyses have identified subtypes characterized by immune activation, mitochondrial dysfunction, lipid dysregulation, and BBB disruption^342^^,^^352^^,^^399^^,^^400^. The addition of neuroimaging data further enriches stratification efforts by revealing distinct progression patterns across biomarker-defined subgroups^347^^,^^401^^,^^402^.

Recent multimodal AI pipelines build upon these findings by embedding omics features alongside neuroimaging trajectories and neuropsychological assessments. For example, the MINDSETS model generates individualized risk profiles and cognitive decline trajectories by linking multi-omic embeddings with longitudinal Mini-Mental State Examination scores^255^. Similarly, the MCAD framework integrates structural MRI, FDG-PET, and cerebrospinal biomarkers to classify patients into diagnostic categories, highlighting interactions such as the synergy between tau burden and regional hypometabolism^274^. Models like WIMOAD apply ensemble learning to integrate gene expression and DNA methylation data, aligning the resulting molecular profiles with cortical thickness maps derived from MRI, thereby identifying early versus slow progression phenotypes within the ADNI cohort^227^.

AI has further enhanced the scalability and interpretability of these frameworks. Explainable models using SHAP analysis on combined clinical, neuroimaging, and psychological inputs have achieved classification accuracies approaching 99% while clearly identifying key contributors such as memory test scores and hippocampal atrophy^271^. In the INSIGHT-preAD cohort, unsupervised learning revealed blood-based multi-omic signatures capable of predicting early amyloid deposition with over 99% accuracy^351^. Deep learning frameworks continue to uncover novel biological pathways, including those related to neuroinflammation and metabolic regulation, which account for diverse disease trajectories^397^^,^^398^. Interactive platforms employing machine learning have defined distinct AD subgroups characterized by pathways involving vasculogenesis and nitric oxide signaling^344^. Multi-kernel learning techniques have improved integration of high-dimensional, multimodal datasets, further enhancing performance and clinical applicability^403^. Importantly, AI-driven stratification is beginning to inform therapeutic decision-making by identifying molecular subtypes that respond differently to interventions. For instance, patients with dominant neuroinflammatory profiles may benefit from early immune-modulating treatments, while those with mitochondrial dysfunction could be targeted with metabolic therapies. Proteomic clustering has identified subgroups particularly responsive to drugs that modulate synaptic function and restore BBB integrity^342^^,^^345^^,^^347^^,^^350^^,^^352^^,^^399^^,^^400^. In one study, plasma inflammatory proteomics, untargeted metabolomics, and both gut and salivary metagenomics were integrated to classify cognitive severity in AD patients. The machine learning model identified SKAP1, NEFL, homovanillate, glutamate, and the gut microbe *Paraprevotella clara* as key predictors, demonstrating high accuracy in a validation cohort^234^. At an international scale, a consortium aggregated clinical and neuroimaging data from over 51,000 participants across nine cohorts to build an XAI framework capable of distinguishing among ten dementia etiologies. The model achieved an area under the receiver operating characteristic curve of 0.94 for classifying cognitive states and 0.96 for identifying co-existing pathologies. Importantly, the integration of model-derived insights improved neurologist diagnostic accuracy by 26%, demonstrating the utility of AI-assisted decision support in routine clinical care^272^.

Despite significant progress, several technical and practical challenges remain. Harmonization of multi-omics datasets is complicated by batch effects, inconsistent data quality, and cross-platform variability. Integration across data modalities can introduce biases, as seen in joint analyses of transcriptomic and epigenomic features, which may complicate interpretation^352^^,^^396^. Deep learning models, while effective, often lack transparency, which limits their clinical adoption. Addressing these limitations will require continued development of interpretable AI algorithms, standardized analytical workflows, and collaborative data-sharing platforms. Ensuring adequate representation of under-studied populations, as highlighted in the AMP-AD initiative, is also essential for building globally applicable stratification frameworks^351^^,^^397^.

## 6 Challenges and solutions

Despite substantial progress, the application of AI in multi-omics research for AD continues to face significant challenges spanning technical, biological, and clinical domains. From a technical perspective, issues such as data heterogeneity, batch effects, and limited access to high-performance computing resources hinder reproducibility, scalability, and cross-study generalization. On the biological front, the inherent complexity and inter-individual variability of AD pathology across different disease stages pose major obstacles to model development and biological interpretation. Clinically, factors including limited model transparency, infrastructural limitations, and regulatory constraints impede integration into routine healthcare settings. Overcoming these challenges will require harmonized data processing pipelines, biologically grounded patient stratification frameworks, rigorous cross-cohort validation strategies, and the development of XAI models that can be seamlessly embedded into clinical workflows.

### 6.1 Technical challenges

Integrating heterogeneous data across multiple omics layers, such as genomics, transcriptomics, proteomics, metabolomics, and epigenomics, remains a foundational challenge in AI-driven multi-omics research for AD^404^^,^^405^. These datasets are often generated through diverse experimental protocols and platforms, resulting in considerable variability in data quality, formats, scales, and batch-related artifacts^406^. Such heterogeneity introduces noise, missing values, and inconsistencies that undermine the robustness, reproducibility, and generalizability of AI models across cohorts and studies.

To address these issues, a range of preprocessing techniques has been adopted. Data normalization methods, such as log transformation, quantile normalization, and z-score scaling, are used to align distributional properties across datasets. Batch effect correction tools, including ComBat, Harmony, and Limma, are employed to harmonize data collected from different platforms. In addition, community-driven initiatives, including the FAIR principles (Findable, Accessible, Interoperable, and Reusable) and standardized data formats developed by consortia such as ENCODE and GTEx, have enhanced consistency, accessibility, and interoperability within the multi-omics research ecosystem.

Integrating high-dimensional omics data also imposes substantial computational demands. Algorithms such as SNF, Multi-Omics Factor Analysis (MOFA), and deep learning-based autoencoders are increasingly used to construct unified representations that preserve modality-specific information. However, these methods often require significant computing power and technical expertise, limiting their accessibility in resource-constrained research environments.

Reproducibility remains a major concern in AD multi-omics modeling. Studies typically involve tens of thousands of molecular features but relatively small sample sizes, often leading to overfitting and unstable feature selection. Power-aware study design is essential. For instance, MultiPower modeling has shown that approximately 500 paired omics and imaging samples are required to detect moderate molecular effects with a 5% false discovery rate in integrative analyses^407^. During model development, best practices include the use of nested or repeated k-fold cross-validation for hyperparameter tuning, the evaluation of performance metrics on strictly held-out datasets, and replication in independent cohorts such as ADNI or AddNeuroMed. Community benchmarking initiatives like the DREAM Alzheimer's Big Data Challenge and the TADPOLE forecast competition have underscored the necessity of rigorous external validation, revealing that many high-performing algorithms do not generalize well to new datasets^408^. Common methodological pitfalls further complicate reproducibility. Meta-analyses of machine learning applications across scientific domains have identified issues such as data leakage, inconsistent preprocessing, and lack of reporting transparency. These findings have prompted the development of reproducibility checklists and model information sheets to standardize reporting practices regarding data handling, model splits, and hyperparameter selection^409^.

Overfitting is a particularly pervasive issue in the analysis of high-dimensional omics data with limited sample sizes. Models often show high performance on training datasets but fail to generalize to independent cohorts, a pattern frequently reported in AD multi-omics studies^410^^,^^411^. Algorithmic bias can also emerge when training data do not adequately represent diverse populations. Systematic reviews of AI fairness in healthcare have demonstrated that models trained on demographically skewed datasets can produce reduced accuracy and exacerbate health disparities in underrepresented groups^412^. Another limitation lies in the interpretability of complex models. Many deep learning architectures operate as “black boxes”, providing limited insight into the rationale behind predictions. This lack of transparency impedes clinical adoption and limits the actionable value of AI outputs. XAI techniques offer promising solutions. SHAP values quantify the contribution of each feature to individual predictions and have been successfully applied in clinical decision-support systems ^413^. Layer-wise relevance propagation (LRP) generates feature-level heat maps that trace neural network decisions back through their layers, providing interpretability in MRI-based AD classifiers^363^. Attention mechanisms, as implemented in healthcare-specific models such as RETAIN, highlight critical visits and clinical variables in longitudinal electronic health record sequences, thereby offering predictive strength alongside human-readable explanations^414^^,^^415^.

In summary, resolving the technical challenges in AI-based multi-omics research for AD requires standardized preprocessing pipelines, power-aware validation strategies, transparent benchmarking practices, scalable computational infrastructures, and XAI frameworks. These advancements are essential for ensuring the robustness, generalizability, and clinical relevance of AI systems in AD research and beyond.

### 6.2 Biological challenges

AD is driven by a complex interplay of pathological mechanisms, including neuroinflammation, protein misfolding, mitochondrial dysfunction, synaptic degeneration, and disruptions in the gut–brain axis^416^. Multi-omics technologies offer a powerful means to investigate these molecular processes by integrating these datasets. However, these mechanisms are both temporally dynamic and spatially heterogeneous. The rates of disease progression, affected brain regions, and molecular cascades can vary significantly among individuals and across disease stages, posing significant challenges to the development of predictive models that accurately capture the dynamic pathology of AD^417^.

The clinical and biological heterogeneity of AD introduces an additional layer of complexity. Patients differ markedly in their age at onset, rate of cognitive decline, and treatment responsiveness. These differences result from a combination of intrinsic factors, such as genetic background, sex, and immune system variability, as well as extrinsic influences including dietary patterns, comorbid conditions, and environmental exposures^418^^,^^419^. Such heterogeneity can obscure relevant molecular signals and hinder the development of AI models that generalize across populations. For instance, machine learning classifiers trained on heterogeneous datasets may average out critical features associated with specific subtypes, thereby reducing both model accuracy and clinical utility^420^.

To address these challenges, advanced patient stratification strategies are increasingly employed to counteract overgeneralization within heterogeneous AD cohorts. Unsupervised learning techniques such as hierarchical clustering, k-means, and latent class analysis applied to integrated multi-omics datasets have revealed molecularly distinct AD subgroups characterized by divergent patterns of neuroinflammation, metabolic dysregulation, and synaptic dysfunction. For example, similarity network fusion analyses of brain tissue have identified clusters associated with immune activation, mitochondrial impairment, and lipid metabolism abnormalities^352^. Other machine learning approaches integrating high-throughput omics data have delineated four distinct molecular profiles that correspond to variability in cognitive function and neuropathological burden^58^. Blood-based transcriptomic analyses have similarly distinguished immune-dominant and metabolic-dominant subgroups, each associated with unique clinical trajectories and therapeutic responses^421^. Stratification into such biologically meaningful subtypes enhances the predictive accuracy of clinical milestones. For instance, identifying molecular subtypes within mild cognitive impairment cohorts improves the prediction of conversion to dementia compared to unstratified models^273^. These stratified approaches provide a foundation for precision medicine by aligning therapeutic interventions with the molecular context of individual patients.

A major limitation to advancing this field is the scarcity of longitudinal multi-omics datasets. Most available studies are cross-sectional in nature, providing static snapshots of disease. Post-mortem multi-omics investigations, for instance, often rely on pseudotemporal inference due to the absence of repeated measurements^35^. Reviews of multi-omics analyses in brain tissue have emphasized the importance of time-series data in capturing the dynamic evolution of molecular and clinical features^397^. Although some imaging-focused cohorts incorporate longitudinal clinical assessments that support predictions of disease conversion^362^, the integration of repeated multi-omics sampling over time remains rare.

In conclusion, enhancing the biological interpretability and translational potential of AI-based multi-omics research in AD requires a more nuanced approach to patient diversity. This includes applying data-driven clustering methods to define biologically relevant subtypes, expanding these frameworks to identify subgroup-specific biomarkers and therapeutic targets, and establishing longitudinal, multi-modal cohorts that combine serial omics profiling with follow-up neuroimaging and clinical evaluations. Such efforts will reduce the risk of model overfitting, improve the identification of meaningful biological mechanisms, and support the development of individualized treatment strategies that align with the complex molecular landscape of AD^361^.

### 6.3 Clinical challenges

The translation of AI-based multi-omics research into clinical practice for AD presents a range of significant challenges spanning methodological, interpretative, infrastructural, and regulatory domains^405^^,^^422^. Reproducibility is a primary concern. While AI models often achieve high accuracy in controlled research settings, their performance frequently declines in real-world clinical environments. Many studies rely on datasets with tens of thousands of molecular and imaging features derived from relatively small cohorts. For instance, reliable detection of moderate molecular effects in AD requires at least 500 paired omics and imaging samples, yet most studies fall short of this benchmark^423^. This imbalance contributes to overfitting and limits generalizability. To address these limitations, best practices now emphasize the use of nested or repeated k-fold cross-validation, strict separation of hold-out test sets, and independent replication in external validation cohorts such as ADNI, Australian Imaging Biomarkers and Lifestyle (AIBL), and AddNeuroMed. Benchmarking efforts, including the DREAM Alzheimer's Big Data Challenge, have revealed that many models perform well on internal data but fail to generalize to unseen datasets, highlighting the necessity of transparent head-to-head comparisons against simple baseline models^408^.

Interpretability is another key barrier to clinical adoption. The complexity of machine learning algorithms, particularly deep learning models, often makes it difficult for clinicians to understand or trust the predictions. To enhance transparency and usability, XAI techniques tailored to biomedical applications are increasingly integrated into model workflows. Methods such as SHAP, integrated gradients, attention-based visualization, and counterfactual reasoning clarify the influence of specific features, including genetic variants and imaging measures, on model predictions. Recent developments include interactive dashboards that allow clinicians to explore how individual variables contribute to patient-specific risk scores^364^^,^^424^. These tools help translate complex outputs into interpretable formats, improving clinician engagement and decision-making. The use of user-centered design (UCD) in XAI development ensures that explanations are presented in clinically meaningful ways. Studies involving clinician interviews, prototyping, and usability testing demonstrate that well-designed interfaces significantly improve comprehension and perceived utility^425^. Embedding these tools within electronic health records or clinical decision support systems further facilitates adoption. Mixed-method evaluations show that clinicians are more confident and more likely to follow AI-generated recommendations when supported by transparent explanations such as SHAP plots^426^^,^^427^. Evidence from prospective deployments reinforces this trend. For example, an XAI-enhanced early warning system improved diagnostic accuracy for acute deterioration, while an interpretable decision support tool for remote strep throat diagnosis increased clinician performance and highlighted the importance of intelligible feedback for sustained use^427^^,^^428^. While interpretability is increasingly recognized as essential for the clinical integration of AI, further prospective and real-world studies are needed to quantify its impact on patient outcomes and define implementation best practices^429^^,^^430^.

Infrastructure and regulatory limitations further complicate the clinical implementation of AI-driven approaches. Effective deployment requires secure, scalable storage, strong cybersecurity measures, and seamless integration with existing electronic health record systems. Many healthcare institutions, particularly in low-resource settings, lack these capabilities. To address these gaps, ongoing efforts focus on developing standardized data formats and improving system interoperability to support data exchange and model deployment^431^. Regulatory approval also demands rigorous demonstration of clinical utility in prospective, multi-center trials. Guidelines such as CONSORT-AI and SPIRIT-AI emphasize the need for protocol pre-registration, comprehensive reporting of preprocessing steps and model parameters, and continuous post-deployment monitoring. Updating “model cards”, which are structured documents that detail model performance, version history, and known limitations, is becoming standard practice in regulatory submissions^432^.

Bridging the cultural and knowledge divide between clinicians and data scientists is also essential. This requires both institutional investment in cross-disciplinary collaboration and individual training in AI literacy. Recent competency frameworks recommend that clinicians acquire foundational and applied knowledge in data provenance, algorithm behavior, and uncertainty communication prior to deploying AI tools in clinical settings. Online training programs, such as the Radiology AI Literacy curriculum, now adopted by over 500 practicing radiologists, demonstrate strong demand for such education^433^. Conference sessions, including the “Empowering Healthcare Professionals Through AI Literacy” workshop at HIMSS 2025, further support skills development and help align expectations regarding AI capabilities. Organizational studies show that models co-designed by interdisciplinary teams achieve greater clinical accuracy and user acceptance compared to those developed in disciplinary silos^434^. This insight has informed institutional initiatives like the Houston Methodist–Rice University Digital Health Institute, which offers a collaborative environment for model development, validation, and deployment. Governance frameworks that incorporate ethics boards and patient advocacy representatives throughout the development lifecycle also play a critical role in ensuring transparency, addressing algorithmic bias, and promoting equitable use of AI systems^412^. Together, these initiatives foster a virtuous cycle of trust, interpretability, and clinical effectiveness, accelerating the safe and meaningful integration of AI into real-world healthcare environments.

In summary, the successful clinical translation of AI-based multi-omics approaches in AD requires a rigorous study design with sufficient sample sizes, robust internal and external validation, transparent benchmarking, interpretable and clinician-friendly model outputs, interoperable infrastructure, and sustained interdisciplinary collaboration. These strategies are essential for transforming AI-based multi-omics innovations from research tools into clinically actionable solutions that enhance diagnosis, stratification, and therapeutic decision-making in AD.

## 7 Conclusions and perspectives

AI-driven multi-omics research presents transformative potential for AD, offering innovative avenues for early diagnosis, individualized treatment, and accelerated drug discovery. By integrating genomic, transcriptomic, proteomic, and metabolomic data, AI models enable a comprehensive understanding of the molecular complexity underlying AD, elucidating the combined influence of genetic, environmental, and biological factors. These integrative approaches facilitate the identification of novel biomarkers for early detection, allowing timely interventions prior to the onset of clinical symptoms, a critical window for effective disease management. Furthermore, AI empowers precision medicine by aligning therapeutic strategies with individual molecular profiles, moving beyond one-size-fits-all treatments toward more targeted, effective, and safer interventions. The capacity of AI models to learn from continuously evolving datasets further enhances their ability to adapt to disease progression, supporting dynamic and optimized therapeutic decisions throughout the clinical course.

To fully realize the promise of AI-driven multi-omics in AD, several key challenges must be addressed while seizing emerging opportunities. Standardization and harmonization of data acquisition, processing, and annotation across diverse platforms and populations are essential for ensuring model comparability and reproducibility. The development of large-scale, well-curated, and demographically diverse datasets that reflect variations in ethnicity, age, and disease stage is crucial for improving model robustness, generalizability, and fairness. Achieving this will require international collaboration and the establishment of secure, ethically governed data-sharing frameworks to mitigate data fragmentation and enable validation across independent cohorts. Furthermore, the adoption of standardized reporting guidelines and evaluation protocols will facilitate transparency and enable meaningful cross-study comparisons. Employing a range of performance metrics, particularly those suited to imbalanced datasets common in AD, will allow a more comprehensive assessment of model performance.

Interpretability remains a critical requirement for clinical translation. The application of XAI techniques that reveal the underlying biological basis of predictions will support not only trust and clinical usability but also the discovery of actionable molecular pathways and therapeutic targets. Advances in computational methodologies, including deep learning, network-based modeling, and causal inference, continue to expand the ability to capture nonlinear, dynamic interactions in disease processes. Incorporating additional data modalities, such as epigenomics, microbiome composition, neuroimaging, and longitudinal electronic health record data, will further strengthen predictive capabilities and enable refined patient stratification.

Ethical and regulatory considerations are integral to the responsible implementation of AI in AD research and care. Ensuring data privacy, informed consent, and strong cybersecurity practices remains essential. Addressing algorithmic bias proactively is critical to avoid perpetuating or exacerbating health disparities. Regulatory frameworks, including GDPR, HIPAA, CONSORT-AI, and SPIRIT-AI, are evolving to require greater transparency, interpretability, and continuous post-deployment performance monitoring. Demonstrating clinical value through prospective, multi-center trials with patient-centered outcomes is increasingly necessary for regulatory approval and clinical adoption.

Importantly, AI offers substantial opportunities to accelerate drug discovery by identifying molecular targets, predicting drug responses, and integrating systems pharmacology data. Adaptive clinical trials guided by real-time AI predictions can improve patient selection and dynamically adjust treatment protocols, thereby increasing trial efficiency and success rates.

In conclusion, the future of AI-driven multi-omics in AD will be shaped by rigorous data standards, ethical integrity, interpretable modeling, and robust clinical validation. Together, these efforts will advance a new paradigm for earlier diagnosis, personalized therapy, and more effective interventions, ultimately improving outcomes and quality of life for individuals affected by AD.

## Author contributions

Fang Ren: Writing—review & editing. Jing Wei: Writing—original draft. Qingxin Chen: Writing—original draft. Mengling Hu: Investigation, Writing—original draft. Lu Yu: Investigation, Visualization. Jianing Mi: Visualization. Xiaogang Zhou: Visualization. Dalian Qin: Investigation, Visualization. Jianming Wu: Data curation, Writing—review & editing. Anguo Wu: Funding acquisition, Software, Supervision, Writing— review & editing.

## Conflicts of interest

The authors declare that they have no competing interests.
